# Supplementary material for: Sexual dimorphism in shell size of the land snail Leptopoma perlucidum (Caenogastropoda: Cyclophoridae)
Source: PeerJ. 2022 May 27;10:e13501. doi: 10.7717/peerj.13501 (PMC9150688; doi:10.7717/peerj.13501)
Supplement: File S3 — Tables of frequentist and Bayesian t-test, respectively, on the effects of sexes on (1) SH—Shell height; (2) SW—Shell width; (3) AH—Aperture height; (4) AW—Aperture width; and (5) SpH—Shell spire height. Geometric morphometric analysis on shell shape comparison: (1) Eigenvalues for principal component analysis (PCA) for shell shape of Leptopoma perlucidum; (2) Procrustes ANOVA table for shape differences between opposites sexes, locations, and interactions between them. [file peerj-10-13501-s003.docx]

**Table S1.** Full results (JASP output table) of assumption testing and frequentist t-test on the effects of sex on shell size of the land snail *Leptopoma perlucidum* from Gaya Island and Tiga Island.

**Gaya Island**

| **Independent Samples T-Test** | | | | | | | | | | |  |
| --- | --- | --- | --- | --- | --- | --- | --- | --- | --- | --- | --- |
|  | | **t** | | **df** | | | **p** | | **Cohen's d** | |  |
| Shell Height |  | 4.363 |  | | 19 |  | < .001 |  | 1.924 |  | |
| Shell Width |  | 4.034 |  | | 19 |  | < .001 |  | 1.779 |  | |
| Aperture Height |  | 3.798 |  | | 19 |  | 0.001 |  | 1.675 |  | |
| Aperture Width |  | 2.607 |  | | 19 |  | 0.017 |  | 1.149 |  | |
| Spire Height |  | 3.928 |  | | 19 |  | < .001 |  | 1.732 |  | |
|  | | | | | | | | | | |  |
| *Note.*  Student's t-test. | | | | | | | | | | |  |

**Assumption Checks**

| **Test of Normality (Shapiro-Wilk)** | | | | | | | |
| --- | --- | --- | --- | --- | --- | --- | --- |
|  | |  | | **W** | | **p** | |
| Shell Height |  | female |  | 0.915 |  | 0.352 |  |
|  |  | male |  | 0.955 |  | 0.706 |  |
| Shell Width |  | female |  | 0.947 |  | 0.658 |  |
|  |  | male |  | 0.924 |  | 0.317 |  |
| Aperture Height |  | female |  | 0.887 |  | 0.186 |  |
|  |  | male |  | 0.861 |  | 0.051 |  |
| Aperture Width |  | female |  | 0.983 |  | 0.976 |  |
|  |  | male |  | 0.928 |  | 0.363 |  |
| Spire Height |  | female |  | 0.979 |  | 0.961 |  |
|  |  | male |  | 0.940 |  | 0.500 |  |
|  | | | | | | | |
| *Note.*  Significant results suggest a deviation from normality. | | | | | | | |

| **Test of Equality of Variances (Levene's)** | | | | | | | |
| --- | --- | --- | --- | --- | --- | --- | --- |
|  | | **F** | | **df** | | **p** | |
| Shell Height |  | 0.096 |  | 1 |  | 0.760 |  |
| Shell Width |  | 1.788 |  | 1 |  | 0.197 |  |
| Aperture Height |  | 0.368 |  | 1 |  | 0.551 |  |
| Aperture Width |  | 0.278 |  | 1 |  | 0.604 |  |
| Spire Height |  | 0.590 |  | 1 |  | 0.452 |  |
|  | | | | | | | |

| **Tiga Island**  **Independent Samples T-Test** | | | | | | | | | |
| --- | --- | --- | --- | --- | --- | --- | --- | --- | --- |
|  | | t | | df | | p | | Cohen's d | |
| Shell Height |  | 3.941 |  | 61 |  | < .001 |  | 0.994 |  |
| Shell Width |  | 4.770 |  | 61 |  | < .001 |  | 1.203 |  |
| Aperture Height |  | 3.716 |  | 61 |  | < .001 |  | 0.937 |  |
| Aperture Width |  | 4.753 |  | 61 |  | < .001 |  | 1.199 |  |
| Spire Height |  | 2.971 |  | 61 |  | 0.004 |  | 0.749 |  |
|  | | | | | | | | | |
| *Note.*  Student's t-test. | | | | | | | | | |

Assumption Checks

| Test of Normality (Shapiro-Wilk) | | | | | | | |
| --- | --- | --- | --- | --- | --- | --- | --- |
|  | |  | | W | | p | |
| Shell Height |  | female |  | 0.982 |  | 0.842 |  |
|  |  | male |  | 0.869 |  | 0.002 |  |
| Shell Width |  | female |  | 0.936 |  | 0.052 |  |
|  |  | male |  | 0.895 |  | 0.006 |  |
| Aperture Height |  | female |  | 0.935 |  | 0.047 |  |
|  |  | male |  | 0.941 |  | 0.099 |  |
| Aperture Width |  | female |  | 0.947 |  | 0.109 |  |
|  |  | male |  | 0.944 |  | 0.119 |  |
| Spire Height |  | female |  | 0.962 |  | 0.302 |  |
|  |  | male |  | 0.942 |  | 0.100 |  |
|  | | | | | | | |
| *Note.*  Significant results suggest a deviation from normality. | | | | | | | |

| Test of Equality of Variances (Levene's) | | | | | | | |
| --- | --- | --- | --- | --- | --- | --- | --- |
|  | | F | | df | | p | |
| Shell Height |  | 0.003 |  | 1 |  | 0.953 |  |
| Shell Width |  | 0.072 |  | 1 |  | 0.790 |  |
| Aperture Height |  | 0.252 |  | 1 |  | 0.617 |  |
| Aperture Width |  | 0.344 |  | 1 |  | 0.560 |  |
| Spire Height |  | 0.730 |  | 1 |  | 0.396 |  |
|  | | | | | | | |

**Table S2.** Full results (JASP output table) of Bayesian t-test on the effects of sex on shell size of the land snail *Leptopoma perlucidum* from Gaya Island and Tiga Island.

**Gaya Island**

| **Bayesian Independent Samples T-Test** | | | | | |
| --- | --- | --- | --- | --- | --- |
|  | | **BF₁₀** | | **error %** | |
| Shell Height |  | 72.548 |  | 1.353e -7 |  |
| Shell Width |  | 39.437 |  | 1.725e -4 |  |
| Aperture Height |  | 25.685 |  | 3.792e -4 |  |
| Aperture Width |  | 3.484 |  | 3.625e -4 |  |
| Spire Height |  | 32.516 |  | 2.818e -6 |  |
|  | | | | | |

**Inferential Plots**

**Shell Height**

**Prior and Posterior**


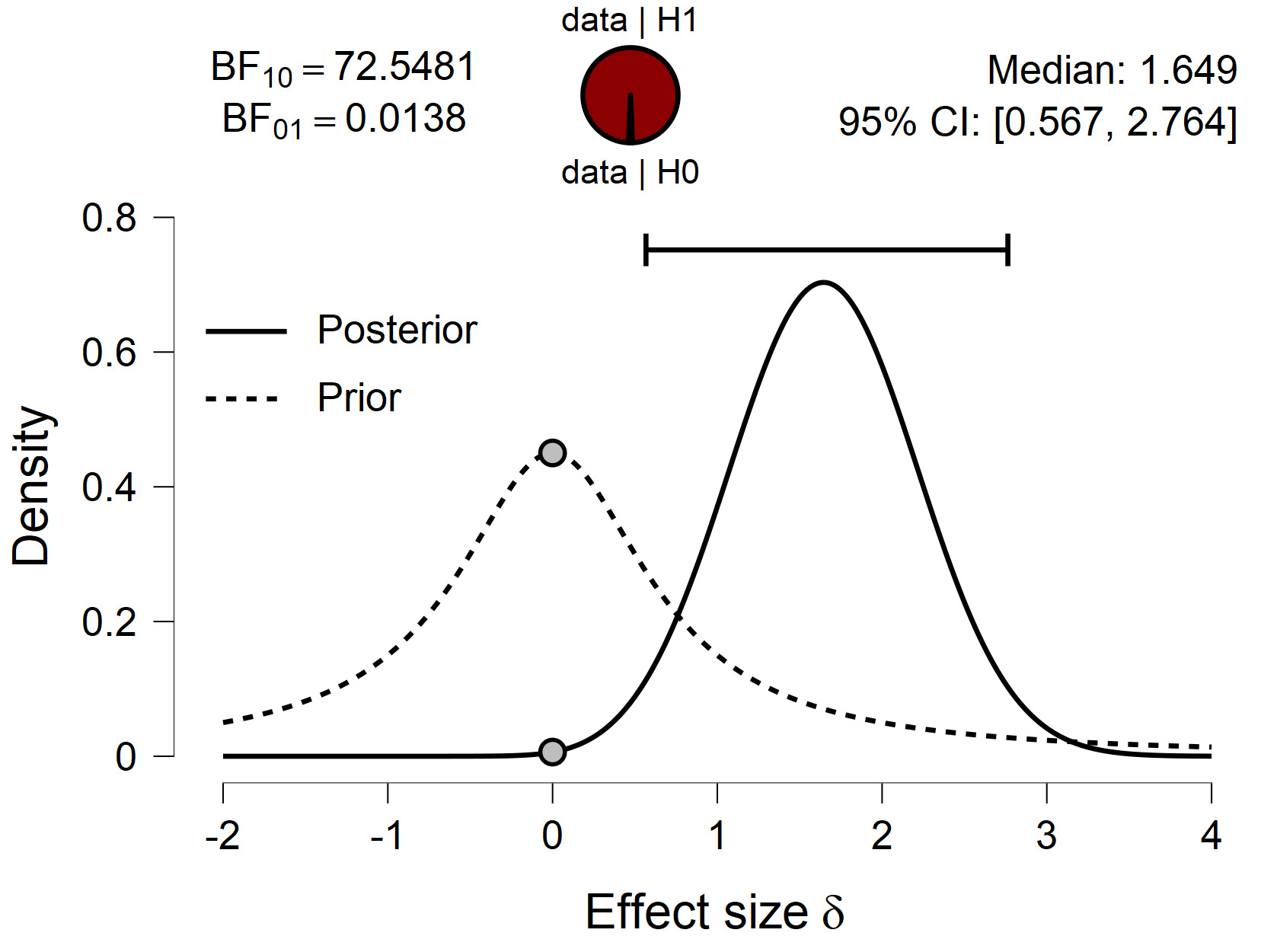


**Bayes Factor Robustness Check**


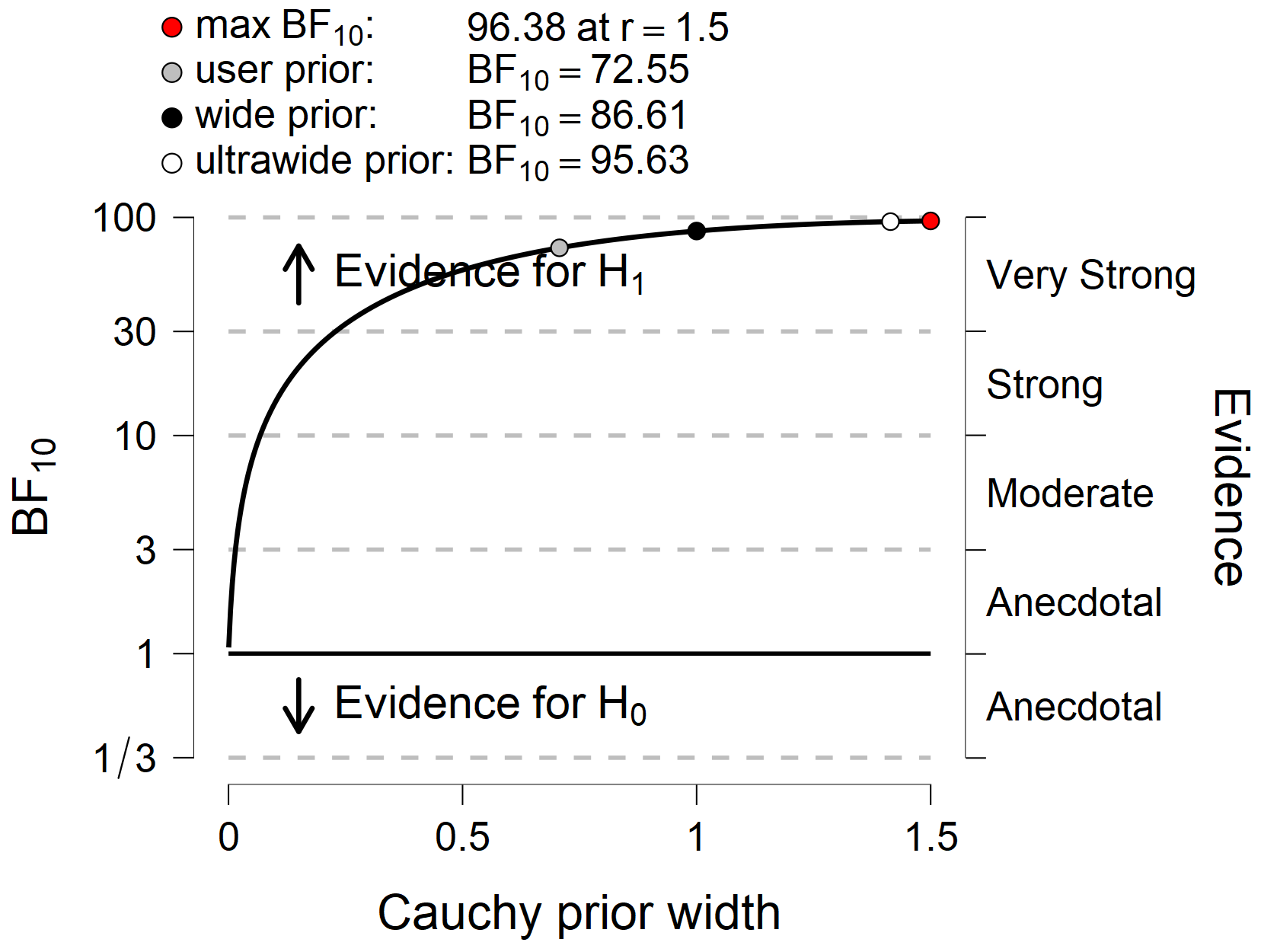


**Shell Width**

**Prior and Posterior**


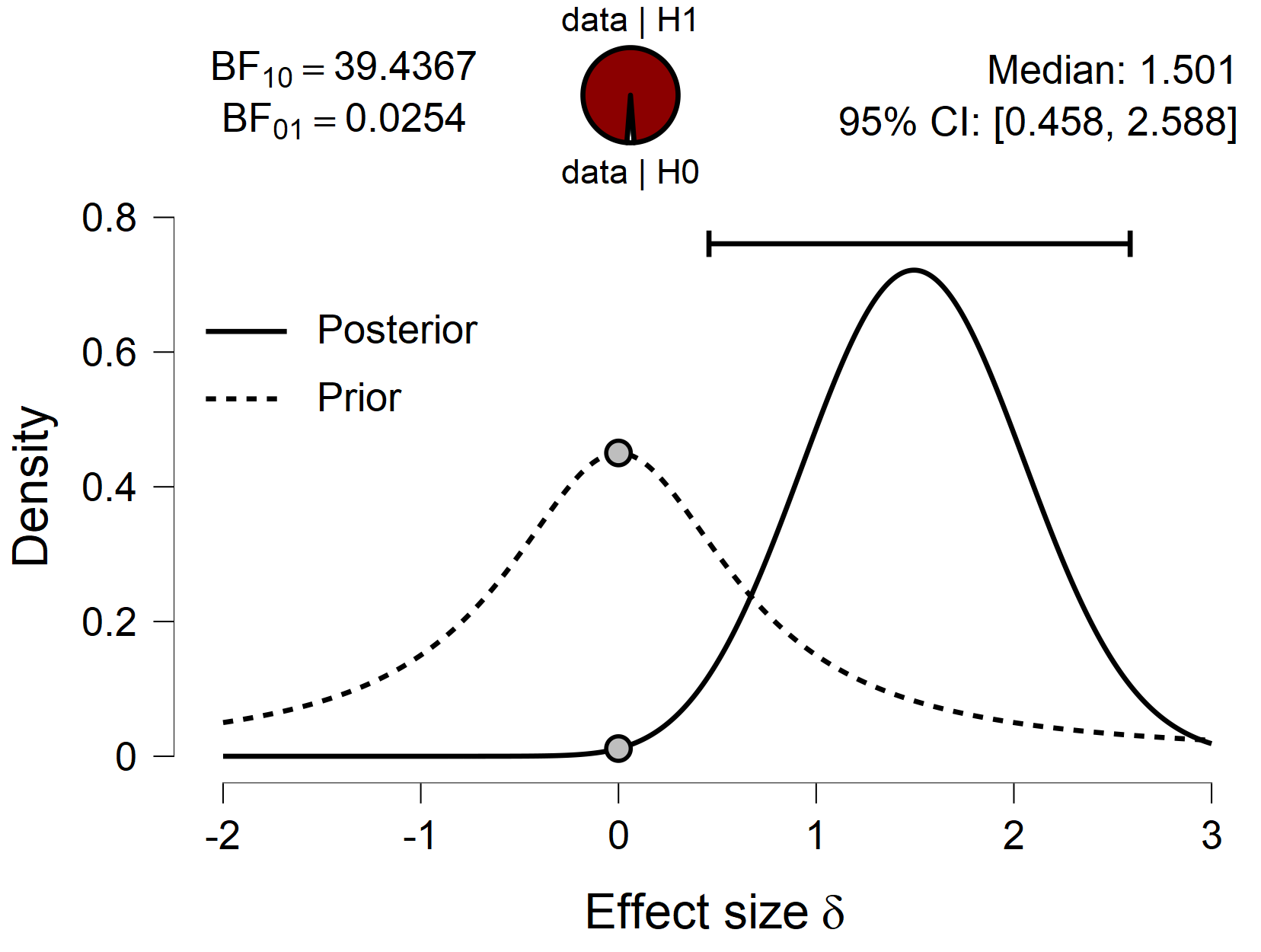


**Bayes Factor Robustness Check**


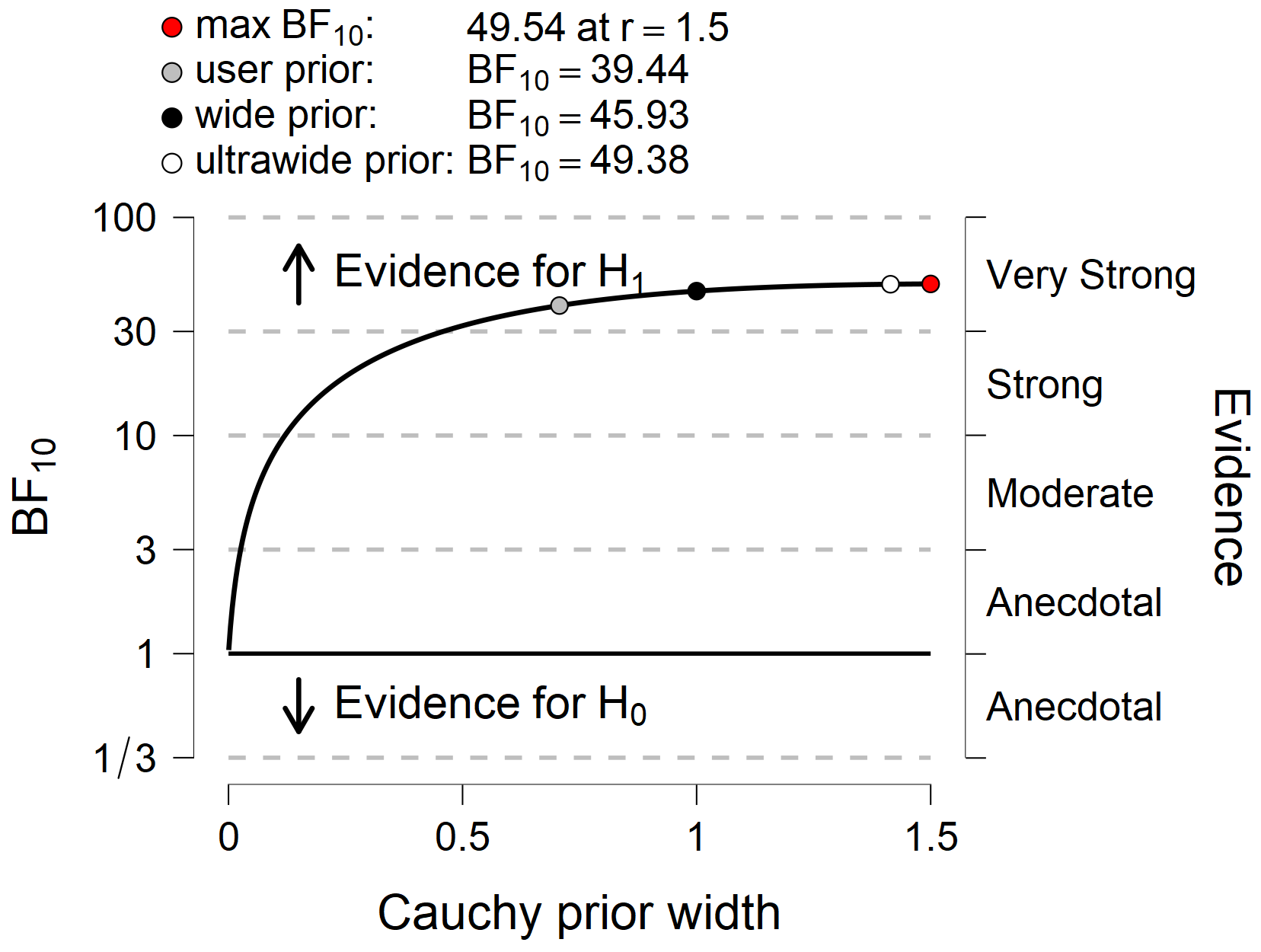


**Aperture Height**

**Prior and Posterior**


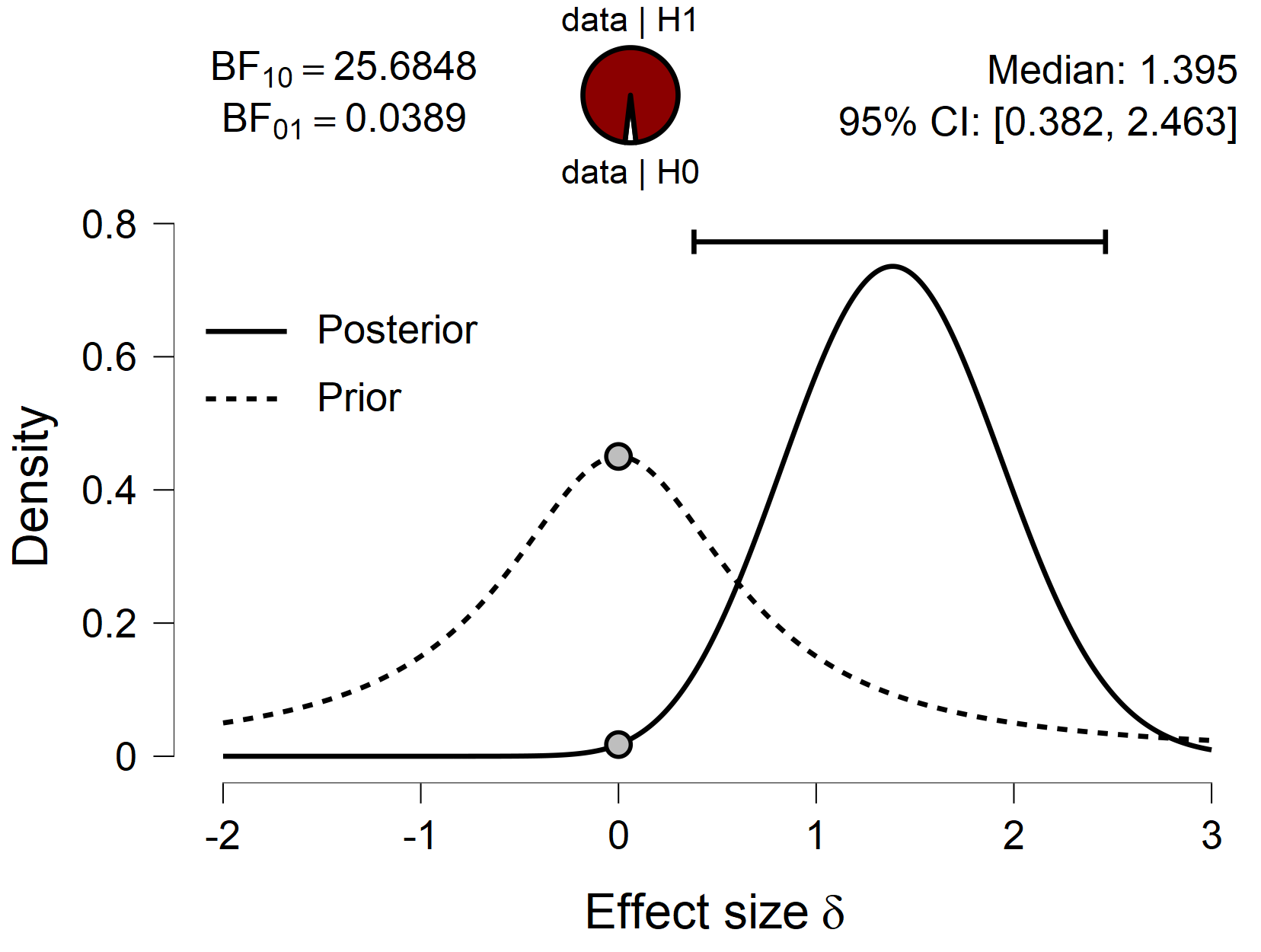


**Bayes Factor Robustness Check**


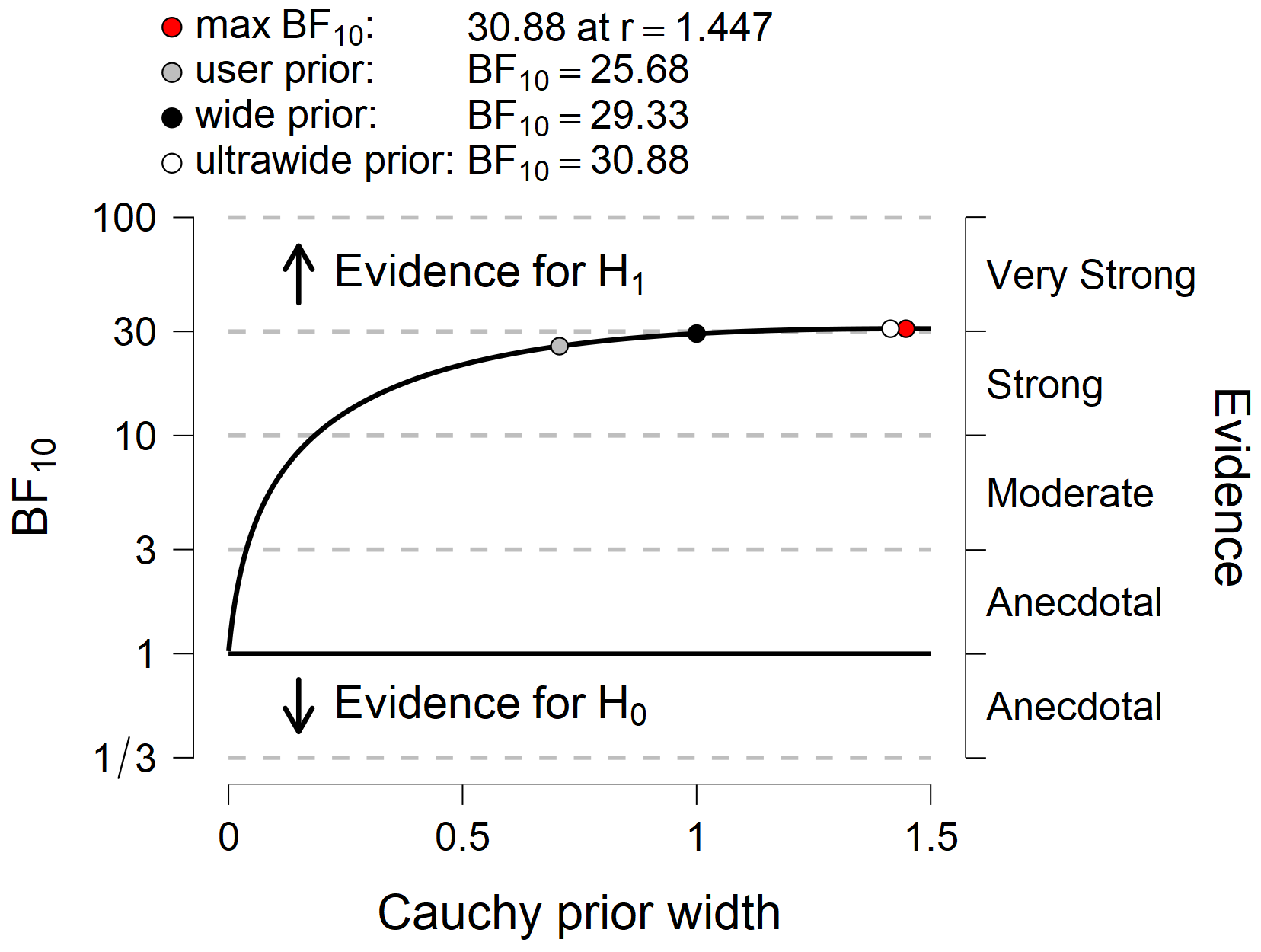


**Aperture Width**

**Prior and Posterior**


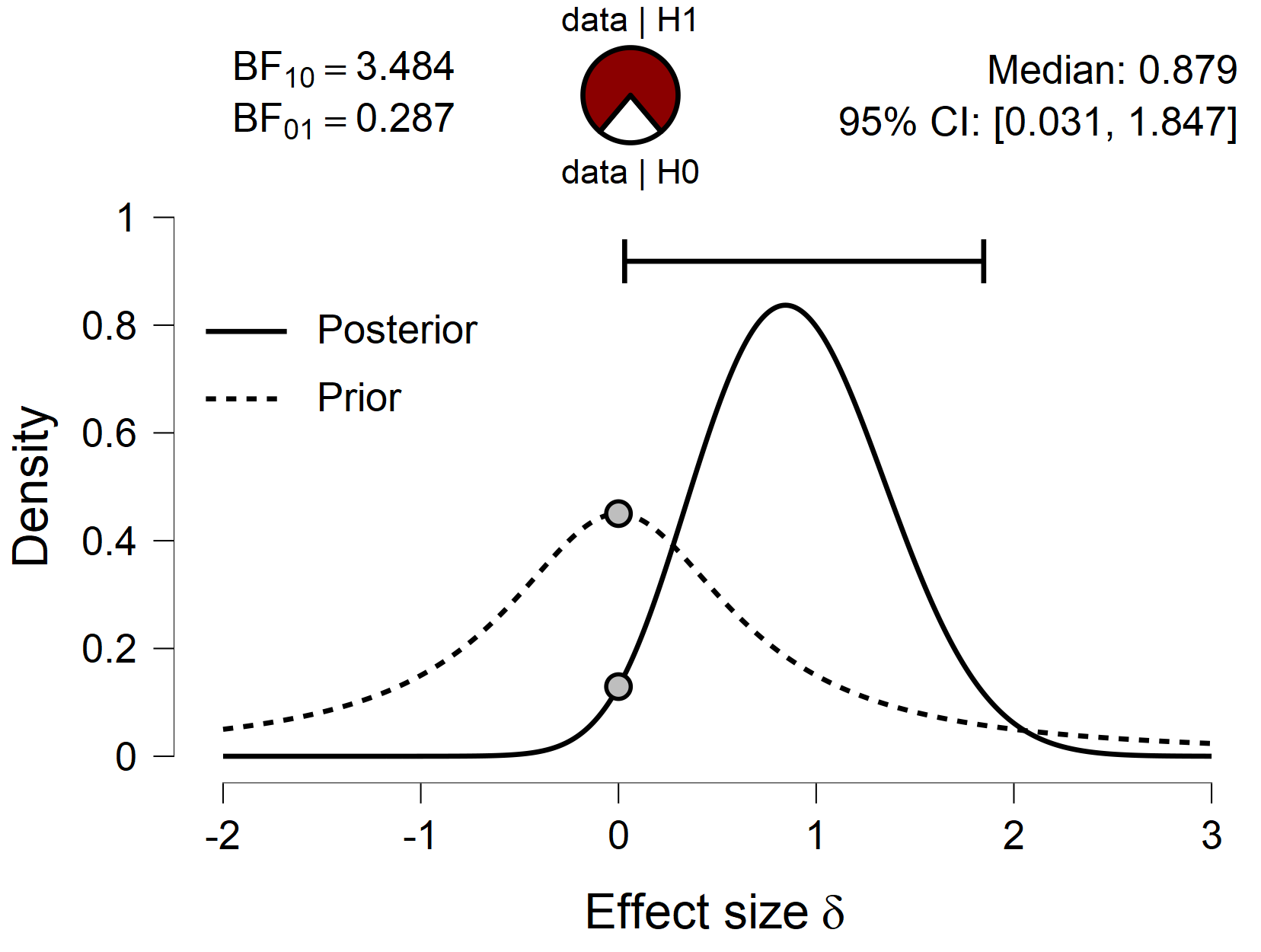


**Bayes Factor Robustness Check**


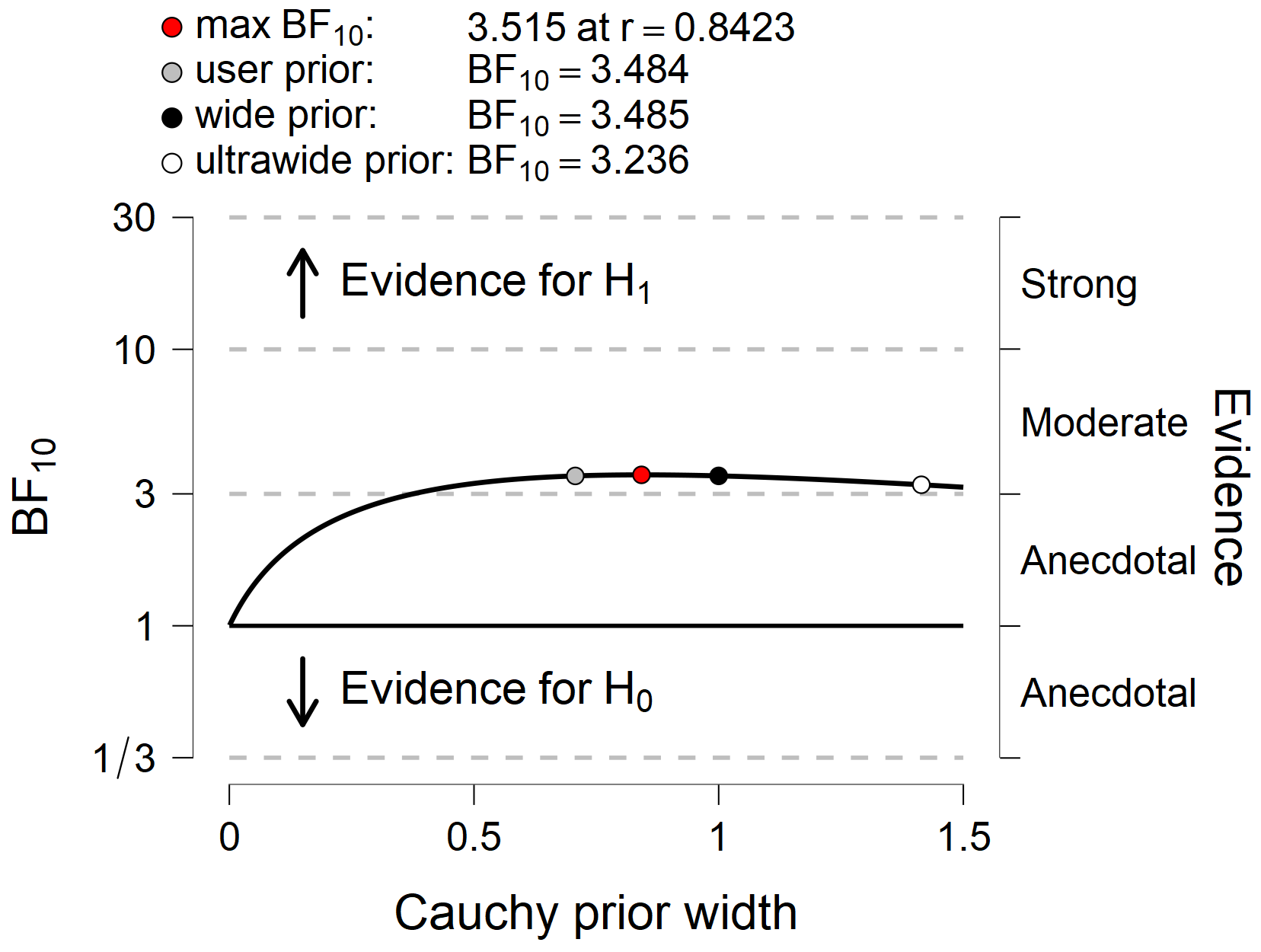


**Spire Height**

**Prior and Posterior**


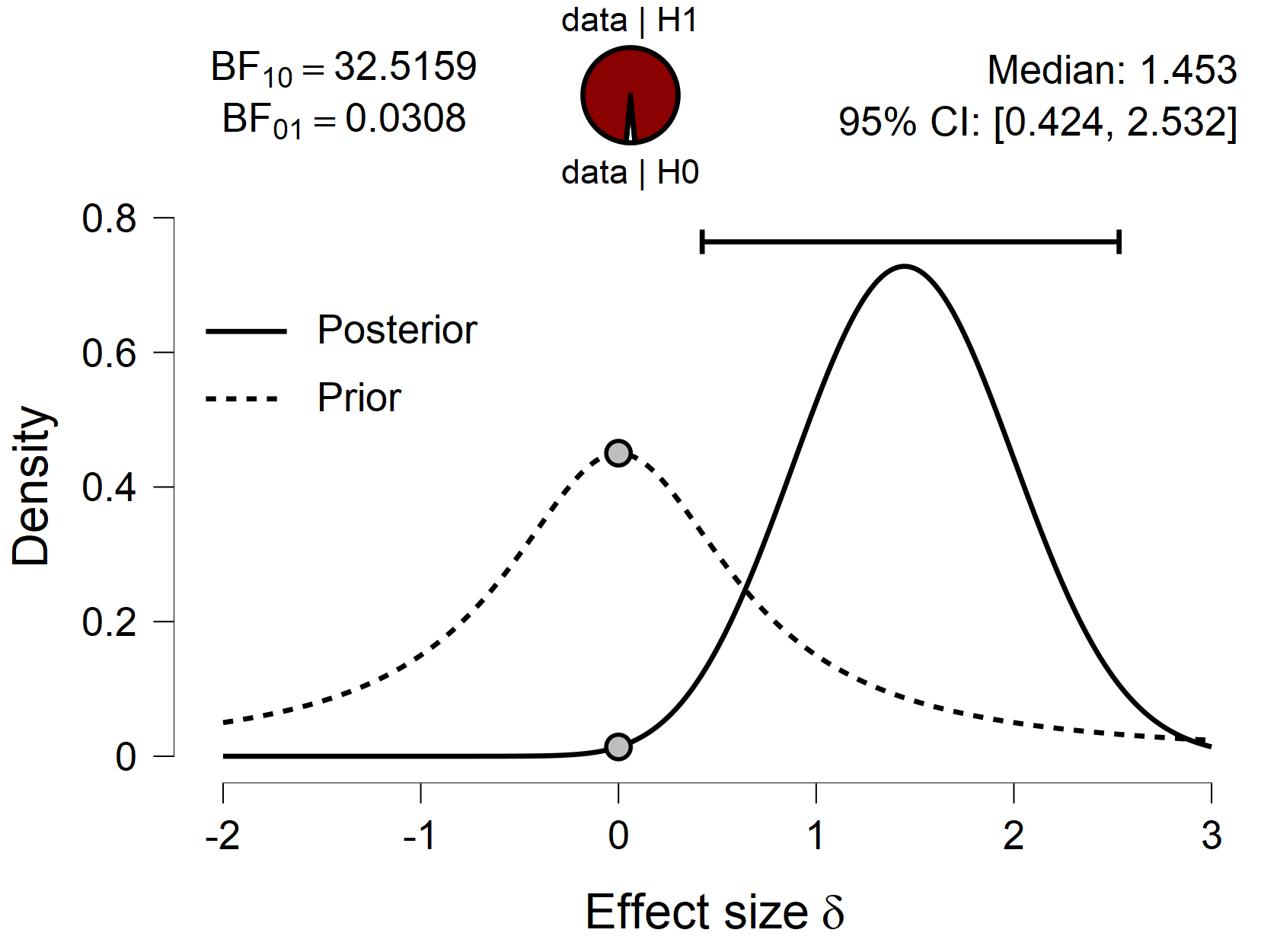


**Bayes Factor Robustness Check**


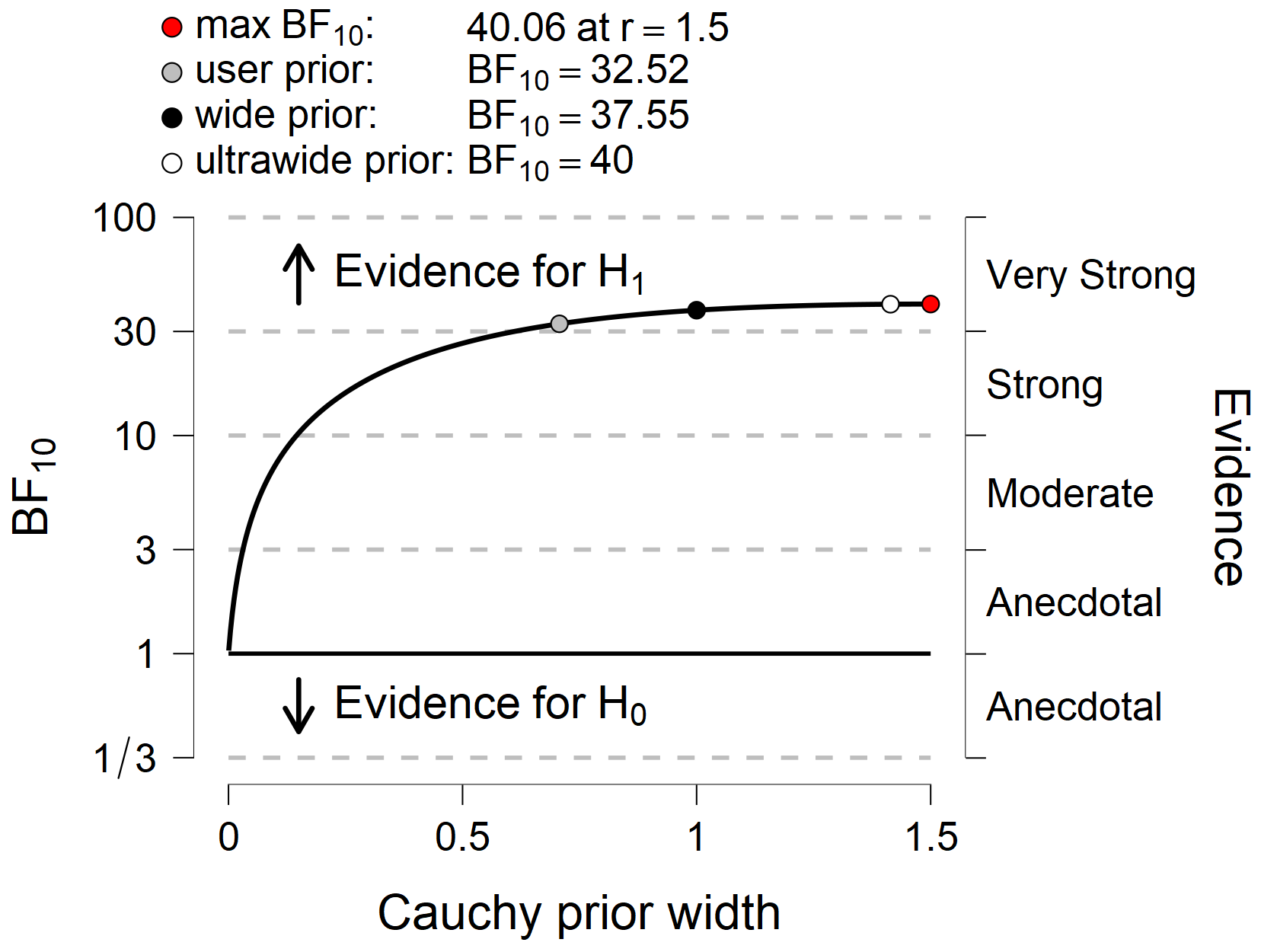


**Tiga Island**

| **Bayesian Independent Samples T-Test** | | | | | |
| --- | --- | --- | --- | --- | --- |
|  | | **BF₁₀** | | **error %** | |
| Shell Height |  | 115.827 |  | 5.570e -7 |  |
| Shell Width |  | 1451.783 |  | 2.785e -8 |  |
| Aperture Height |  | 61.690 |  | 1.422e -6 |  |
| Aperture Width |  | 1374.491 |  | 2.939e -8 |  |
| Spire Height |  | 9.321 |  | 9.651e -4 |  |
|  | | | | | |

### Inferential Plots

#### *Shell Height*

##### Prior and Posterior


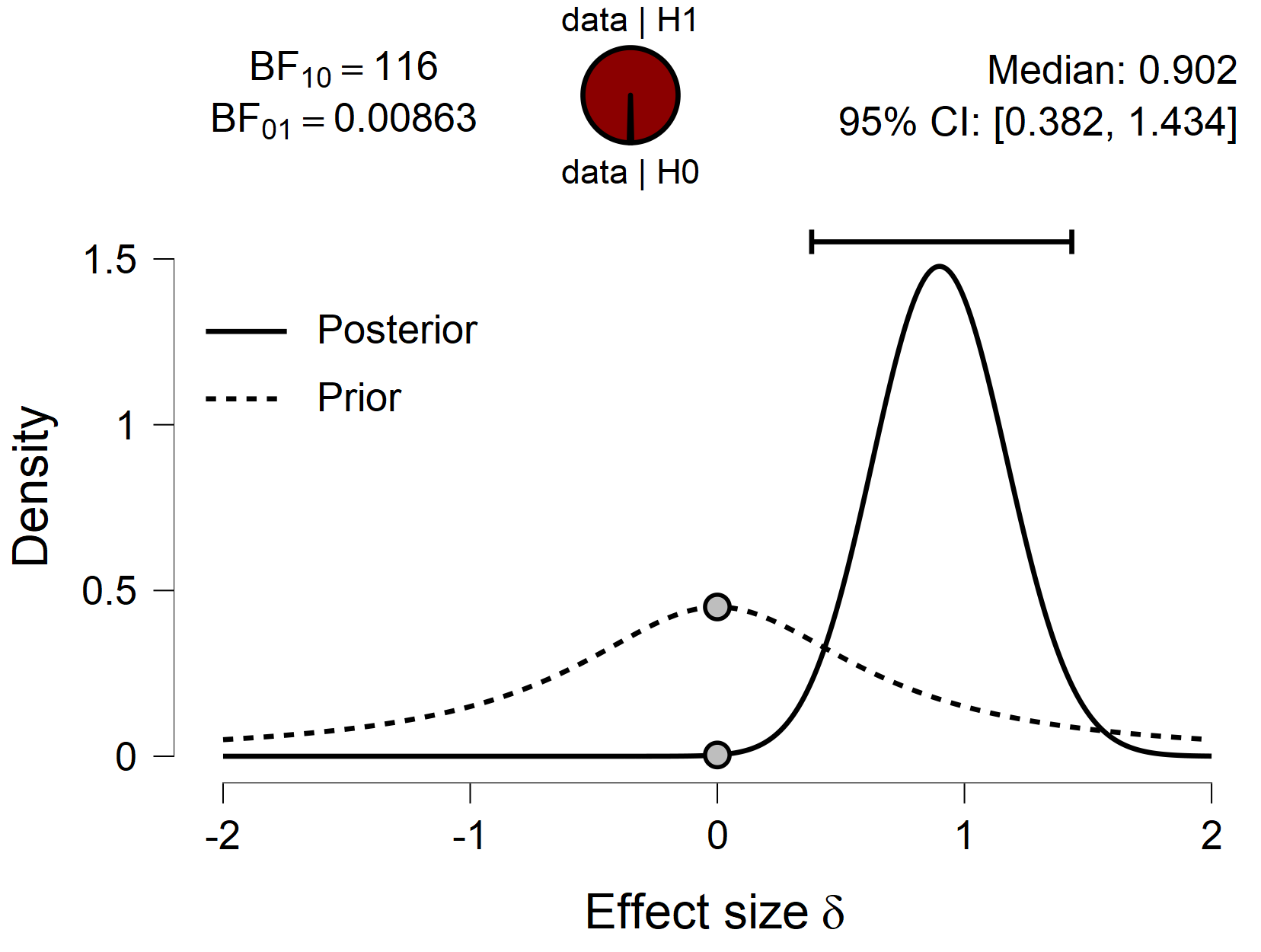


##### Bayes Factor Robustness Check


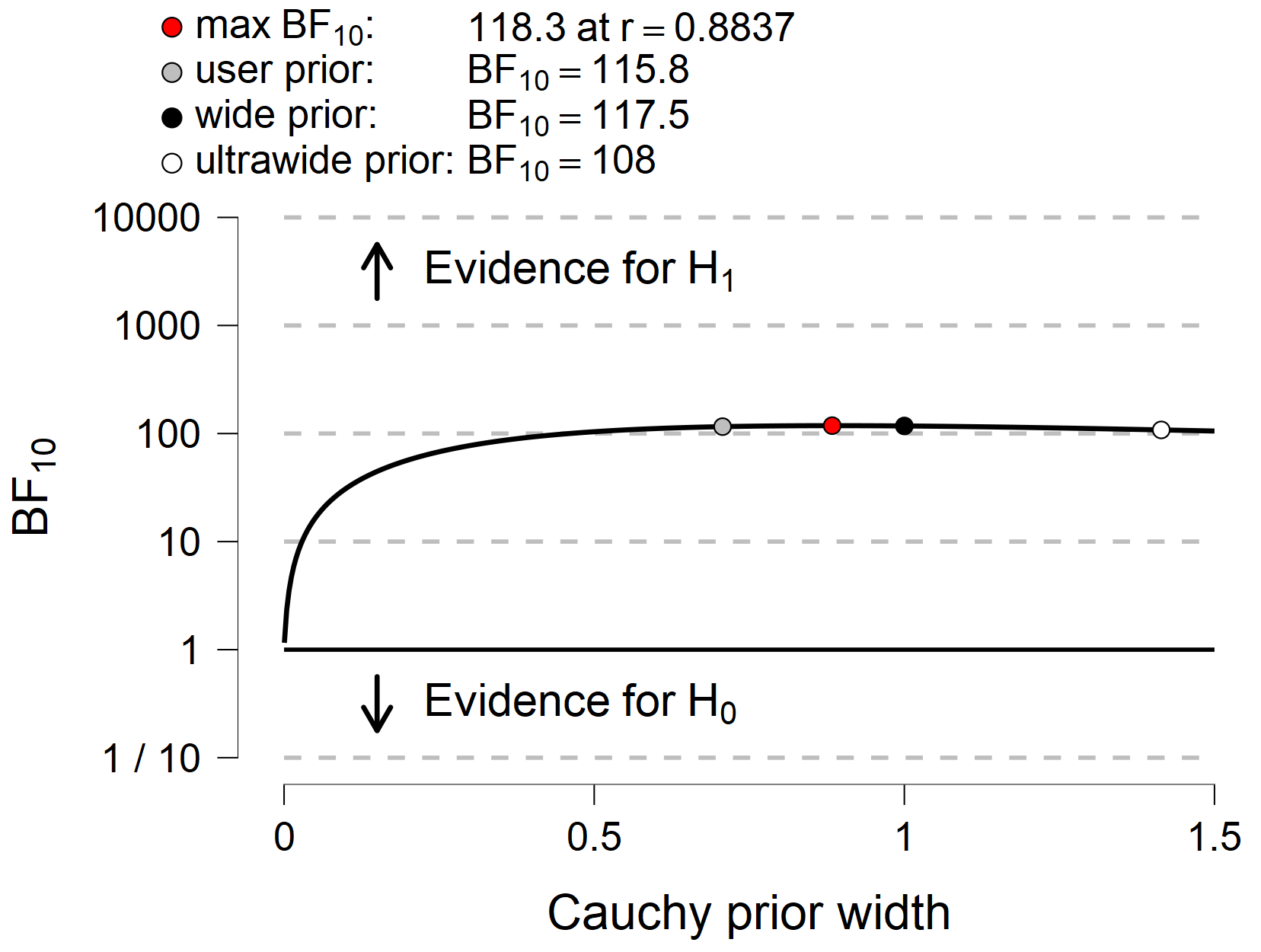


#### *Shell Width*

##### Prior and Posterior


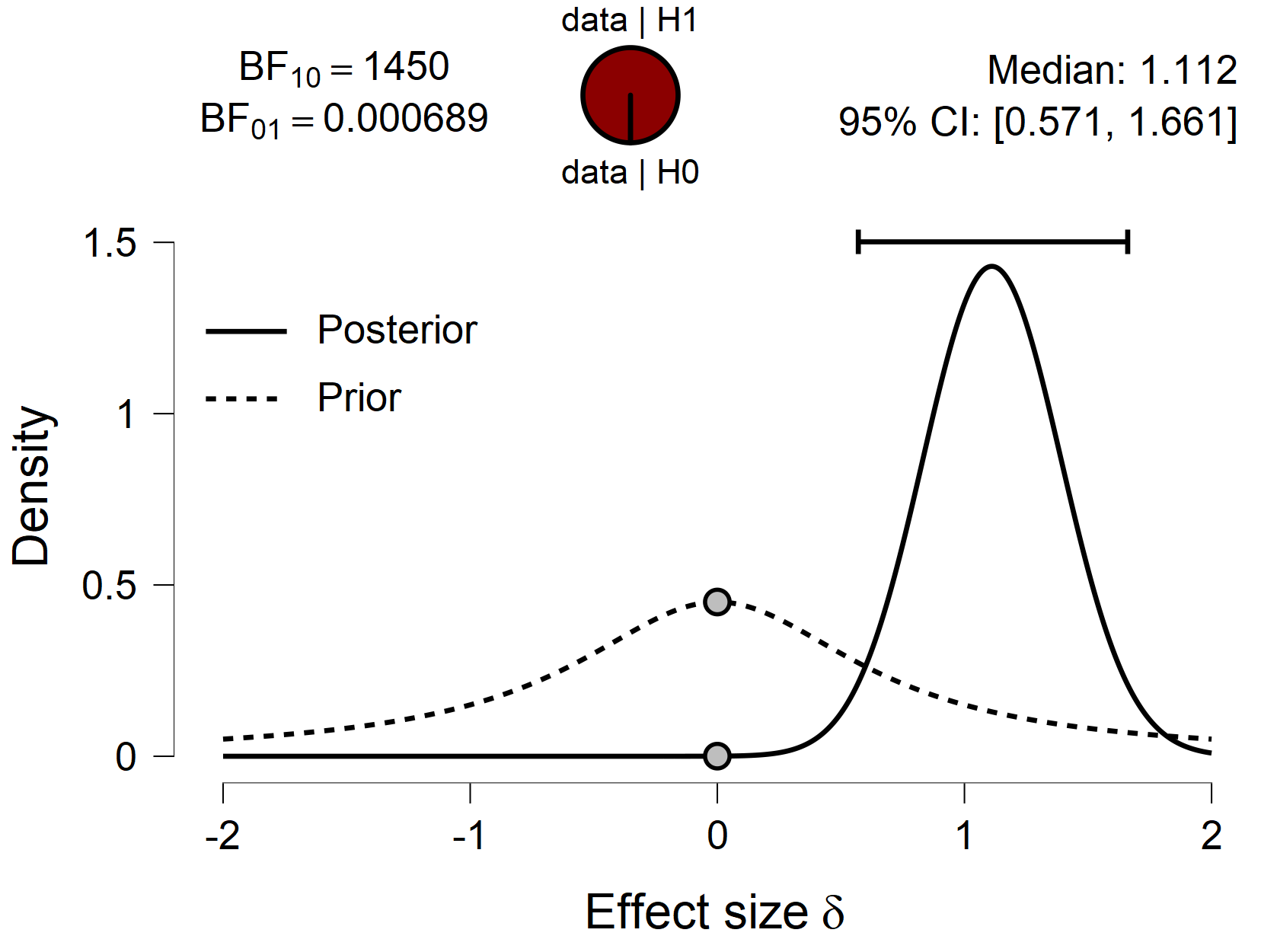


##### Bayes Factor Robustness Check


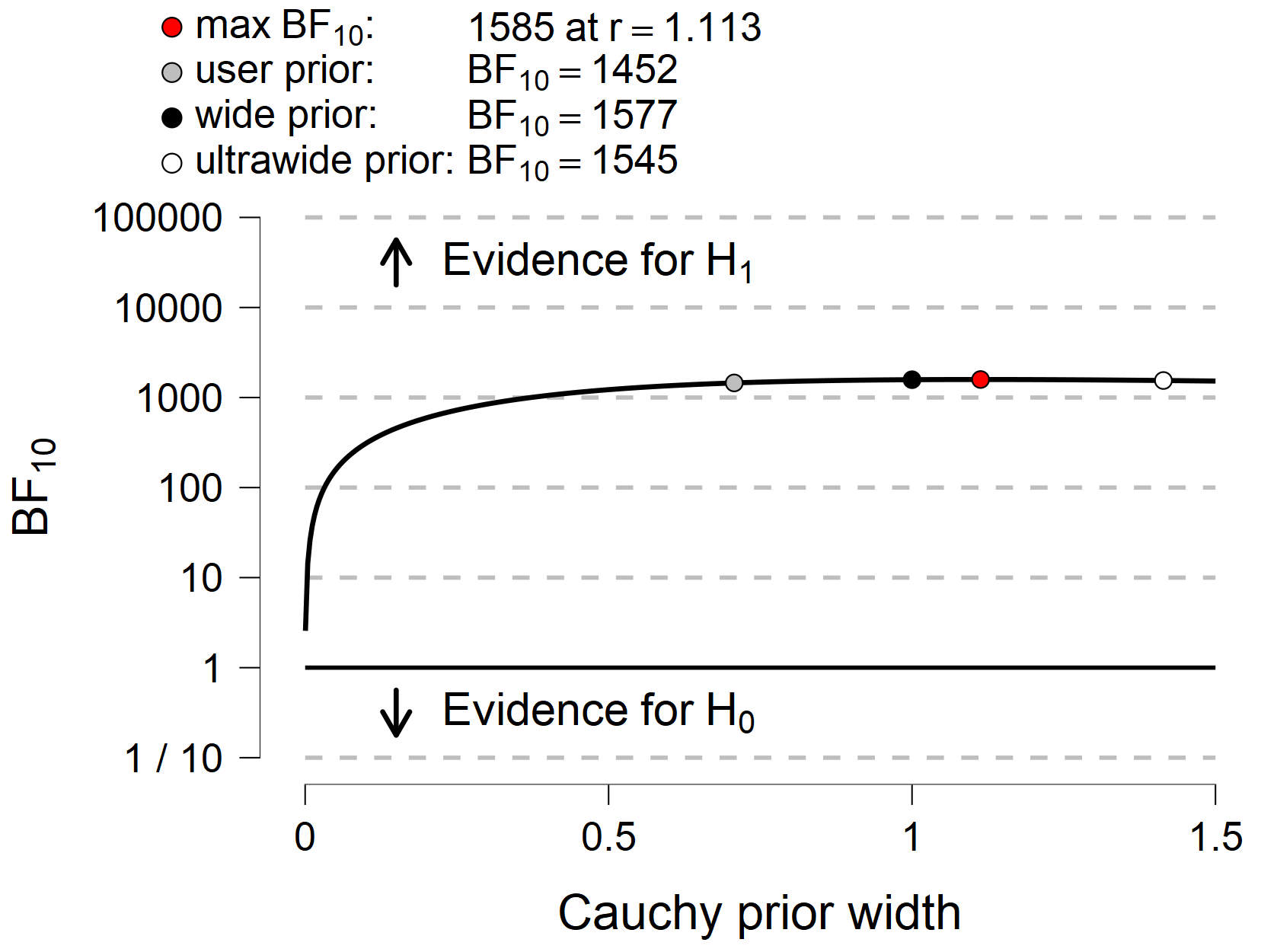


#### *Aperture Height*

##### Prior and Posterior


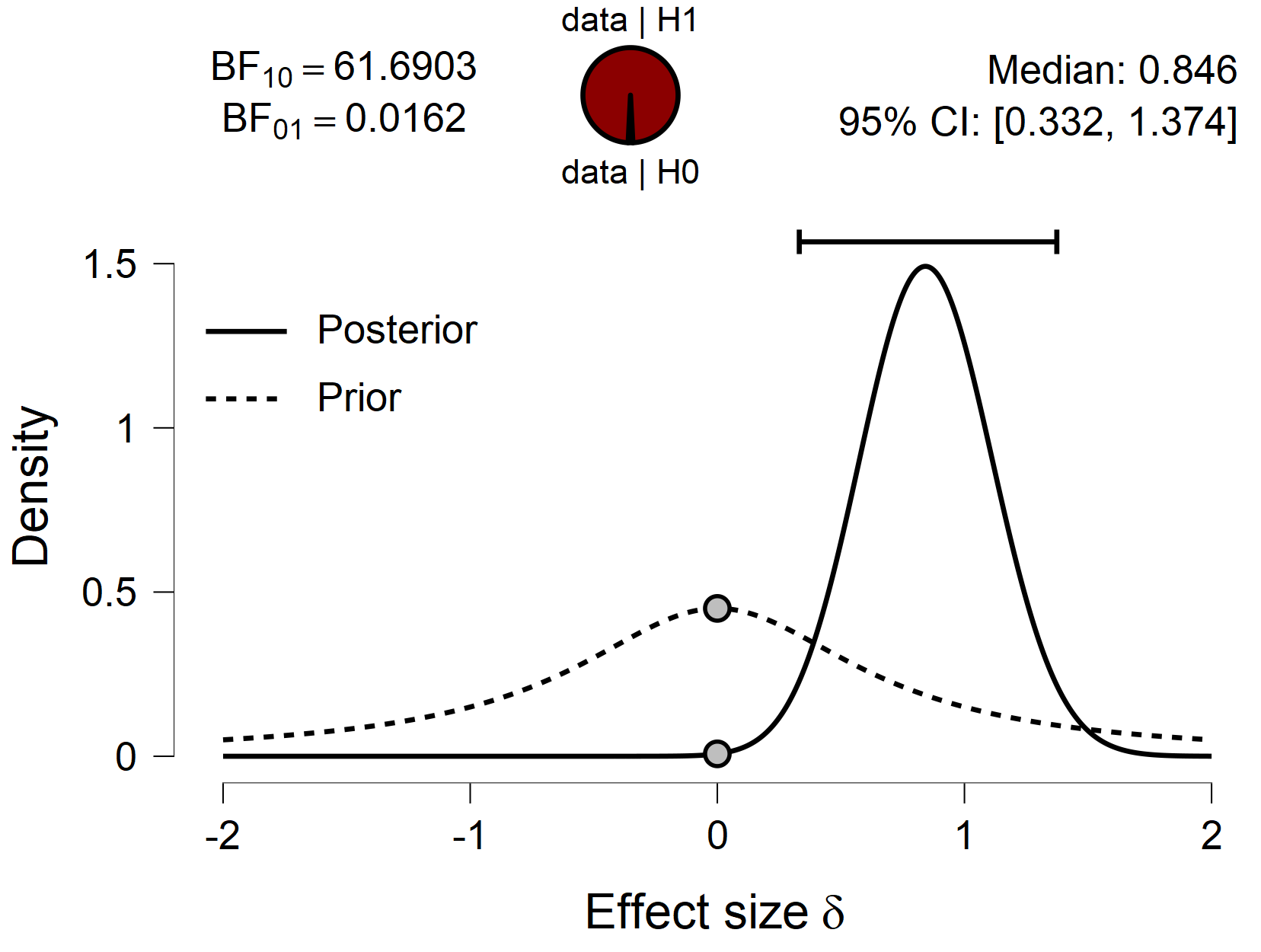


##### Bayes Factor Robustness Check


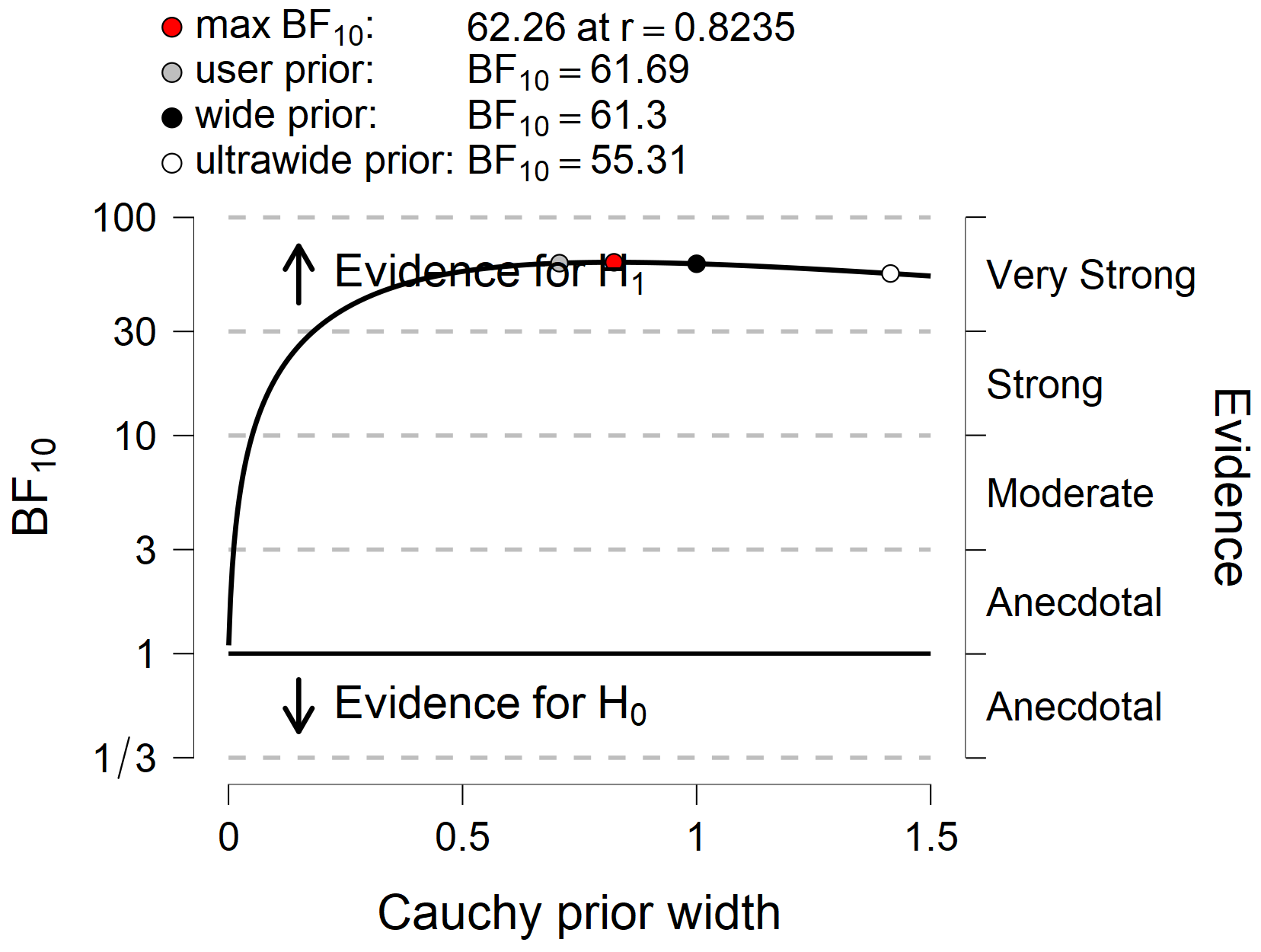


#### *Aperture Width*

##### Prior and Posterior


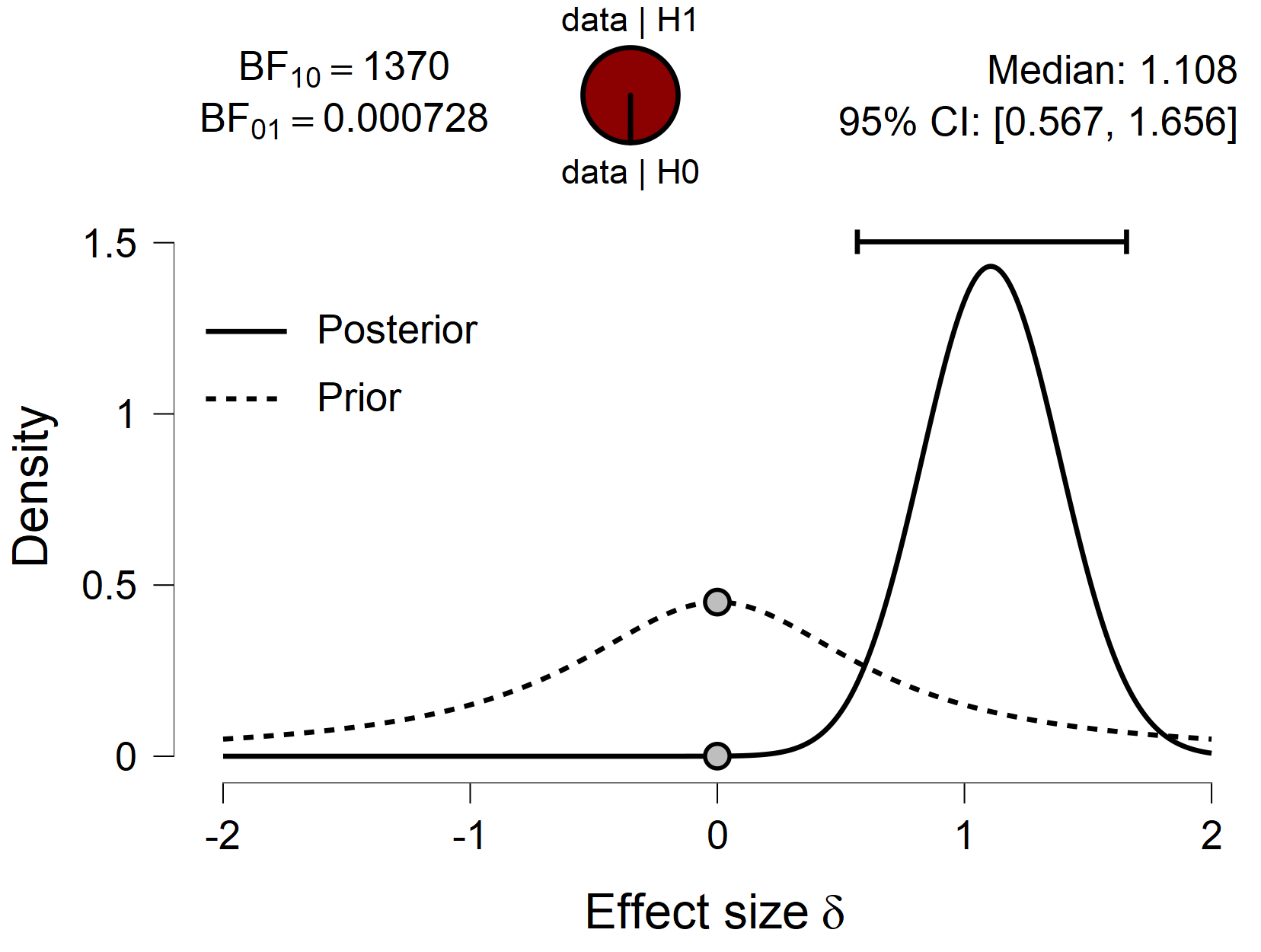


##### Bayes Factor Robustness Check


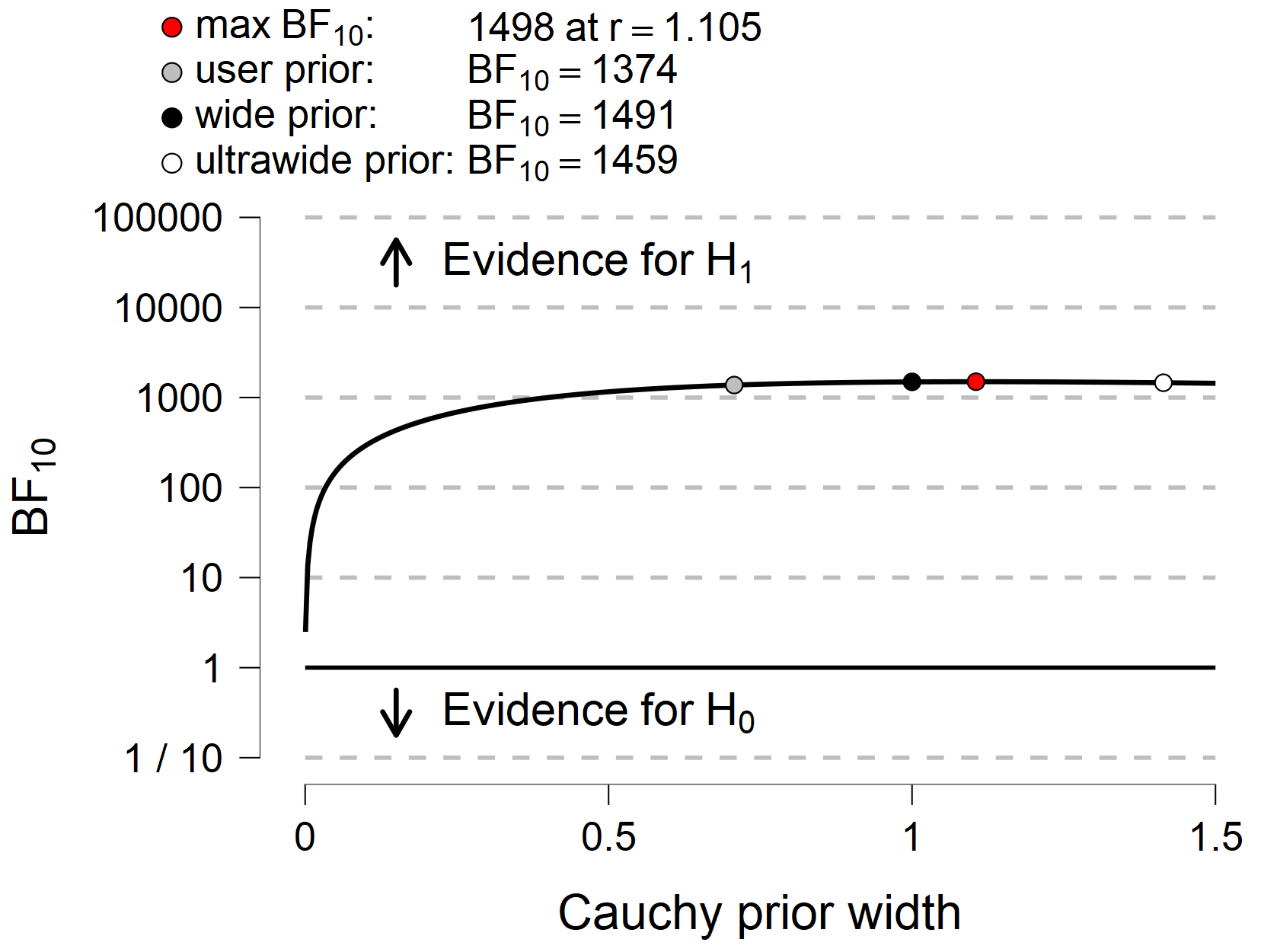


#### *Spire Height*

##### Prior and Posterior


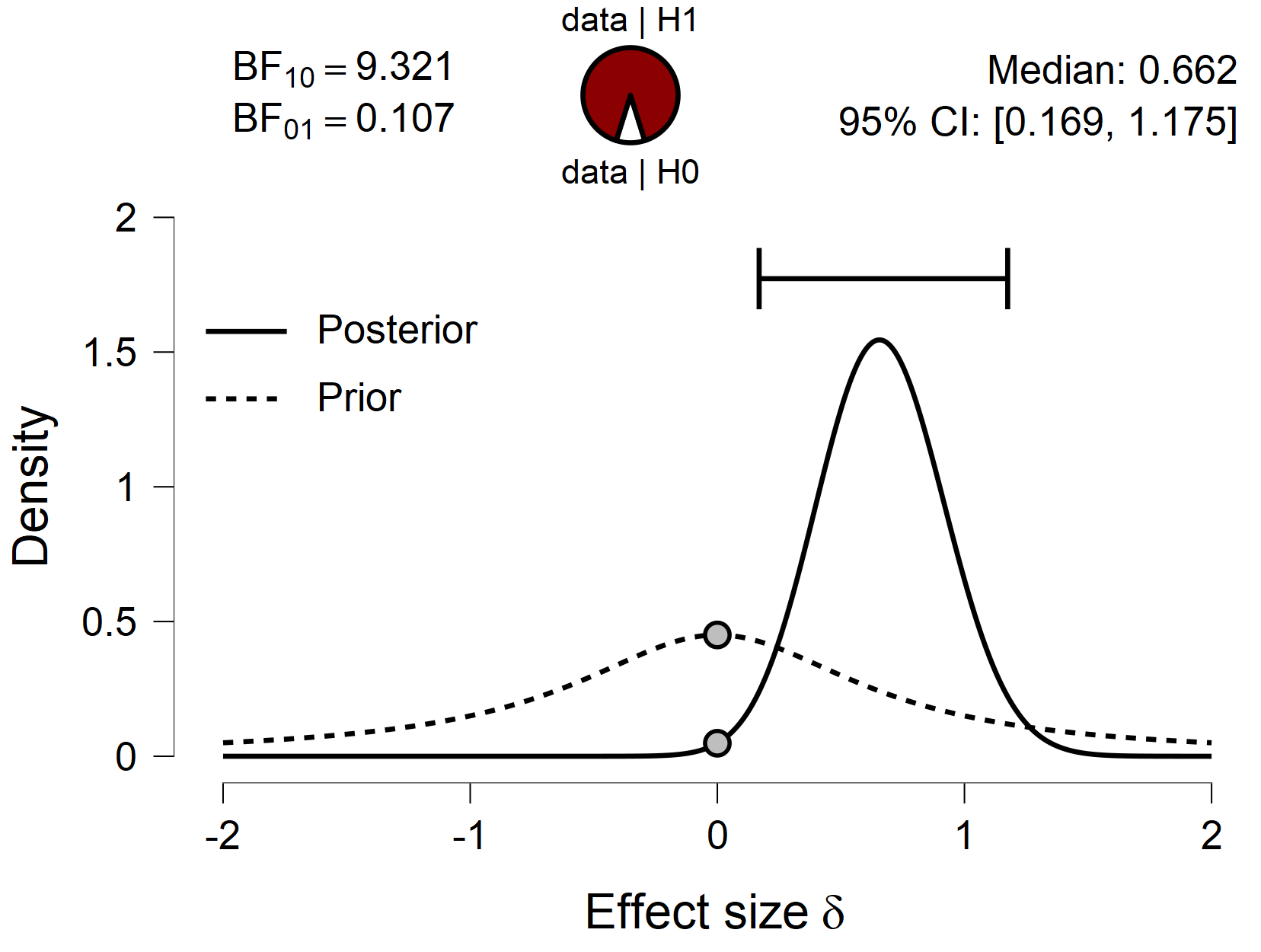


##### Bayes Factor Robustness Check


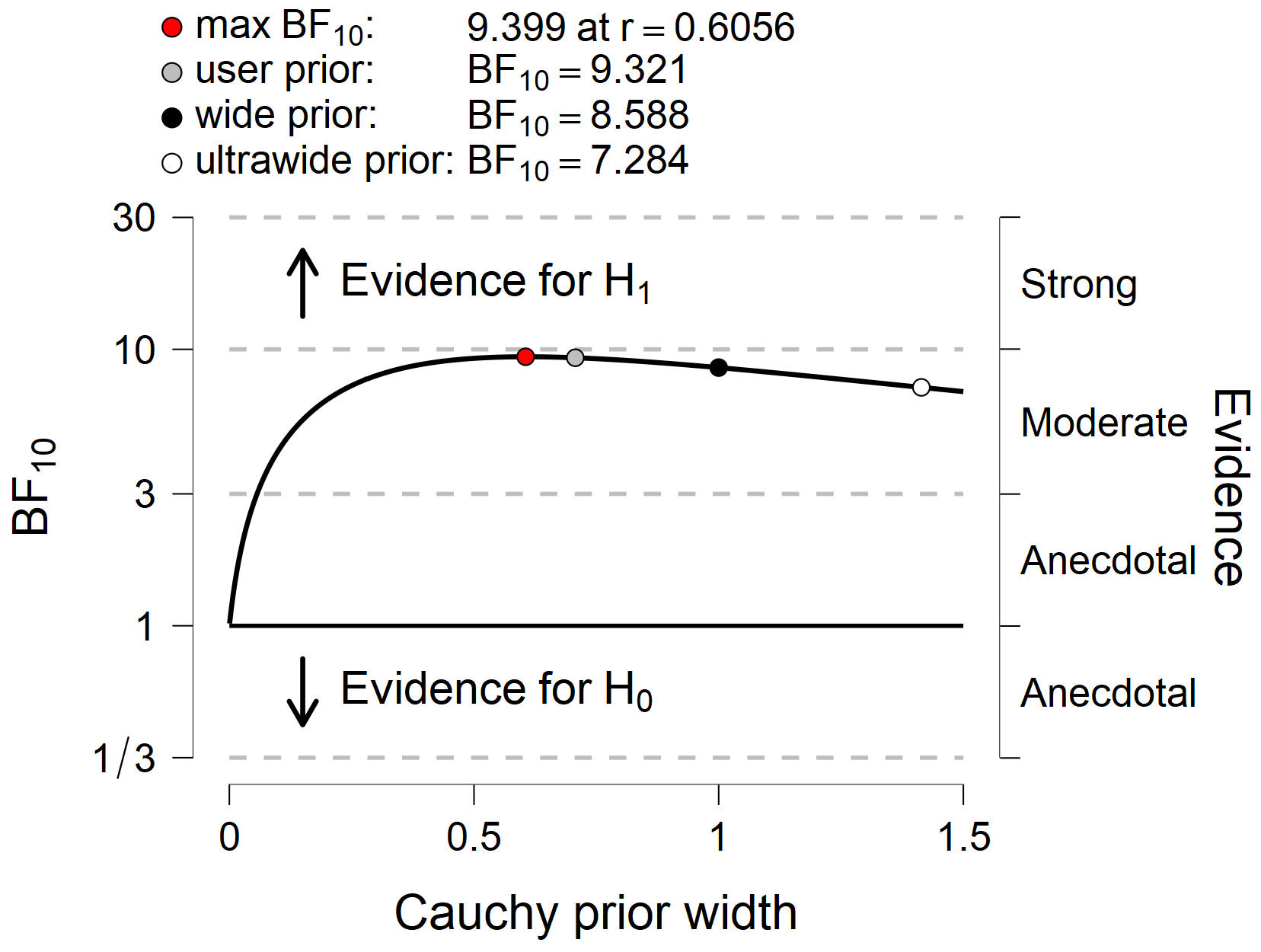


| **Descriptives** | | | | | | | | | | | | | | | | | |
| --- | --- | --- | --- | --- | --- | --- | --- | --- | --- | --- | --- | --- | --- | --- | --- | --- | --- |
|  | | | | | | | | | | | | | | **95% Credible Interval** | | | |
|  | | **Group** | | **N** | | **Mean** | | | **SD** | | | **SE** | | **Lower** | | **Upper** | |
| Shell Height |  | female |  | 33 |  | | 13.843 |  | | 0.876 |  | 0.152 |  | 13.532 |  | 14.153 |  |
|  |  | male |  | 30 |  | | 12.971 |  | | 0.878 |  | 0.160 |  | 12.644 |  | 13.299 |  |
| Shell Width |  | female |  | 33 |  | | 13.460 |  | | 0.708 |  | 0.123 |  | 13.209 |  | 13.712 |  |
|  |  | male |  | 30 |  | | 12.566 |  | | 0.780 |  | 0.142 |  | 12.274 |  | 12.857 |  |
| Aperture Height |  | female |  | 33 |  | | 7.378 |  | | 0.412 |  | 0.072 |  | 7.232 |  | 7.524 |  |
|  |  | male |  | 30 |  | | 6.972 |  | | 0.456 |  | 0.083 |  | 6.802 |  | 7.142 |  |
| Aperture Width |  | female |  | 33 |  | | 8.169 |  | | 0.457 |  | 0.080 |  | 8.007 |  | 8.331 |  |
|  |  | male |  | 30 |  | | 7.637 |  | | 0.429 |  | 0.078 |  | 7.477 |  | 7.797 |  |
| Spire Height |  | female |  | 33 |  | | 3.436 |  | | 0.362 |  | 0.063 |  | 3.308 |  | 3.565 |  |
|  |  | male |  | 30 |  | | 3.191 |  | | 0.285 |  | 0.052 |  | 3.085 |  | 3.297 |  |
|  | | | | | | | | | | | | | | | | | |

**Table S3.** Eigenvalues for principal component analysis (PCA) for shell shape of Leptopoma perlucidum

|  | **Comp1** | **Comp2** | **Comp3** | **Comp4** | **Comp5** | **Comp6** | **Comp7** |
| --- | --- | --- | --- | --- | --- | --- | --- |
| **Eigenvalues** | 0.00041 | 0.00030 | 0.00021 | 0.00009 | 0.00007 | 0.00005 | 0.00004 |
| **Proportion of Variance (%)** | 32% | 23% | 16% | 7% | 5% | 4% | 3% |
| **Cumulative Variance (%)** | 32% | 55% | 71% | 78% | 83% | 87% | 91% |

**Table S4.** Procrustes ANOVA (regression for shape data) on the sources of shape variation using the symmetric component of shape. The R-squared column (Rsq) of this Procrustes ANOVA demonstrates the relative contribution of each factor to overall variation. Procrustes ANOVA testing for shape differences between opposites sexes, locations, and interactions between them.

|  | **df** | **SS** | **MS** | **Rsq** | **F** | **Z** | **p** |
| --- | --- | --- | --- | --- | --- | --- | --- |
| **Sexes** | 1 | 0.001951 | 0.001951 | 0.01823 | 1.5247 | 1.0329 | 0.150 |
| **Locations** | 1 | 0.002164 | 0.002164 | 0.02021 | 1.6907 | 1.1886 | 0.117 |
| **Sexes:Locations** | 1 | 0.000558 | 0.000558 | 0.00521 | 0.4359 | -1.1648 | 0.879 |
| **Residuals** | 80 | 0.102376 | 0.00128 | 0.95635 |  |  |  |
| **Total** | 83 | 0.107049 |  |  |  |  |  |
