# Supplementary figures and images for: Sexual dimorphism in shell size of the land snail Leptopoma perlucidum (Caenogastropoda: Cyclophoridae)
Source: PeerJ. 2022 May 27;10:e13501. doi: 10.7717/peerj.13501 (PMC9150688; doi:10.7717/peerj.13501)

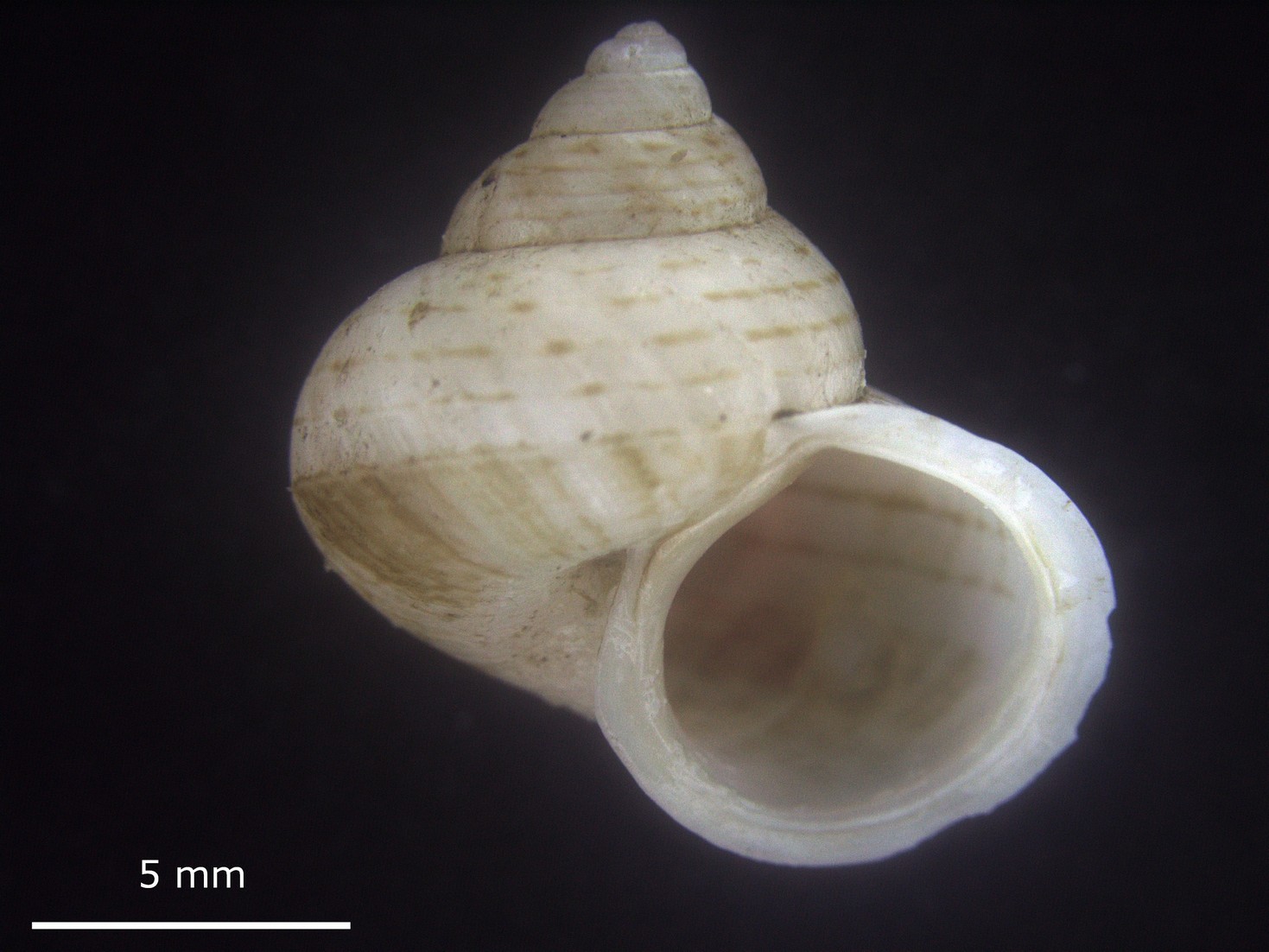

Supplement: File S4 [file peerj-10-13501-s004.zip › New Folder/6651.1.jpg]

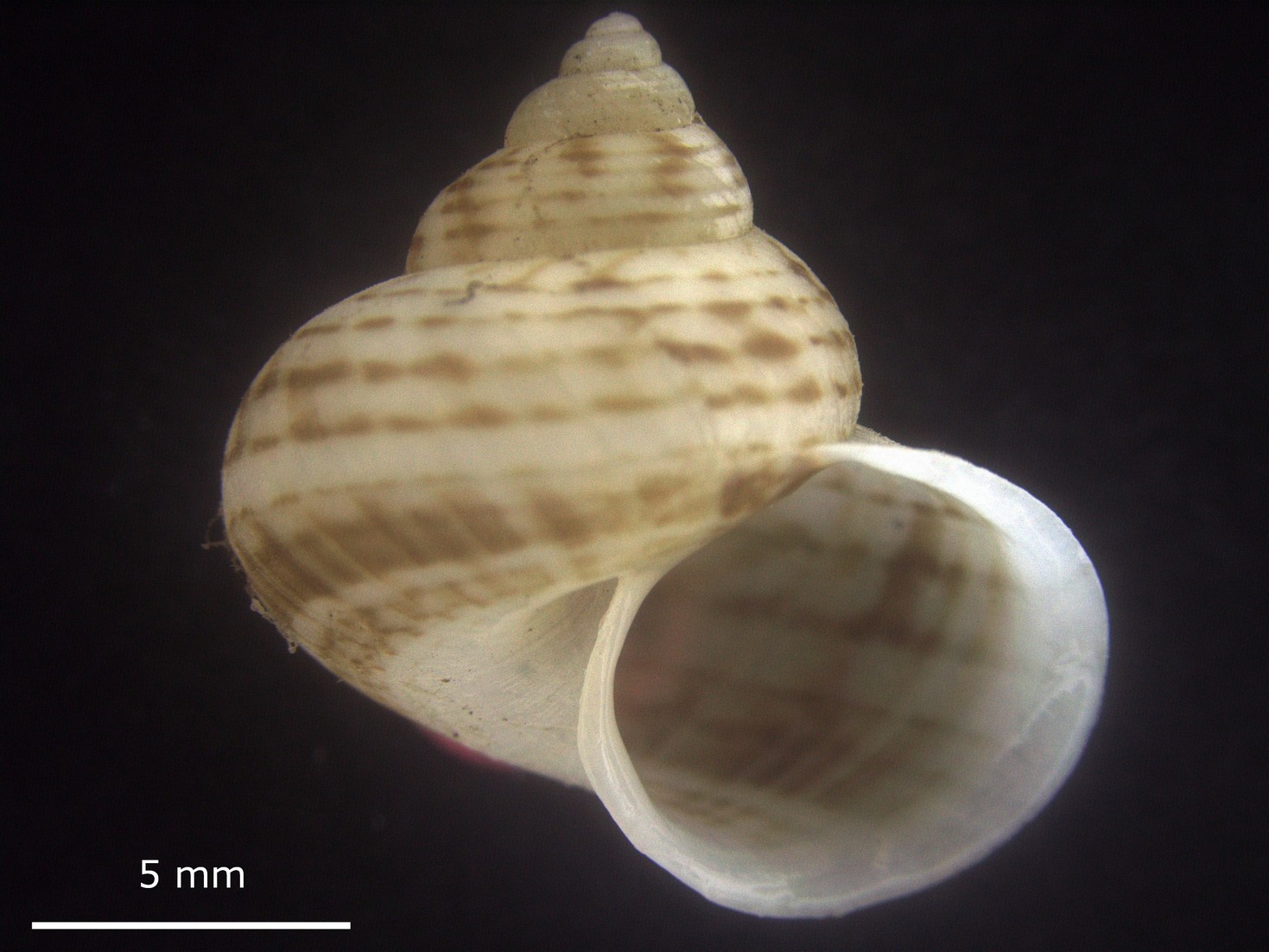

Supplement: File S4 [file peerj-10-13501-s004.zip › New Folder/6653.1.jpg]

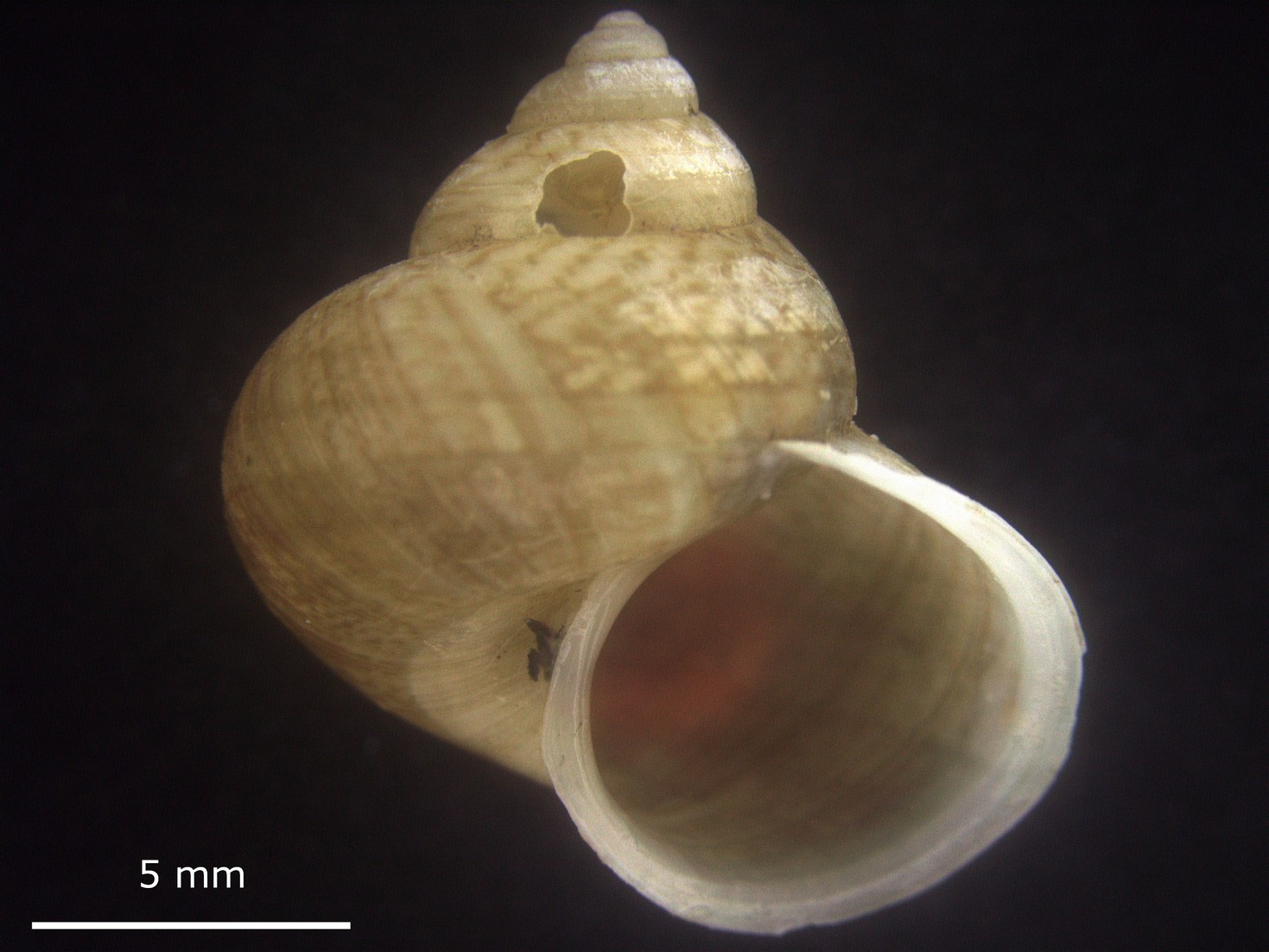

Supplement: File S4 [file peerj-10-13501-s004.zip › New Folder/6654.1.jpg]

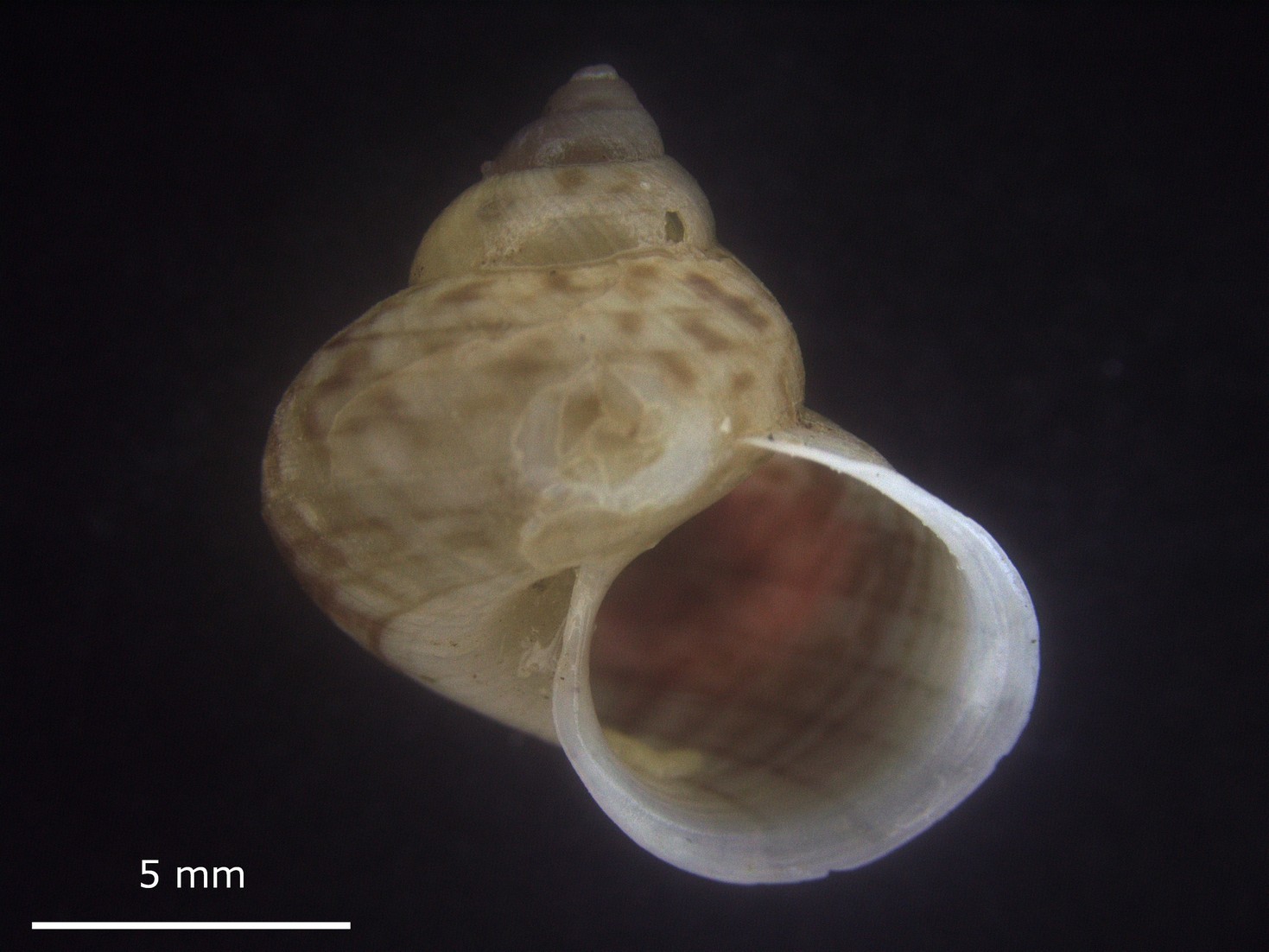

Supplement: File S4 [file peerj-10-13501-s004.zip › New Folder/7873.1.jpg]

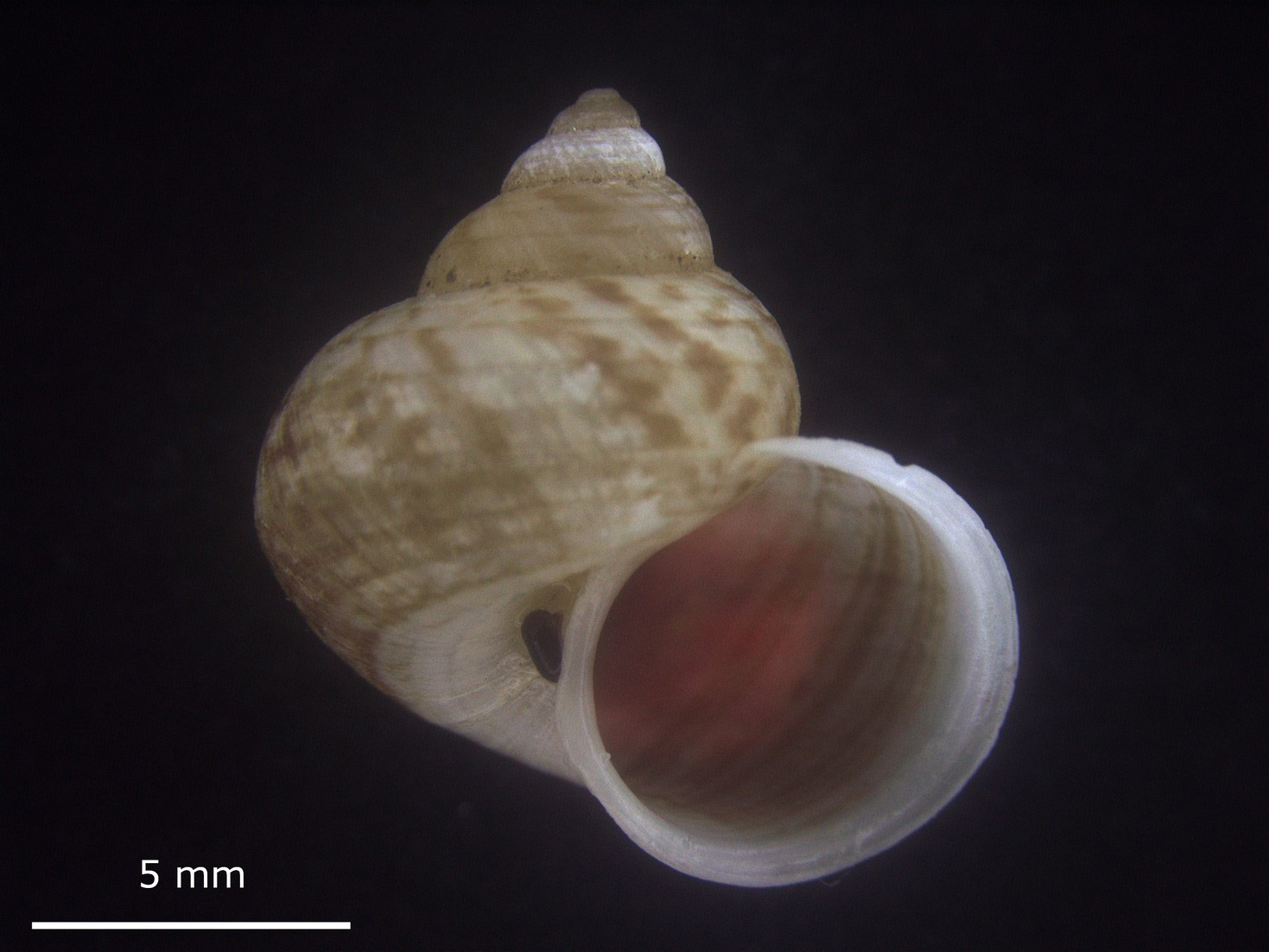

Supplement: File S4 [file peerj-10-13501-s004.zip › New Folder/7874.1.jpg]

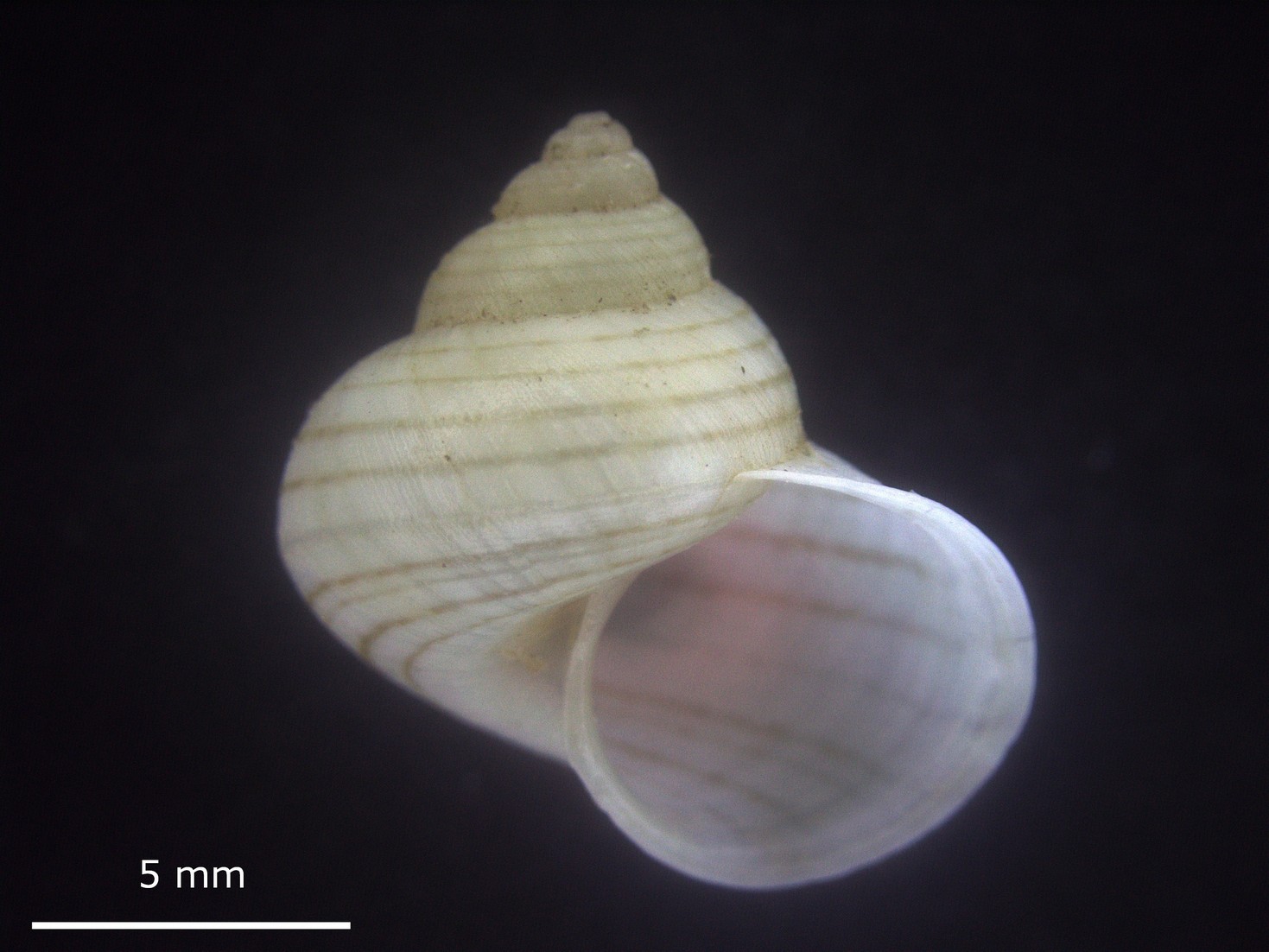

Supplement: File S4 [file peerj-10-13501-s004.zip › New Folder/7875.1.jpg]

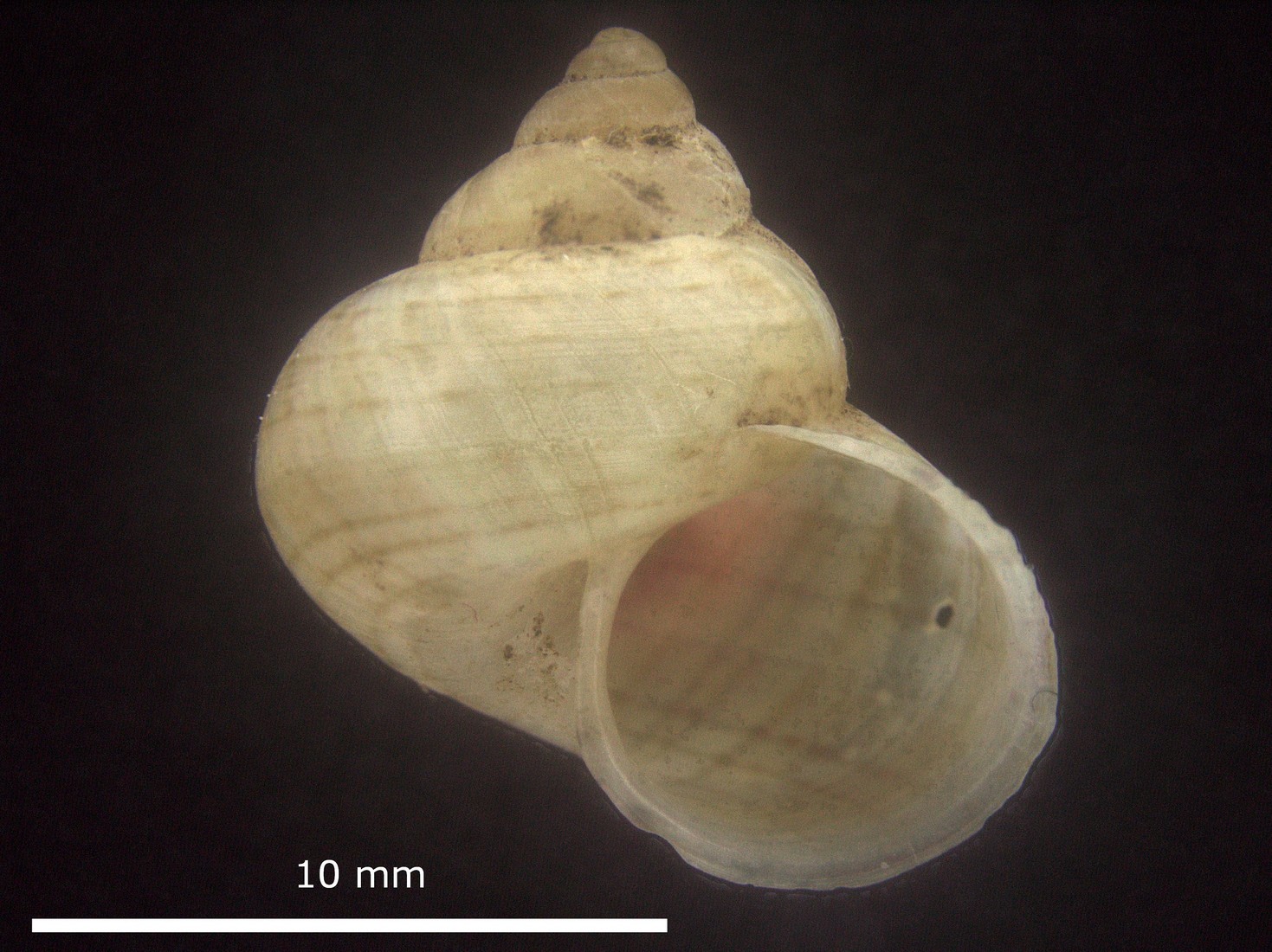

Supplement: File S4 [file peerj-10-13501-s004.zip › New Folder/7877.1.jpg]

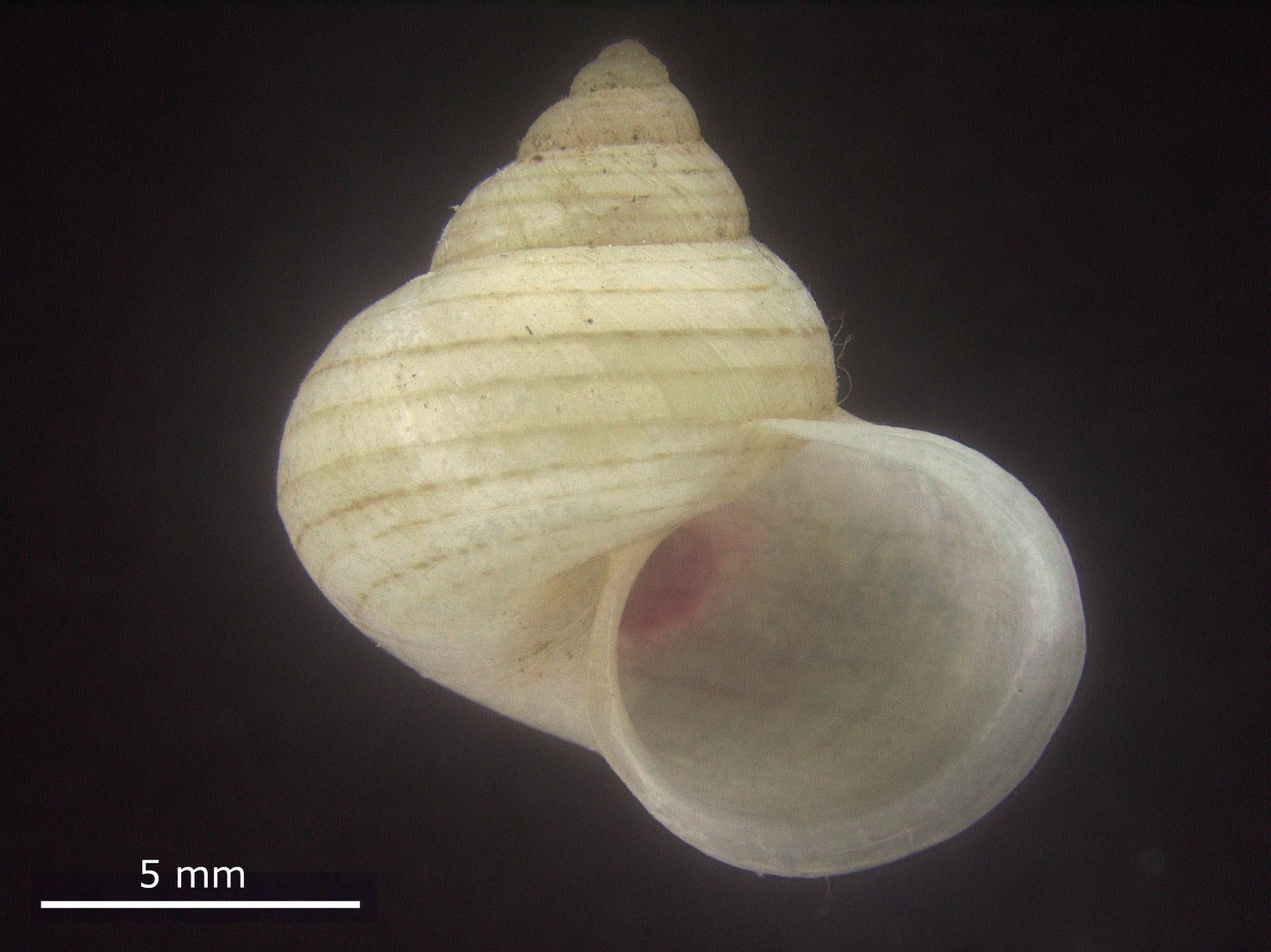

Supplement: File S4 [file peerj-10-13501-s004.zip › New Folder/8739.1.jpg]

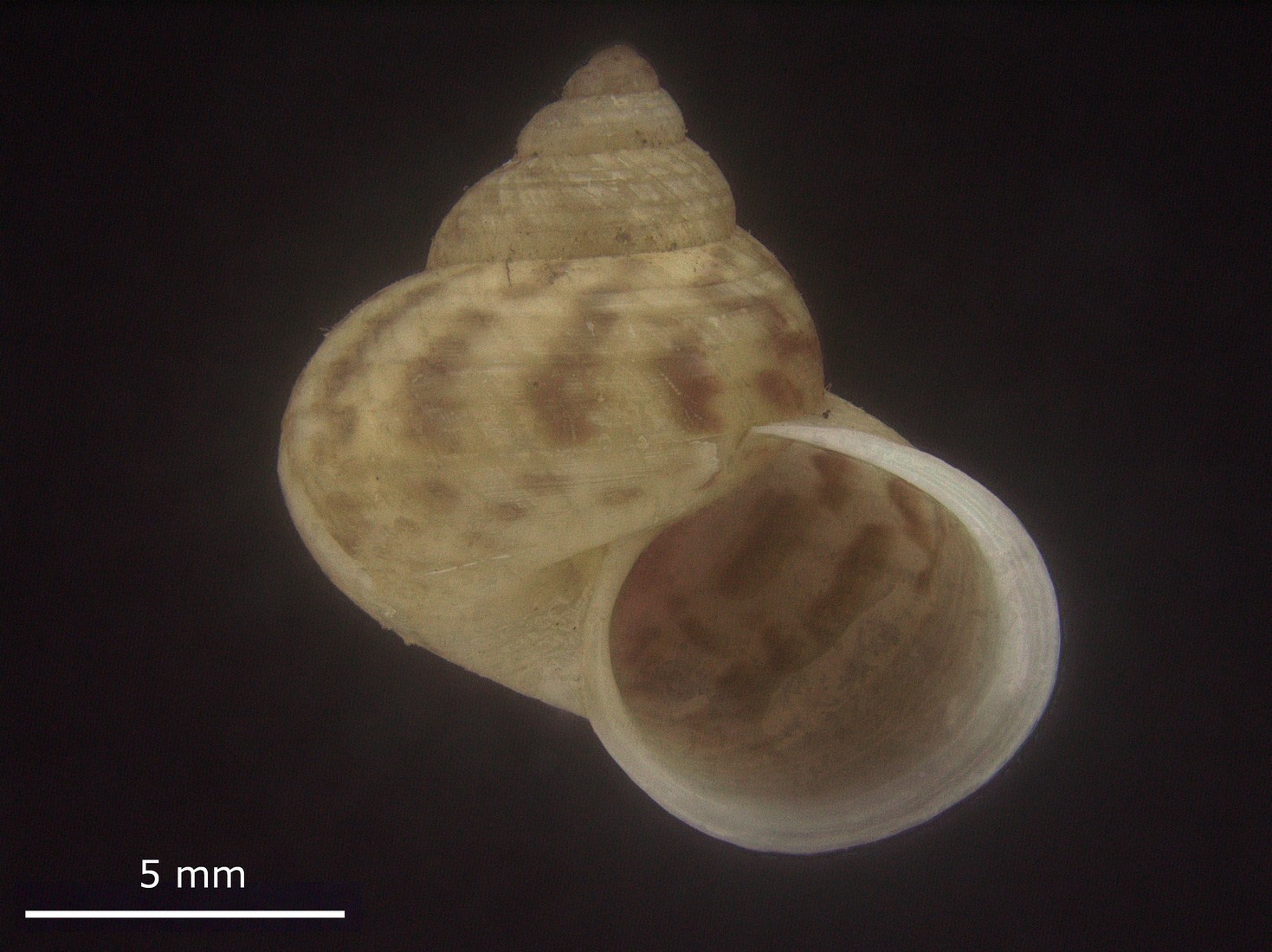

Supplement: File S4 [file peerj-10-13501-s004.zip › New Folder/8740.1.jpg]

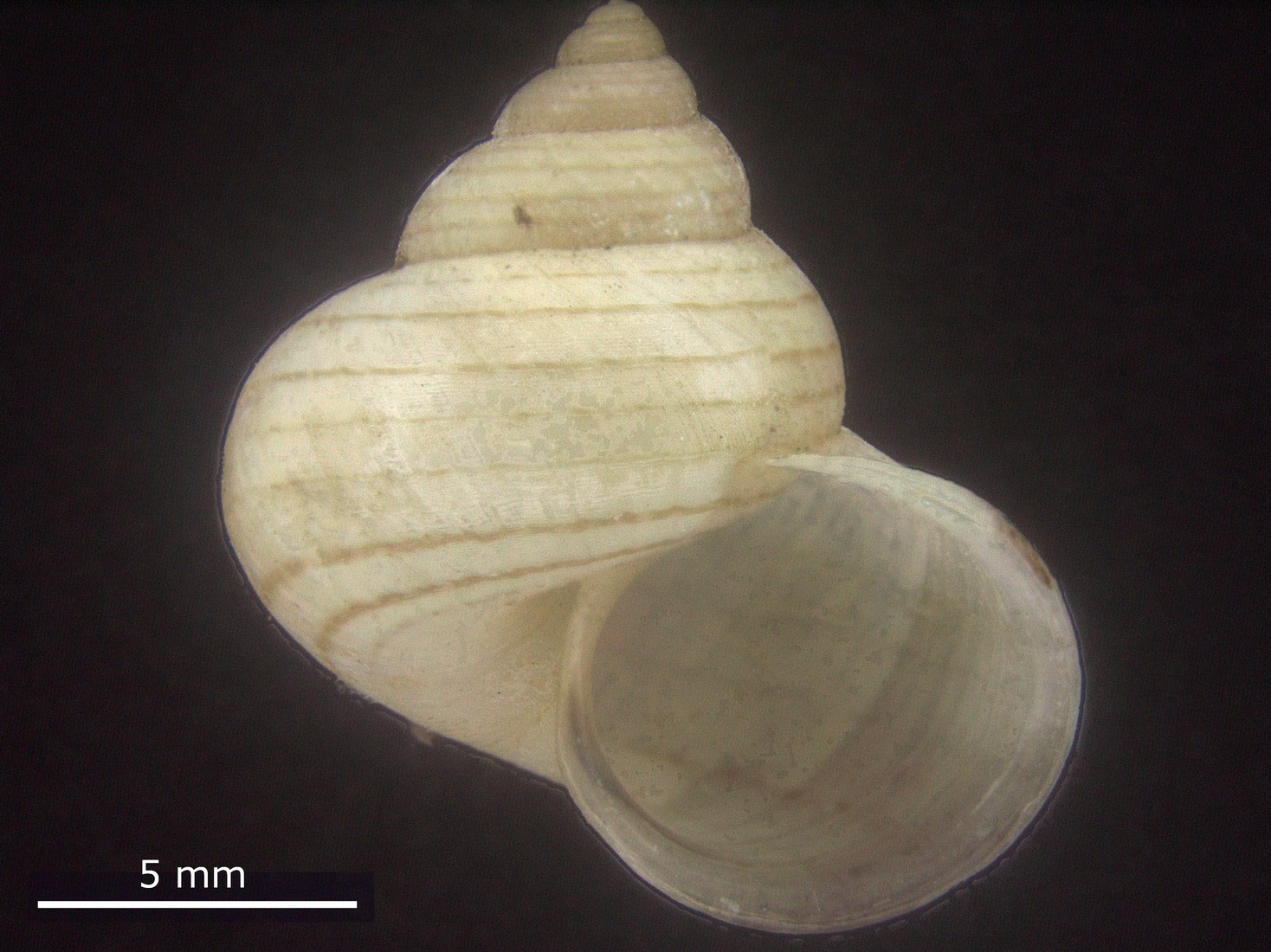

Supplement: File S4 [file peerj-10-13501-s004.zip › New Folder/8741.1.jpg]

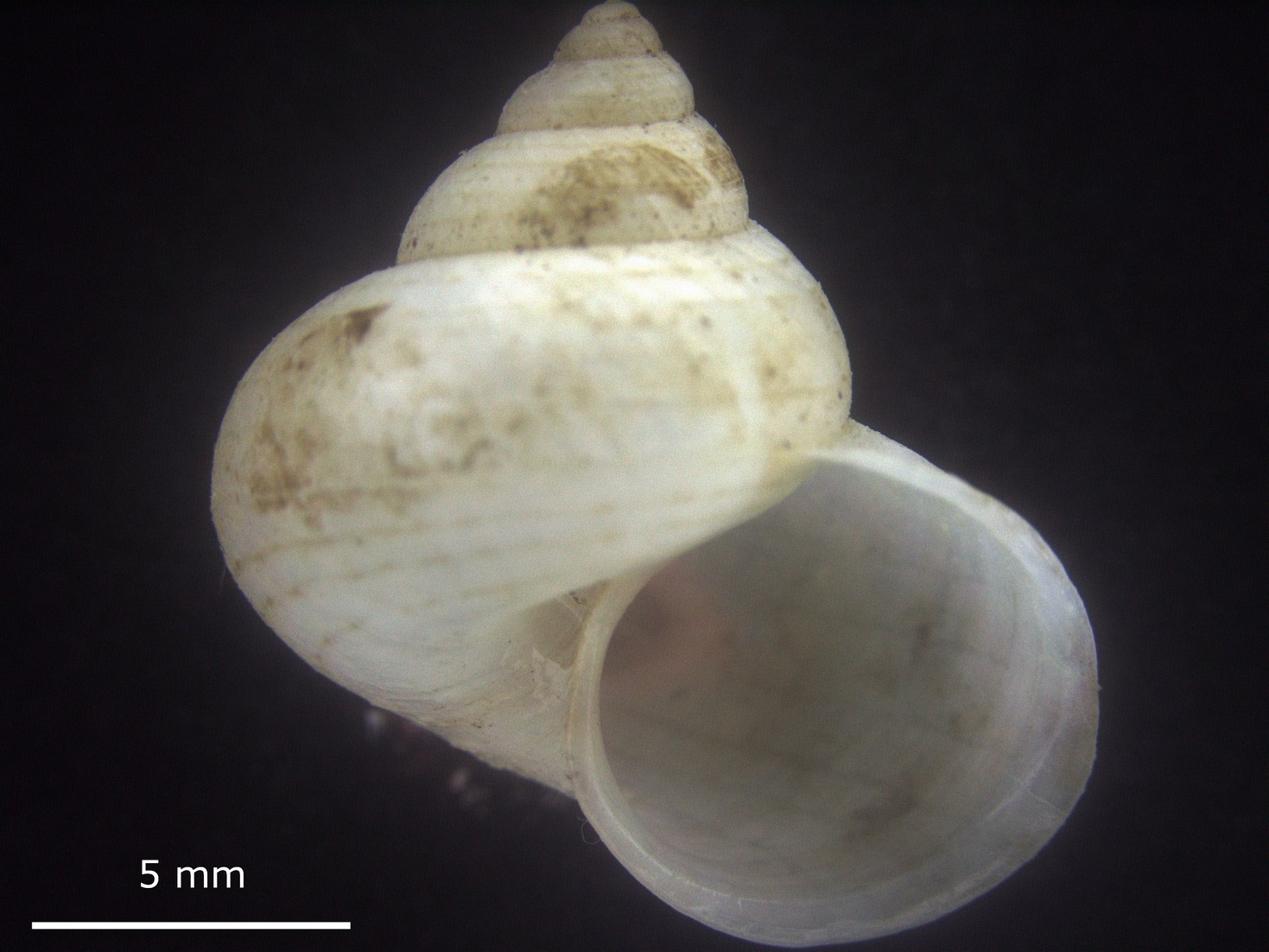

Supplement: File S4 [file peerj-10-13501-s004.zip › New Folder/8742.1.jpg]

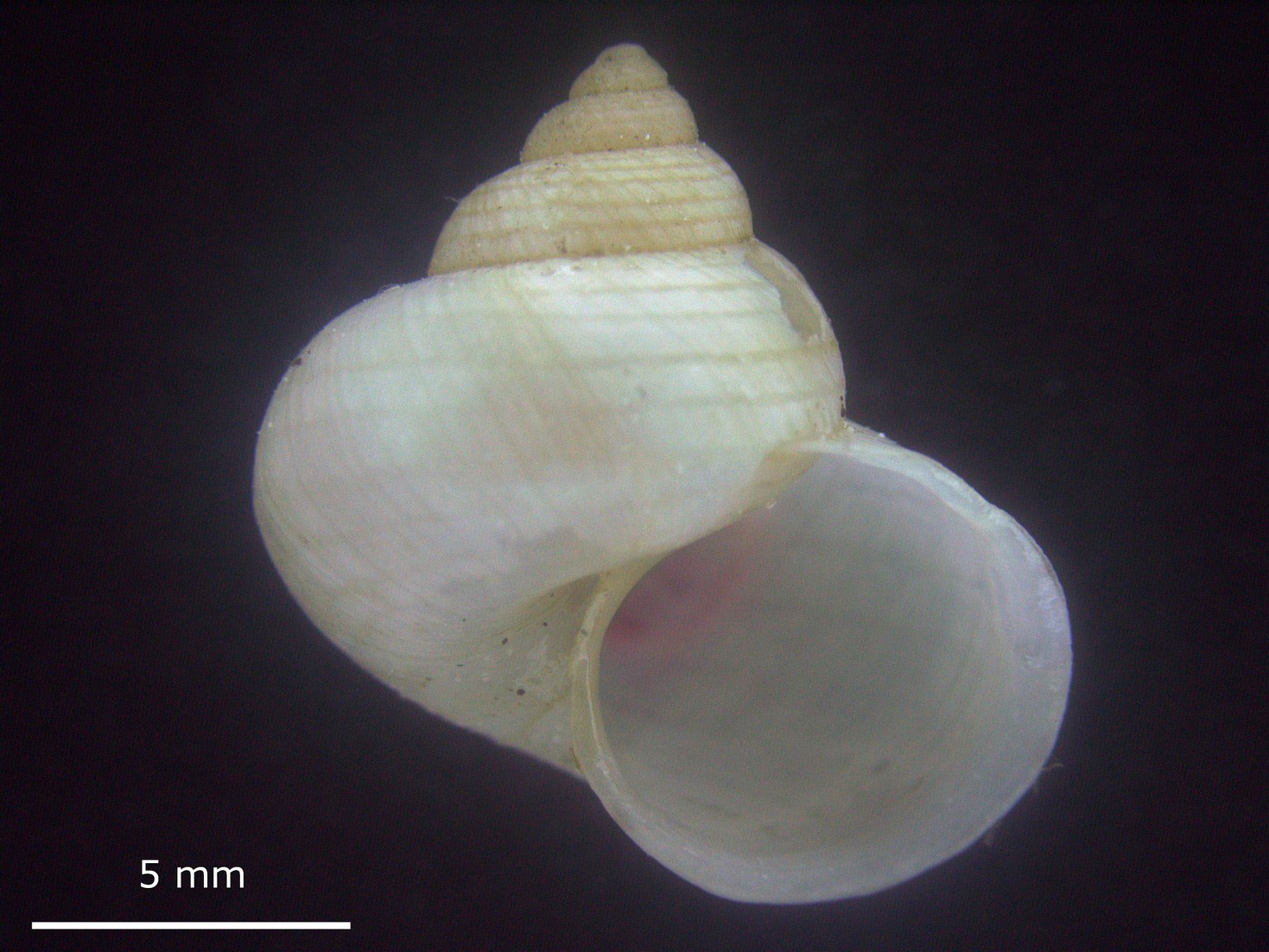

Supplement: File S4 [file peerj-10-13501-s004.zip › New Folder/8743.1.jpg]

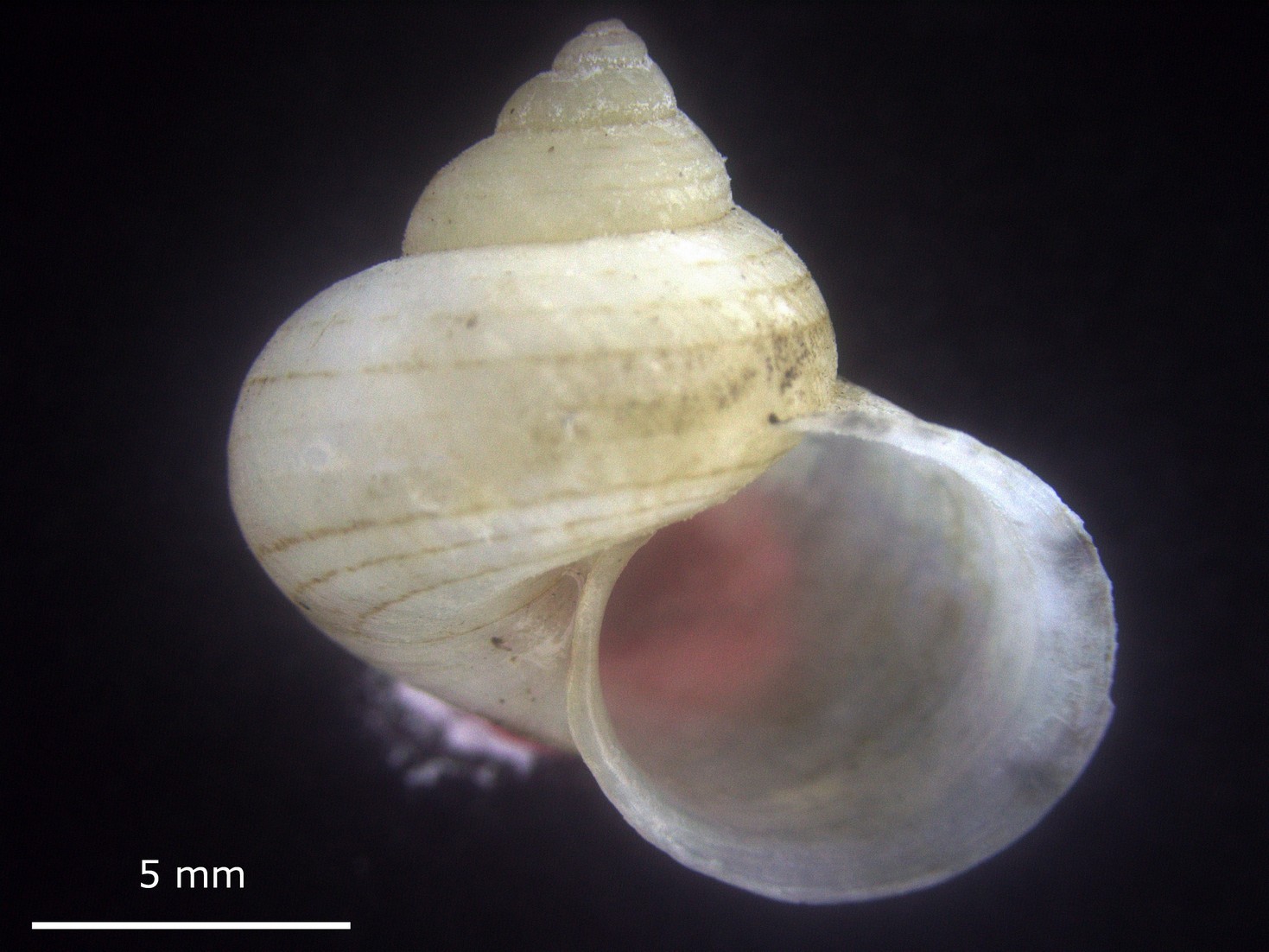

Supplement: File S4 [file peerj-10-13501-s004.zip › New Folder/8744.1.jpg]

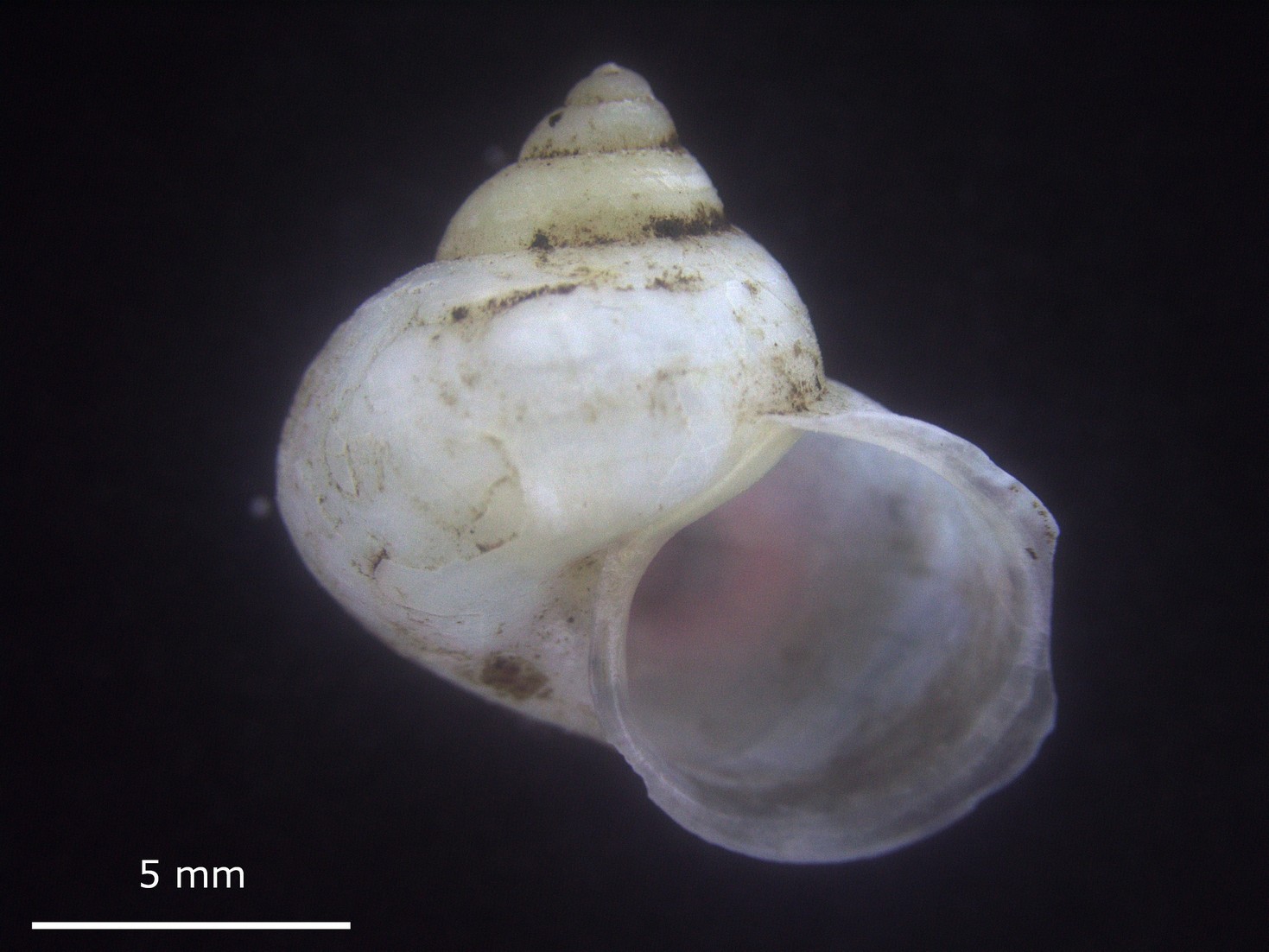

Supplement: File S4 [file peerj-10-13501-s004.zip › New Folder/8745.1.jpg]

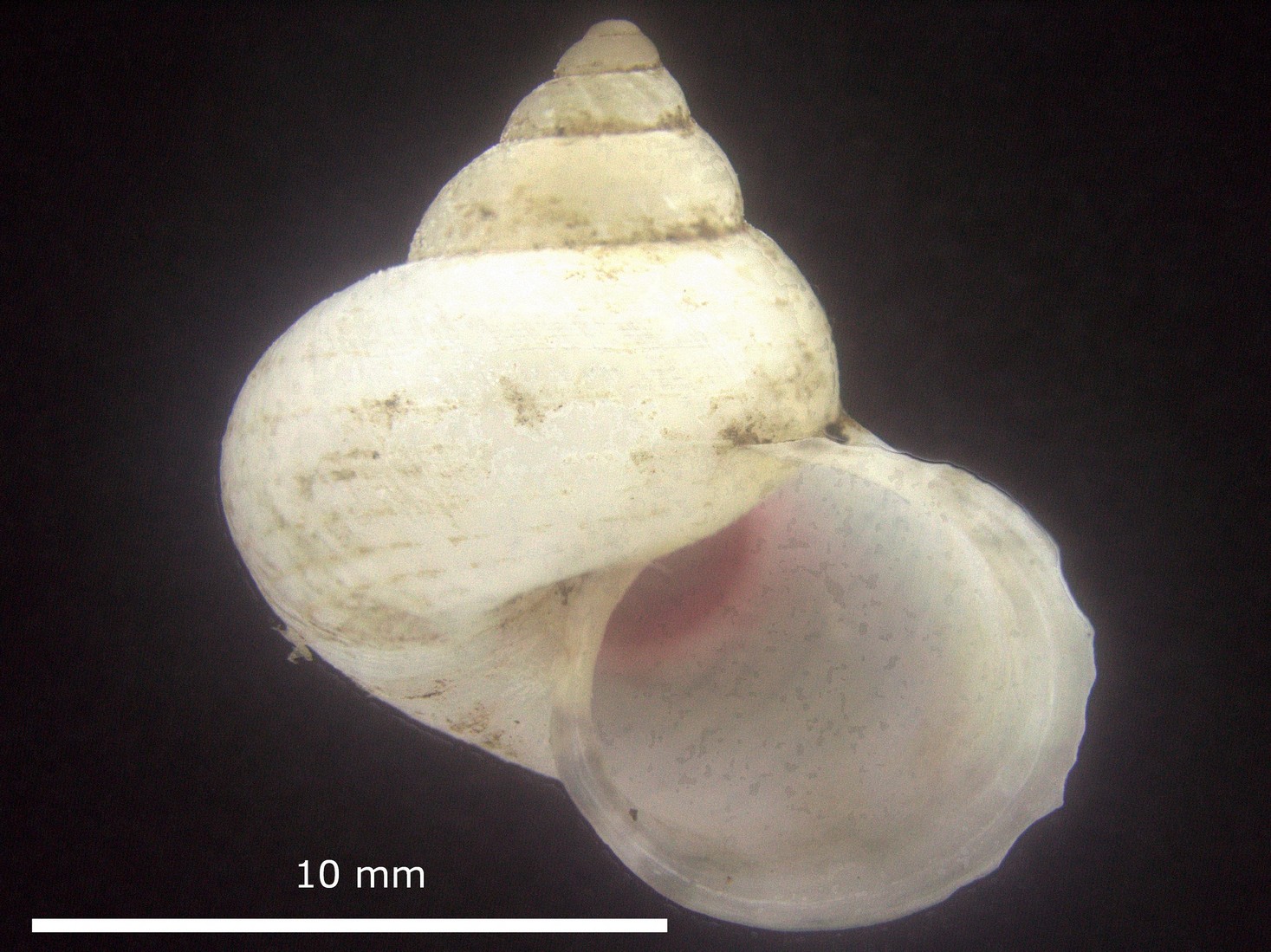

Supplement: File S4 [file peerj-10-13501-s004.zip › New Folder/8746.1.jpg]

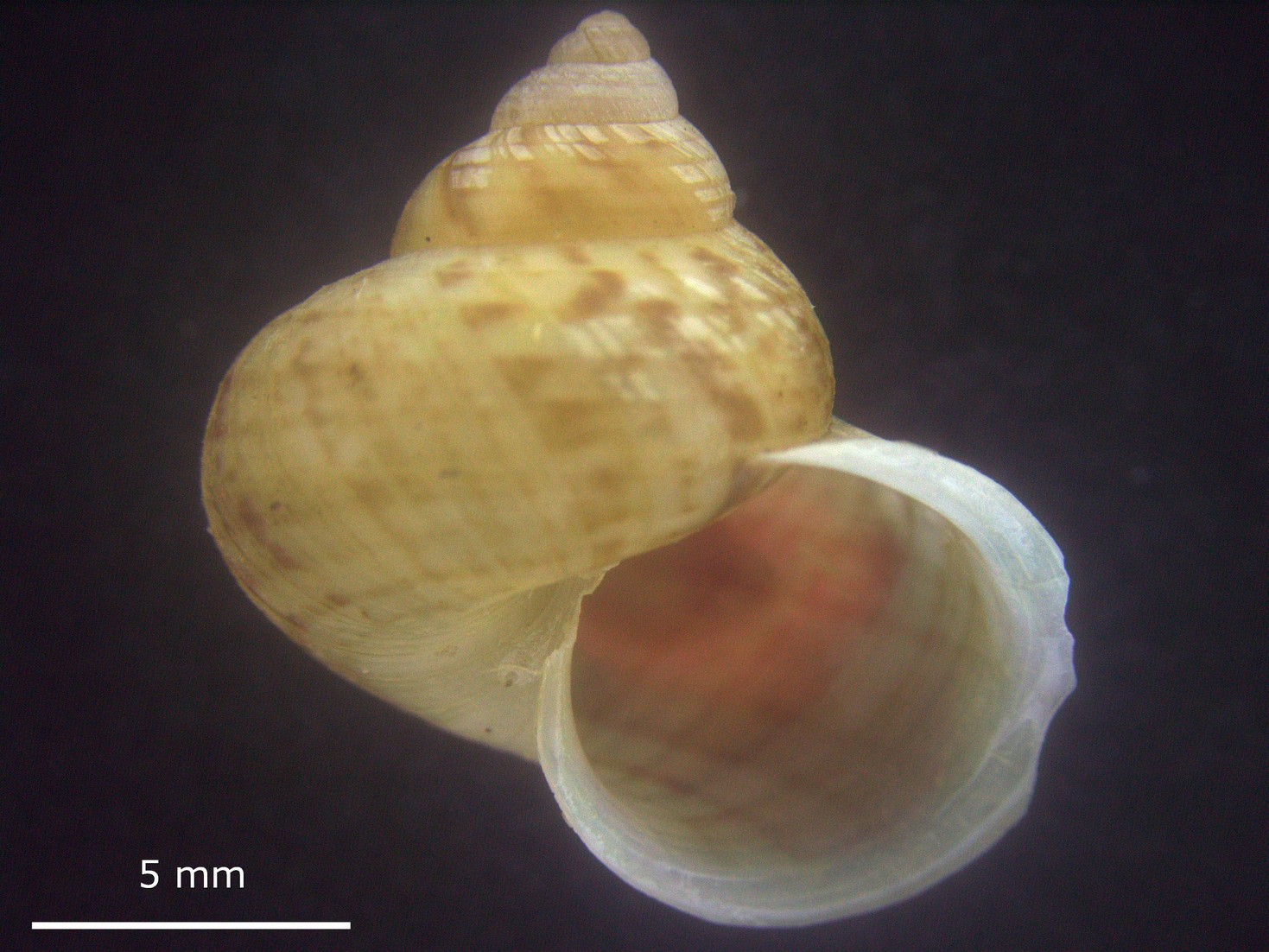

Supplement: File S4 [file peerj-10-13501-s004.zip › New Folder/8747.1.jpg]

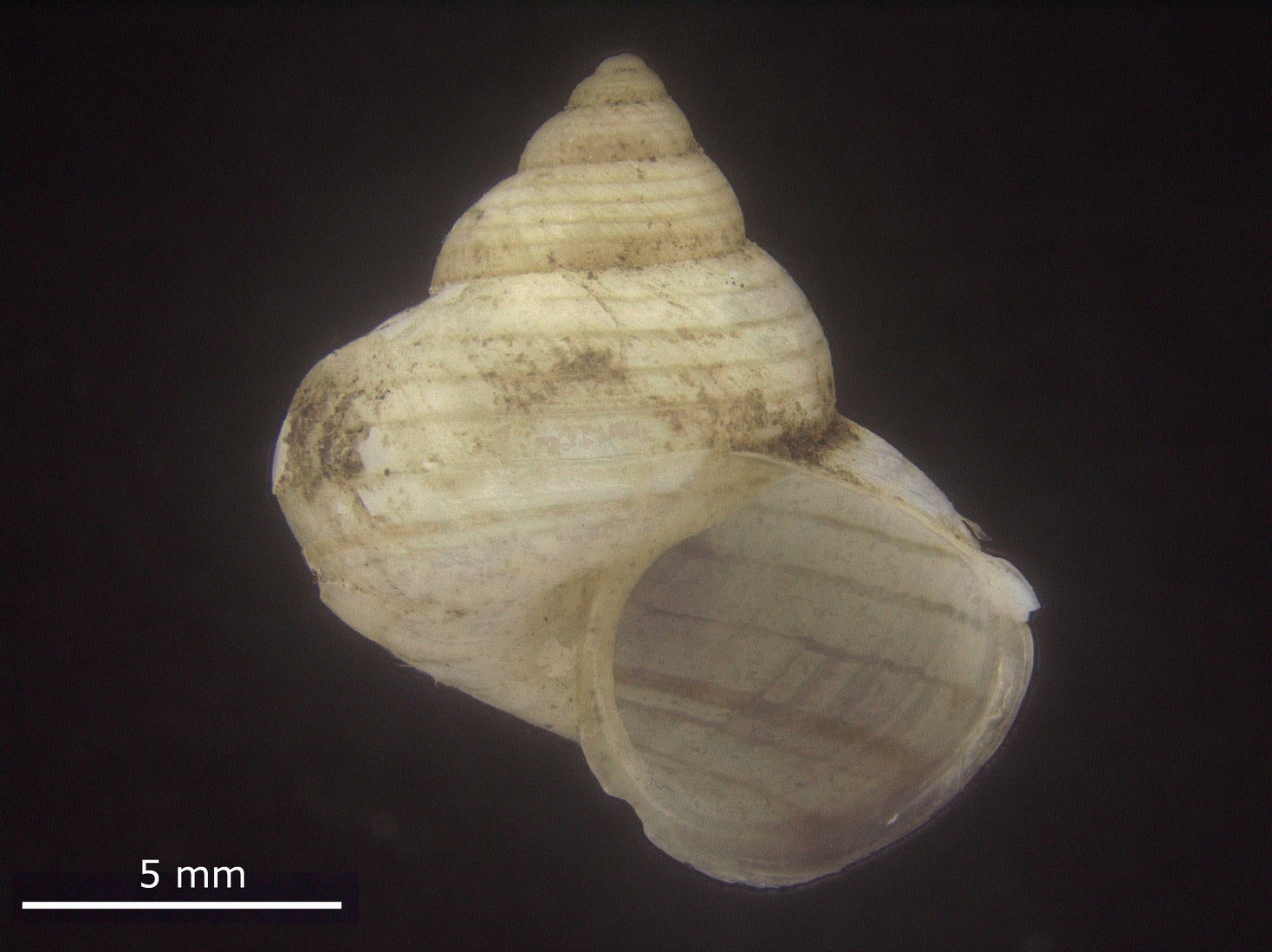

Supplement: File S4 [file peerj-10-13501-s004.zip › New Folder/8748.1.jpg]

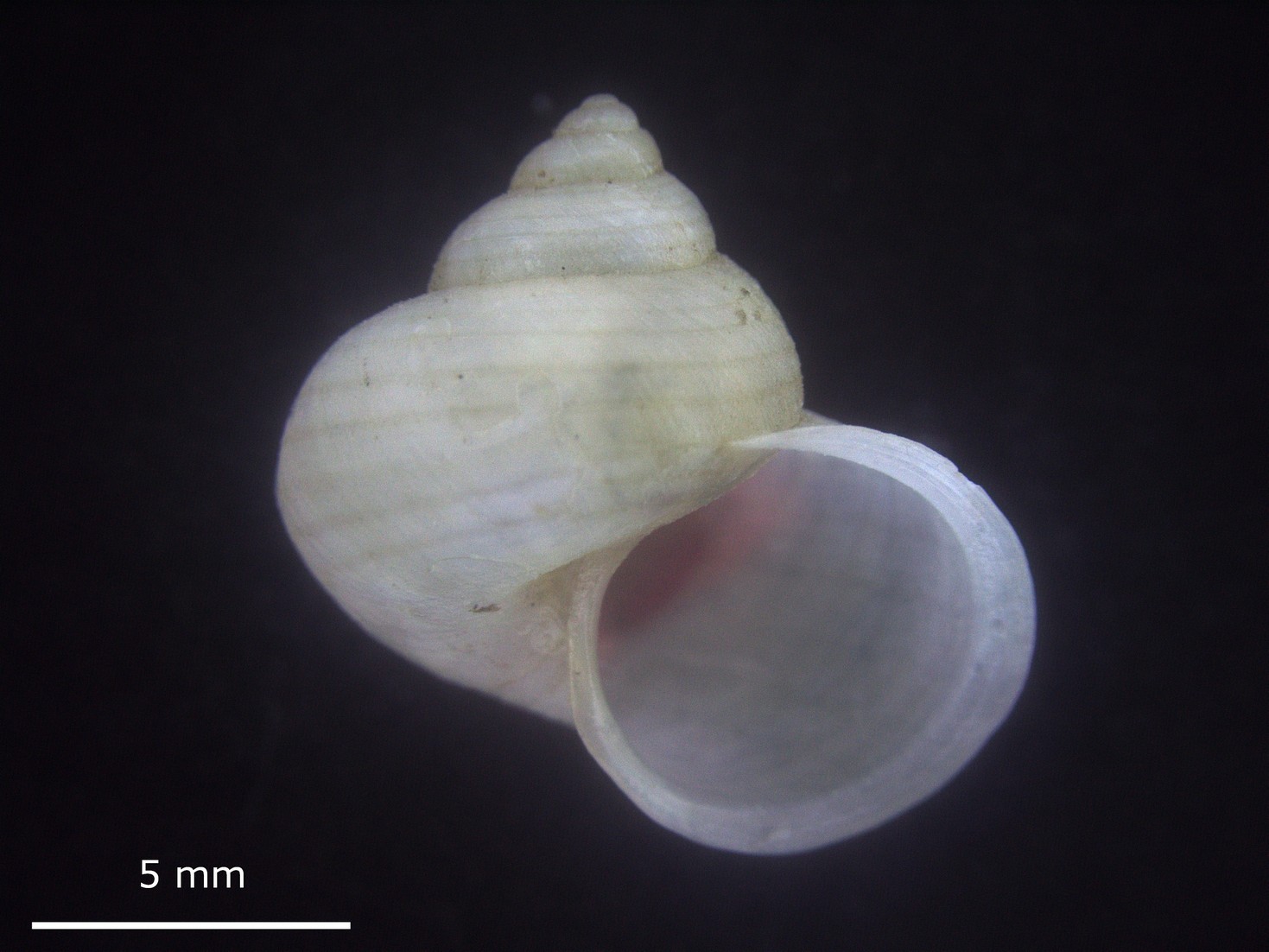

Supplement: File S4 [file peerj-10-13501-s004.zip › New Folder/8750.1.jpg]

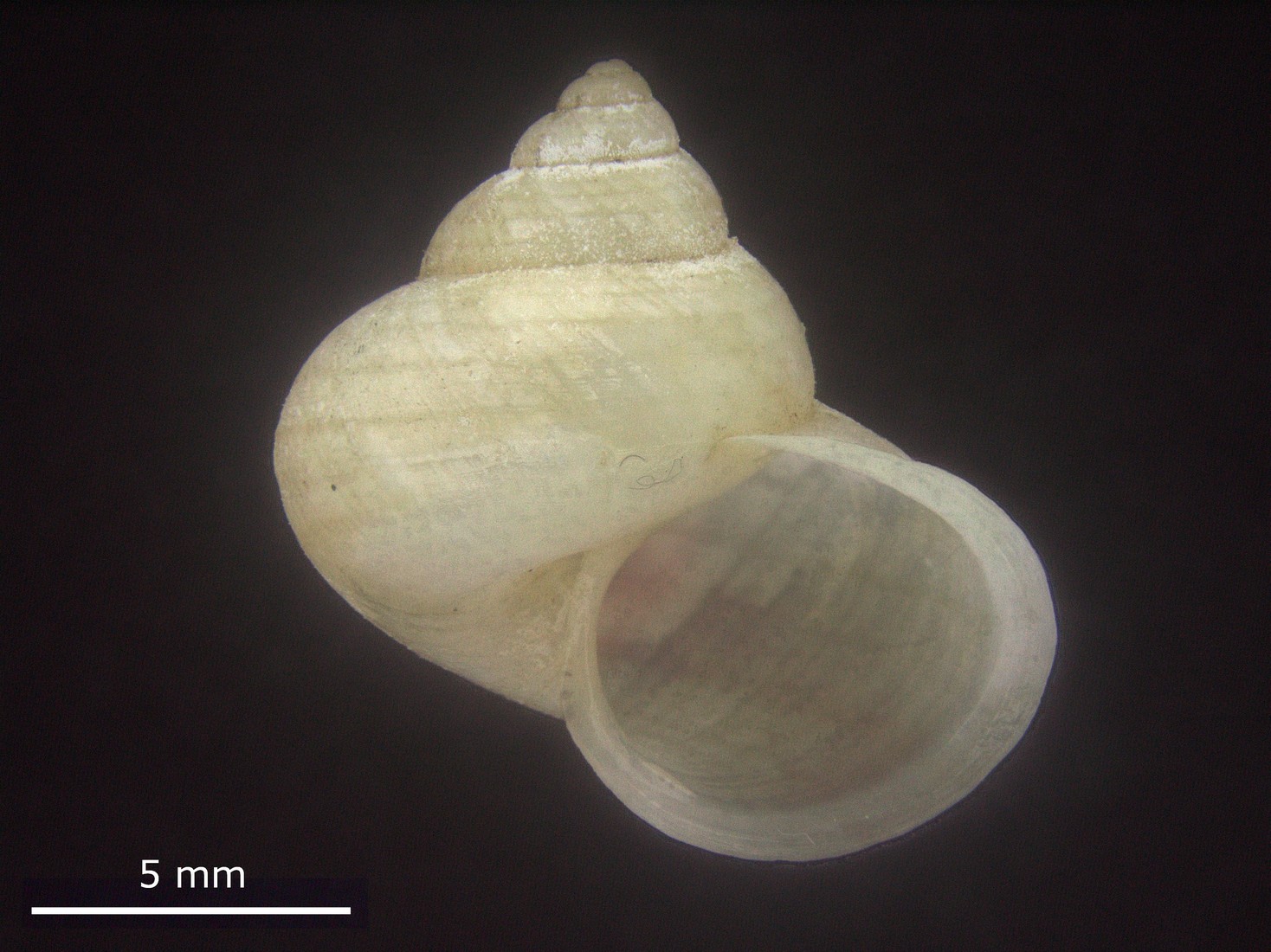

Supplement: File S4 [file peerj-10-13501-s004.zip › New Folder/8751.1.jpg]

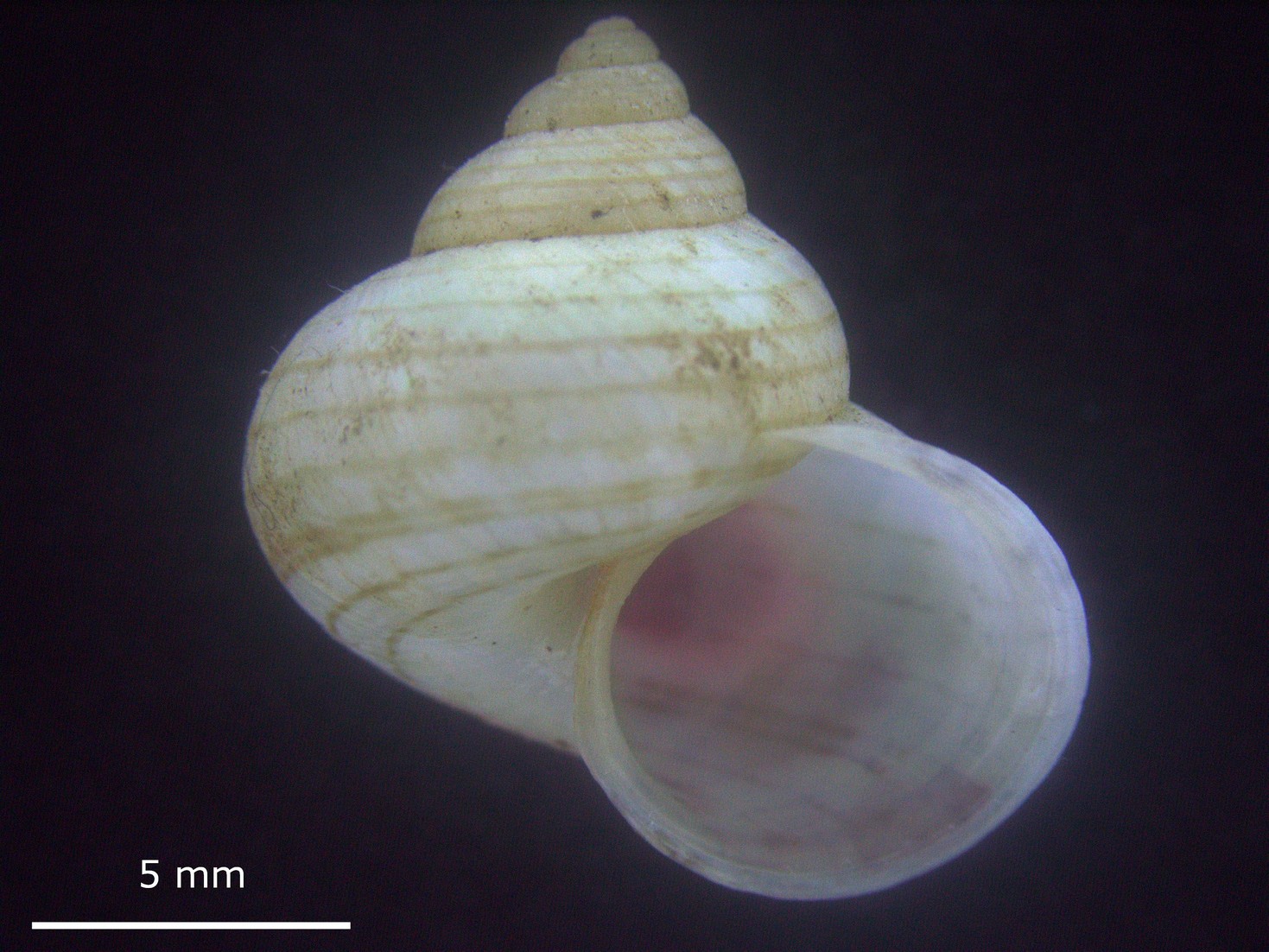

Supplement: File S4 [file peerj-10-13501-s004.zip › New Folder/8752.1..jpg]

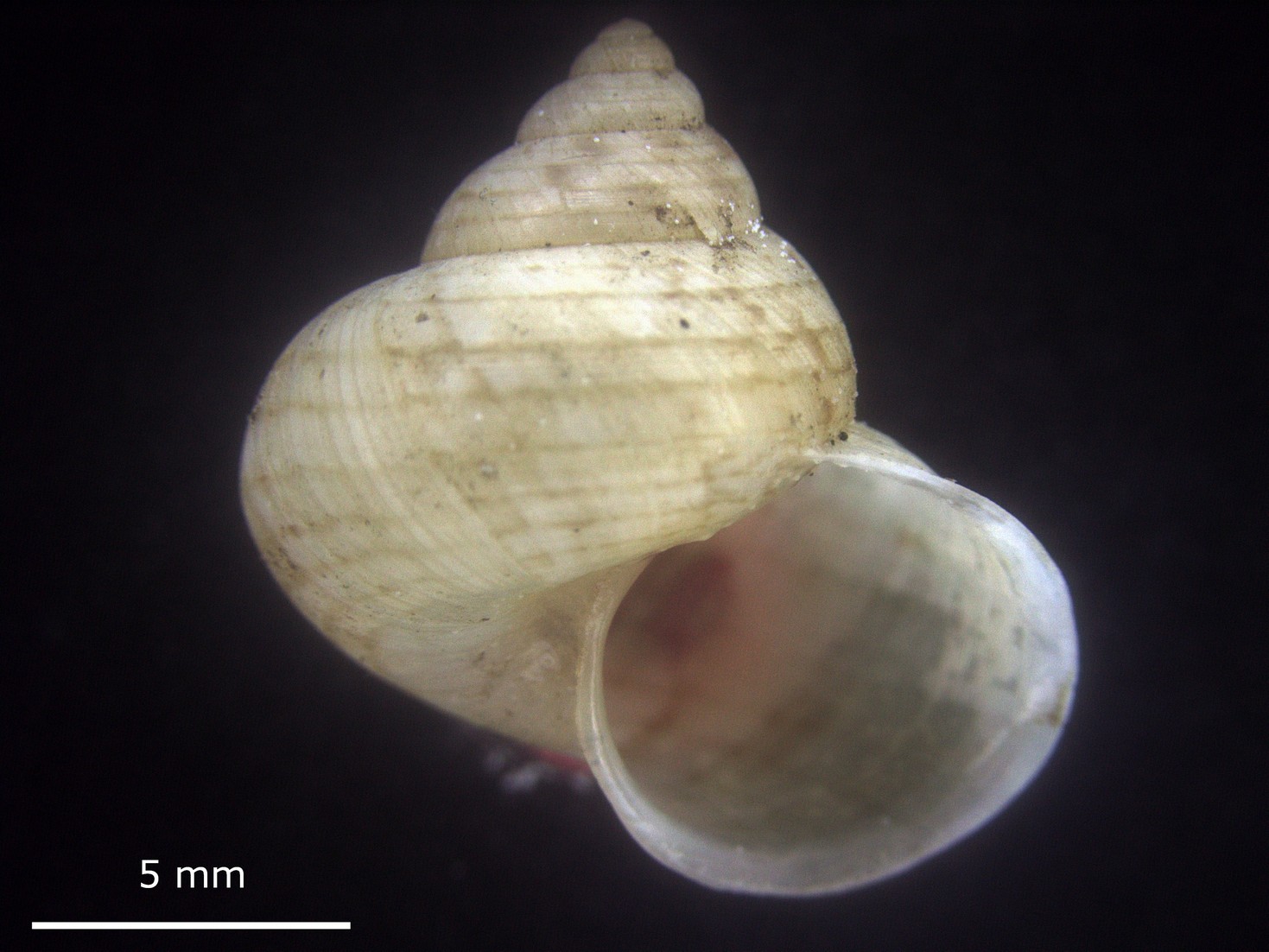

Supplement: File S4 [file peerj-10-13501-s004.zip › New Folder/8753.1.jpg]

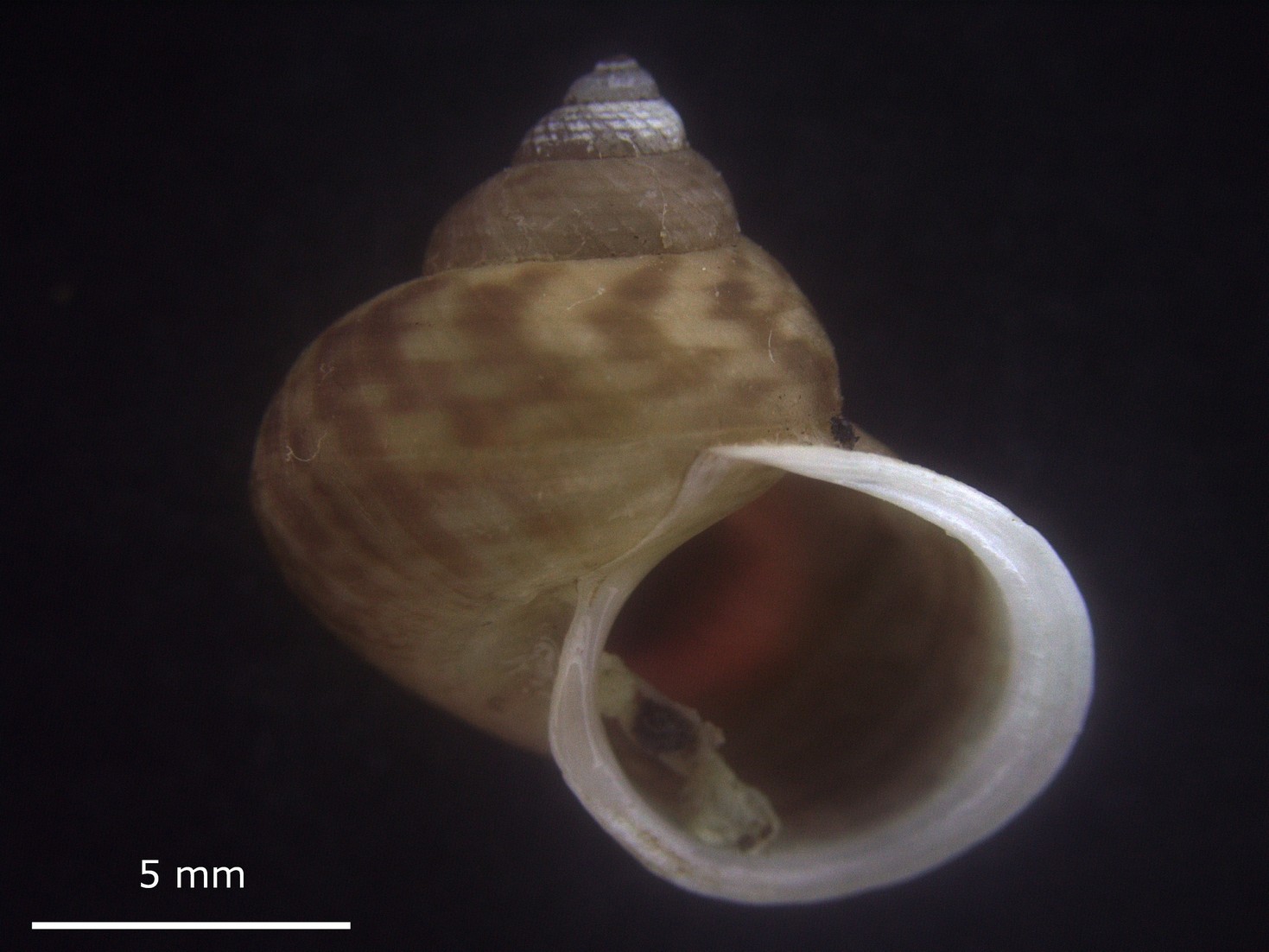

Supplement: File S4 [file peerj-10-13501-s004.zip › New Folder/8754.1.jpg]

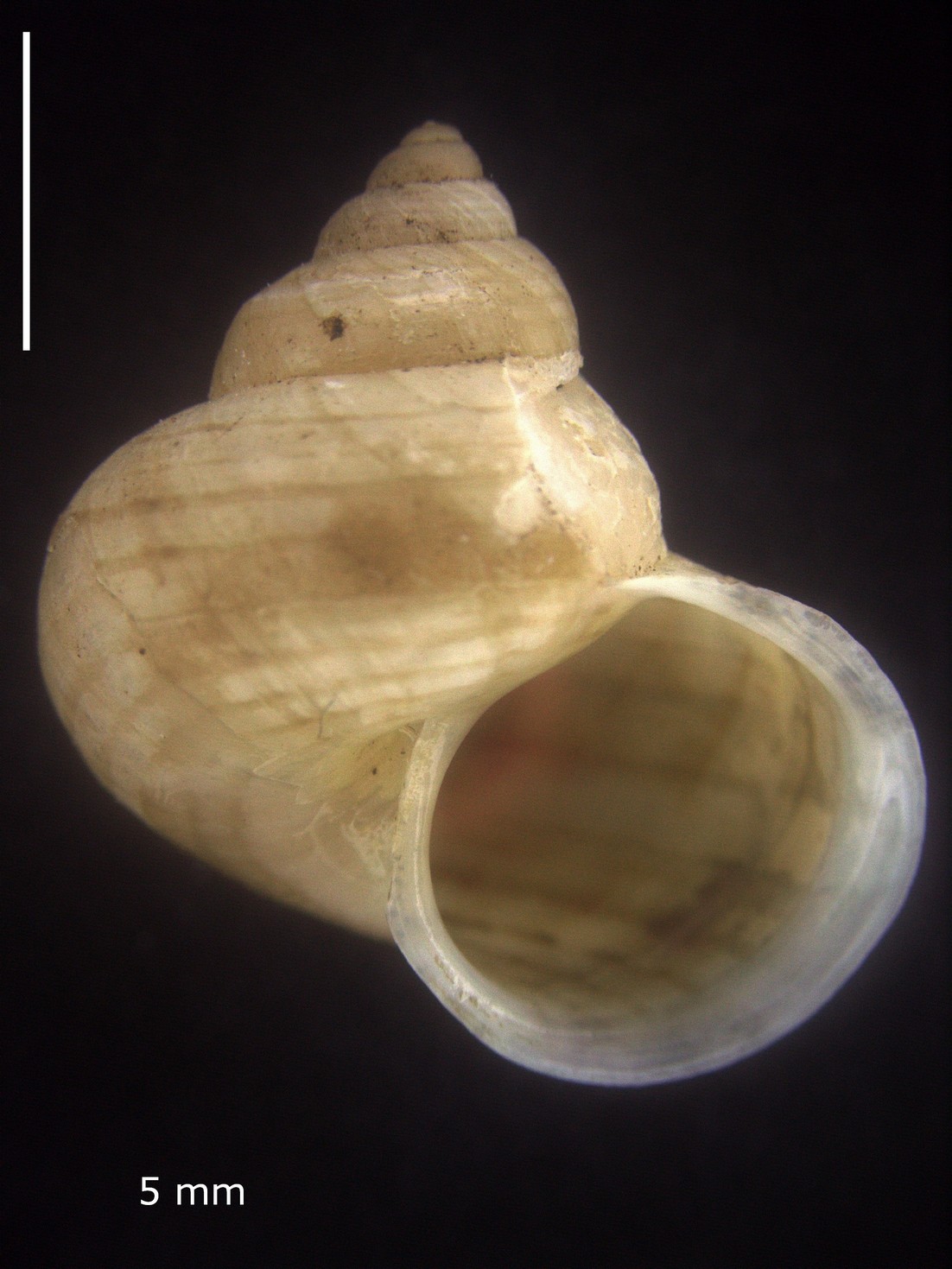

Supplement: File S4 [file peerj-10-13501-s004.zip › New Folder/8756.1.jpg]

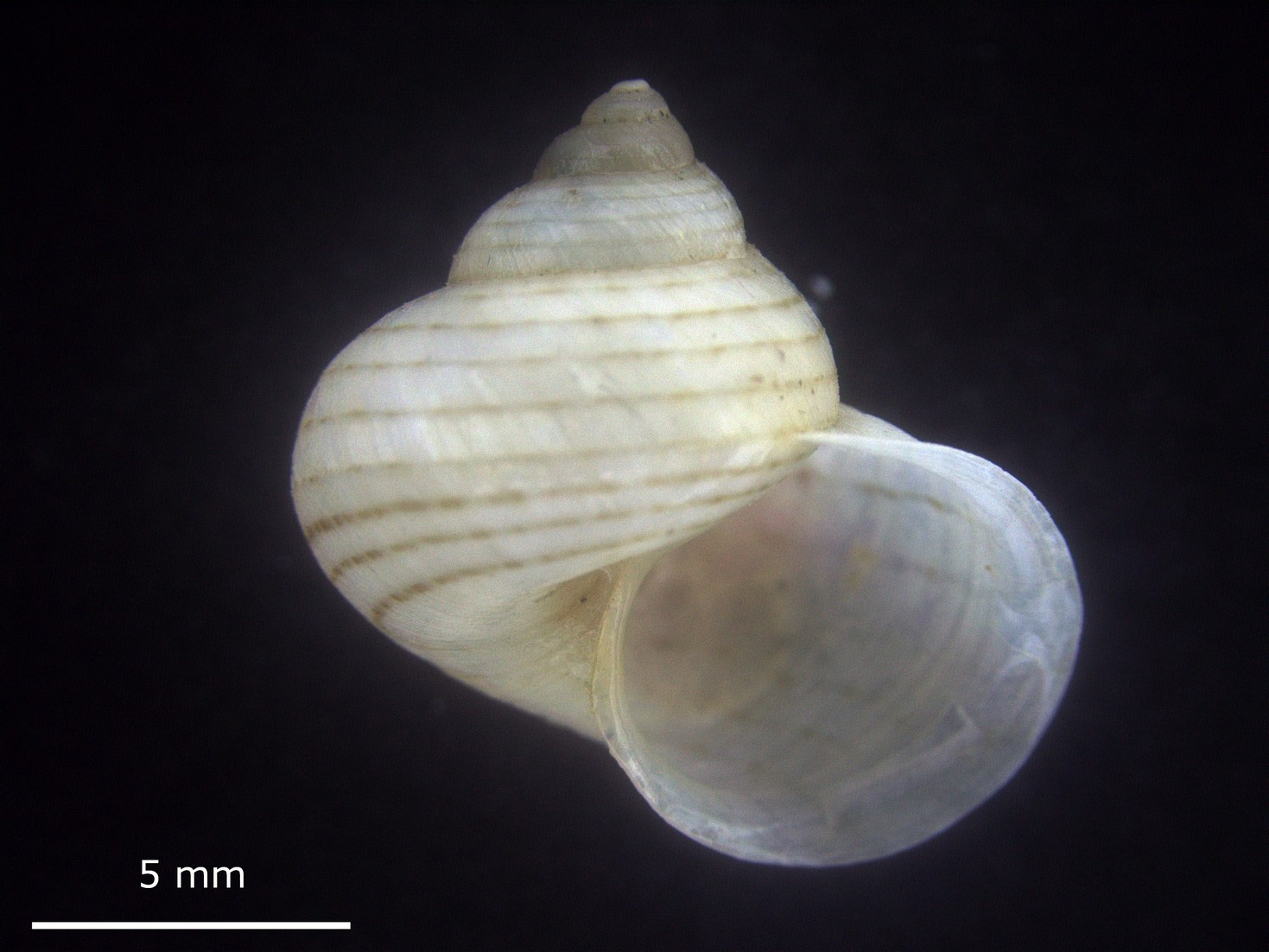

Supplement: File S4 [file peerj-10-13501-s004.zip › New Folder/8757.1.jpg]

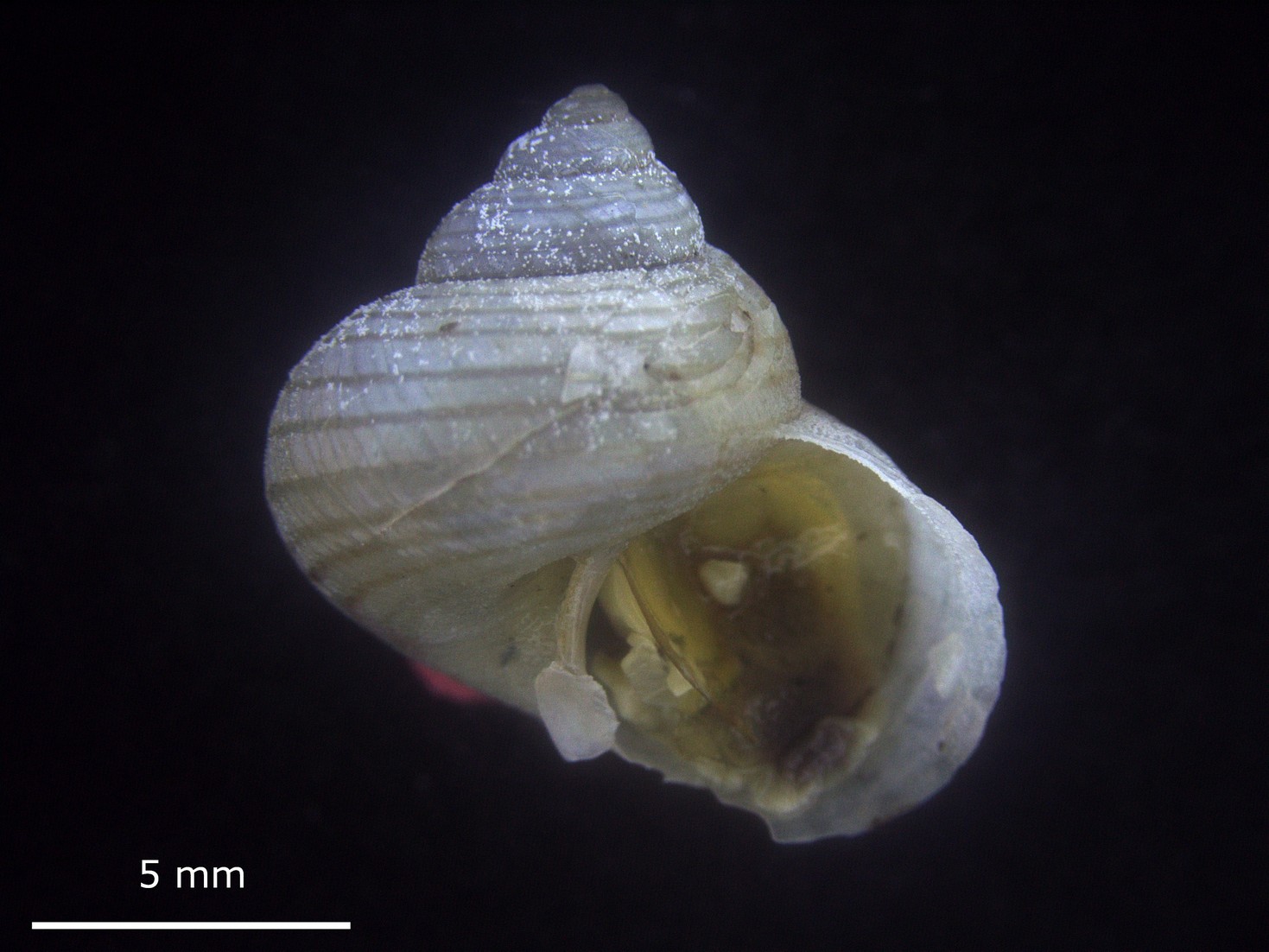

Supplement: File S4 [file peerj-10-13501-s004.zip › New Folder/8758.1.jpg]

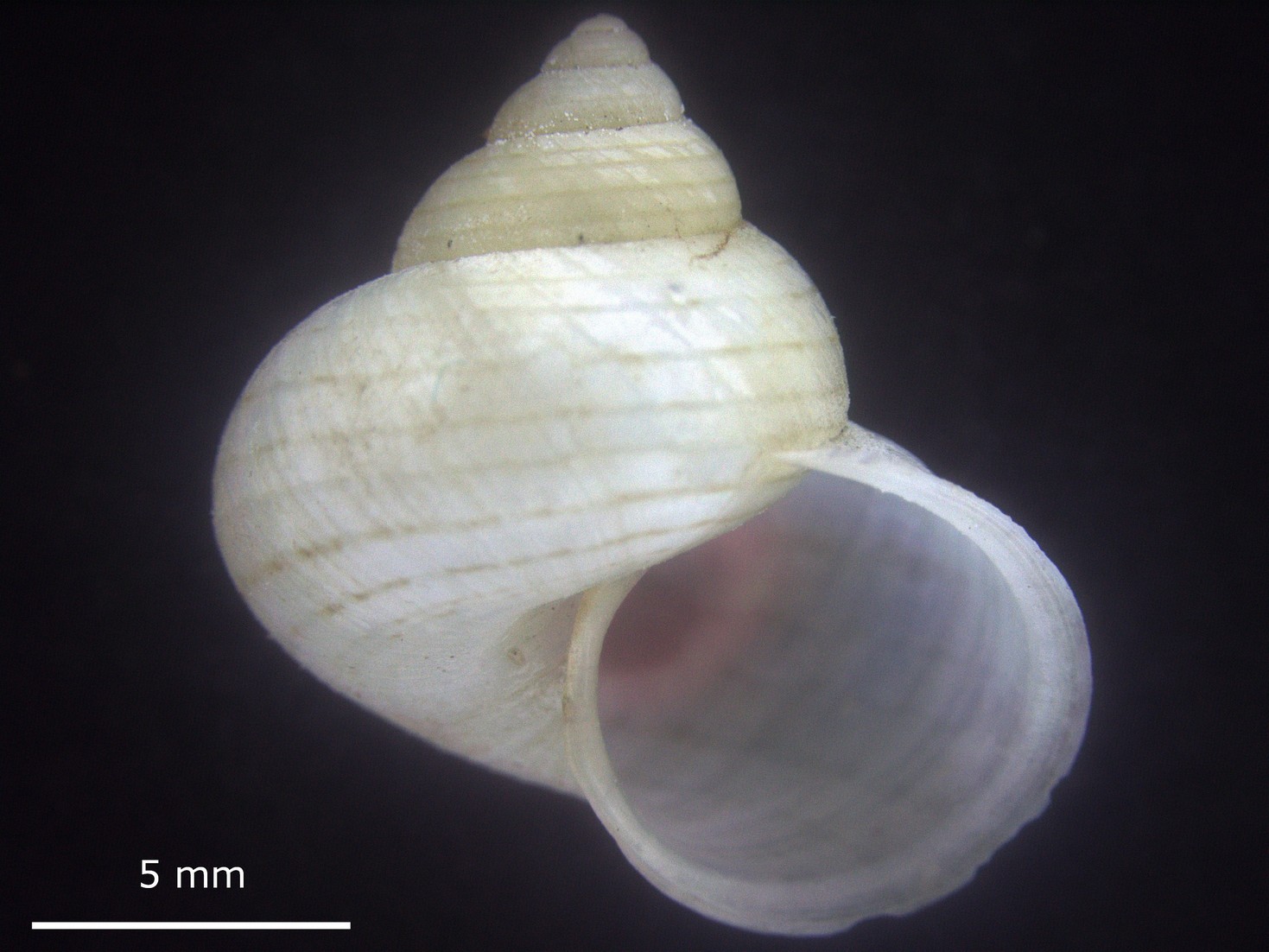

Supplement: File S4 [file peerj-10-13501-s004.zip › New Folder/8759.1.jpg]

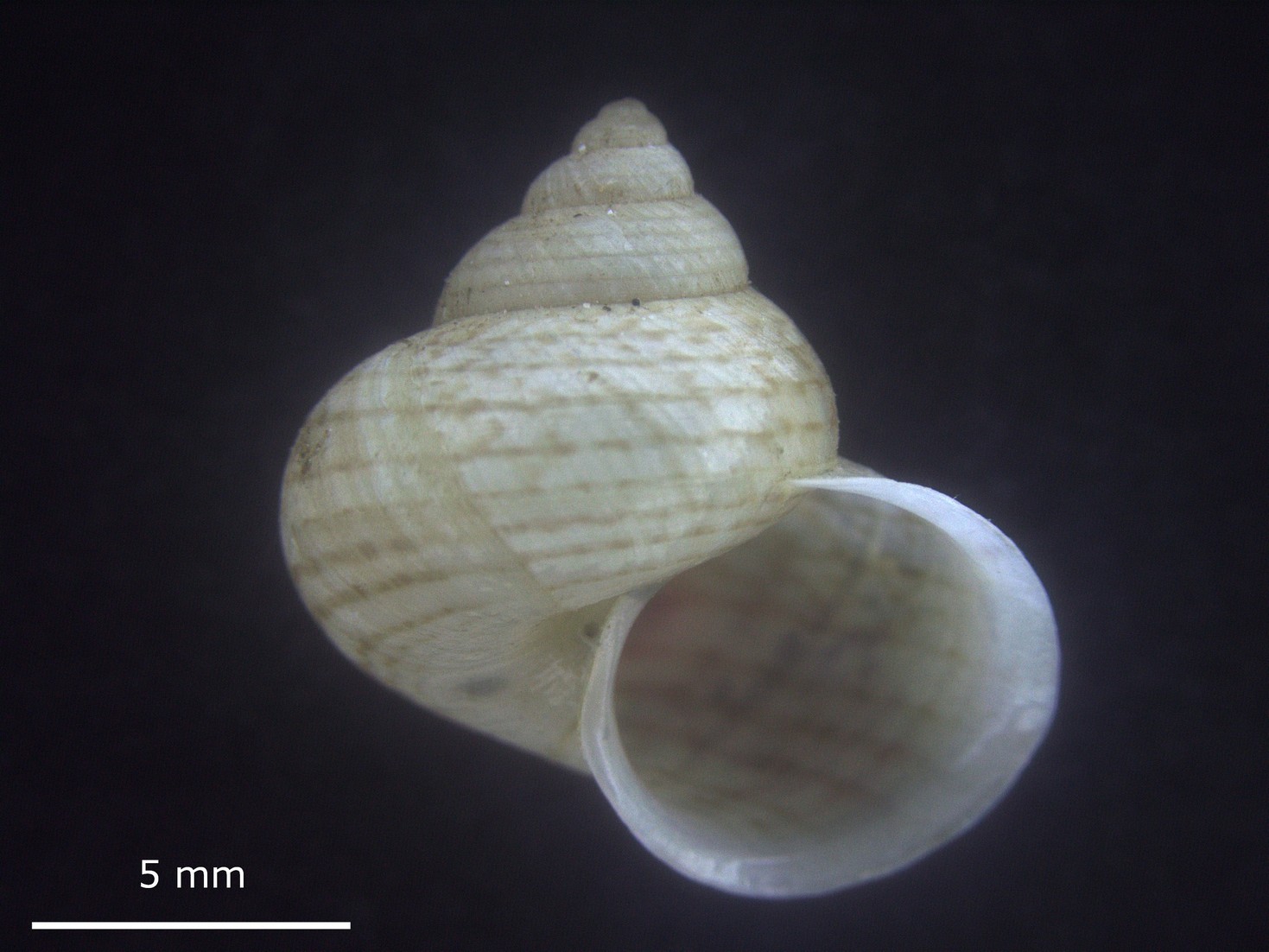

Supplement: File S4 [file peerj-10-13501-s004.zip › New Folder/8760.1.jpg]

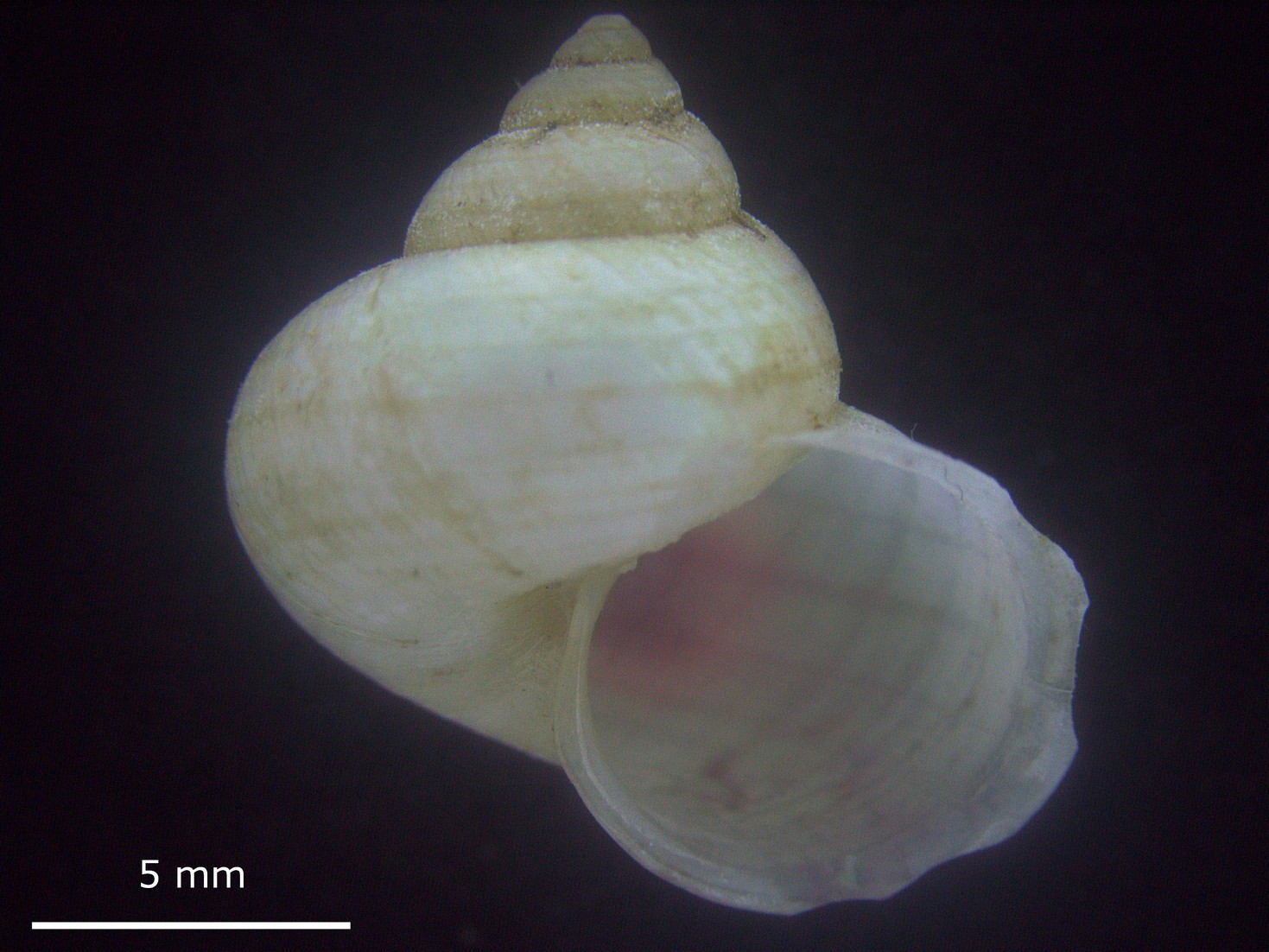

Supplement: File S4 [file peerj-10-13501-s004.zip › New Folder/8761.1.jpg]

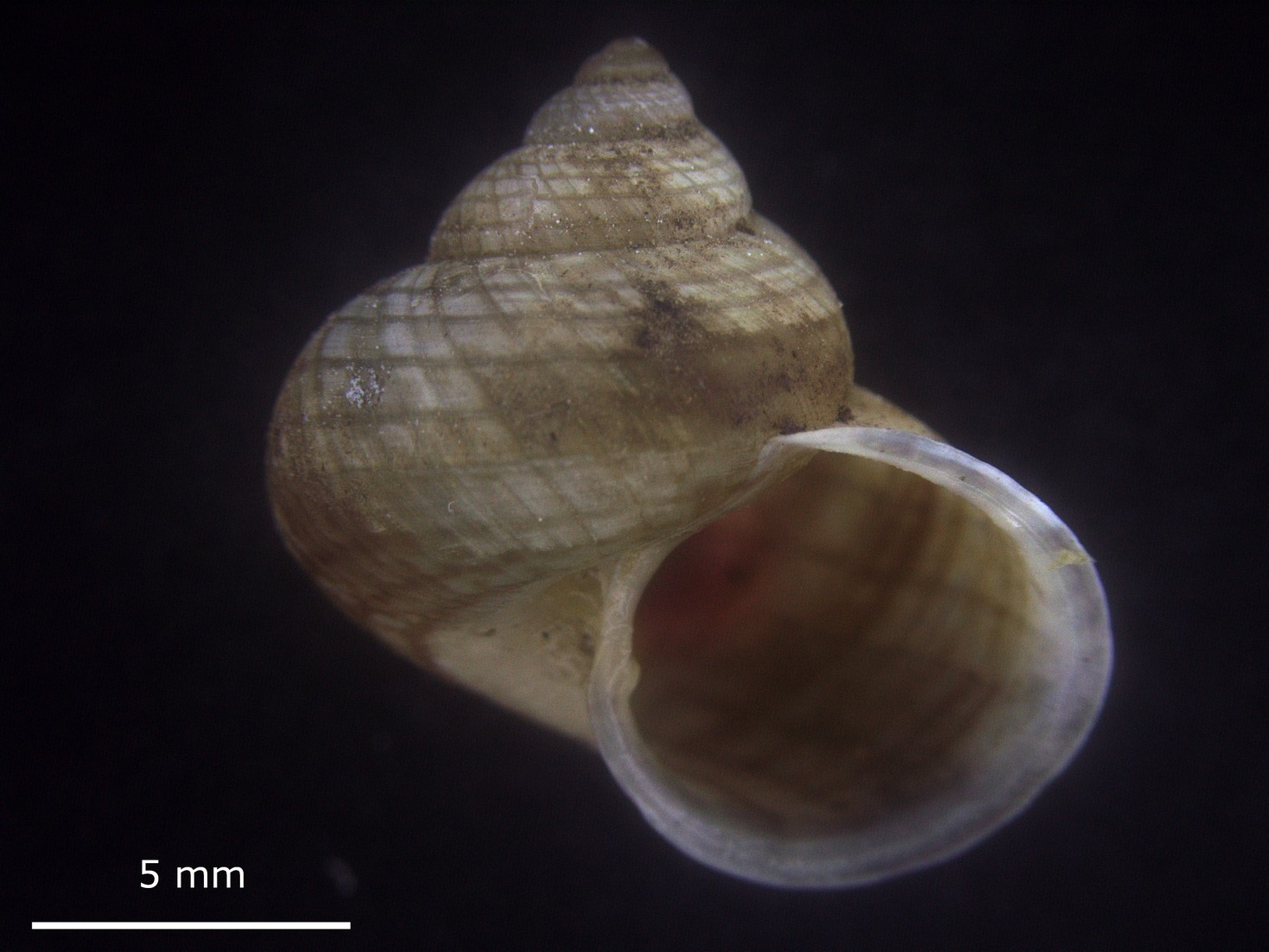

Supplement: File S4 [file peerj-10-13501-s004.zip › New Folder/8762.1.jpg]

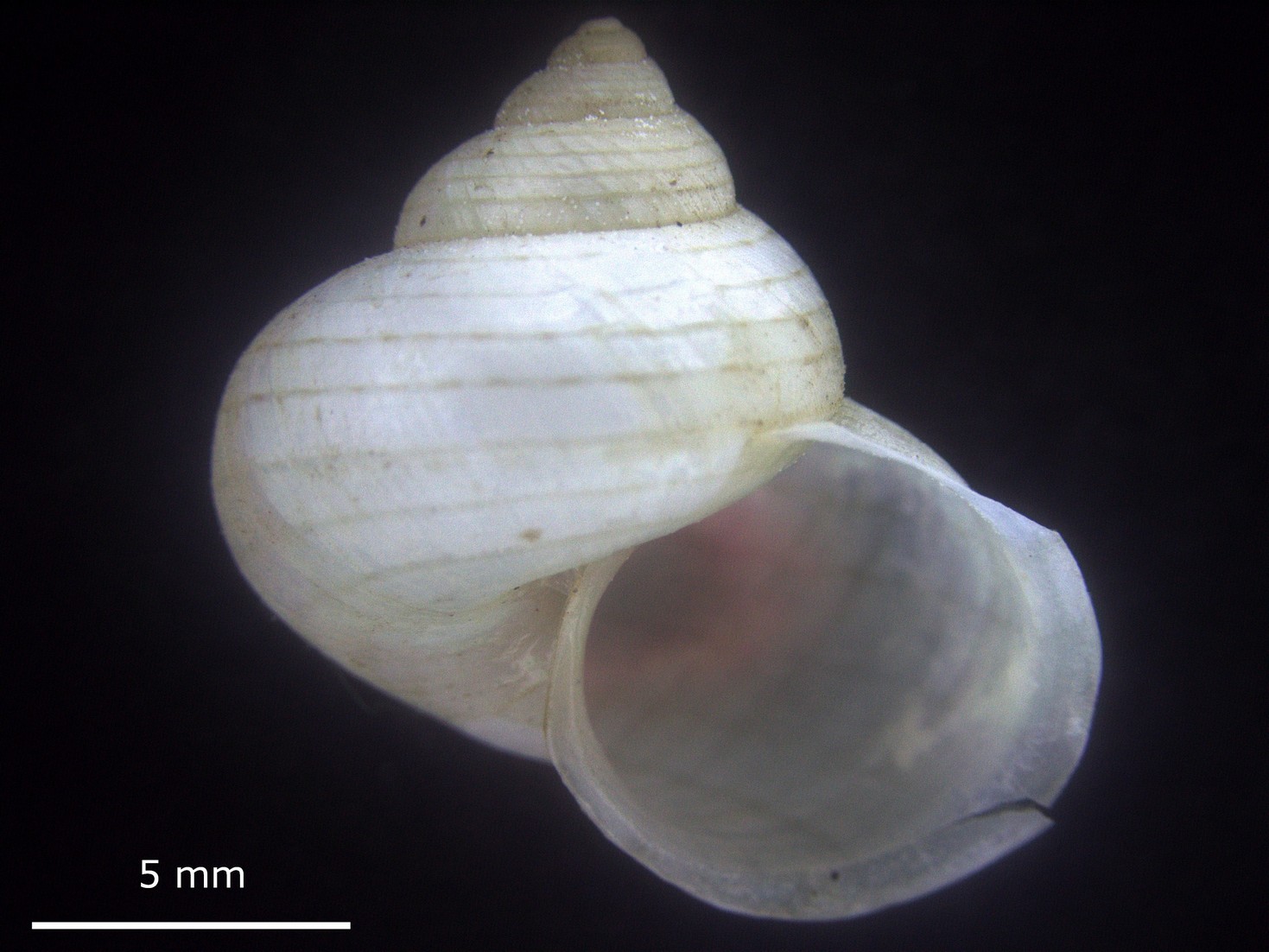

Supplement: File S4 [file peerj-10-13501-s004.zip › New Folder/8763.1.jpg]

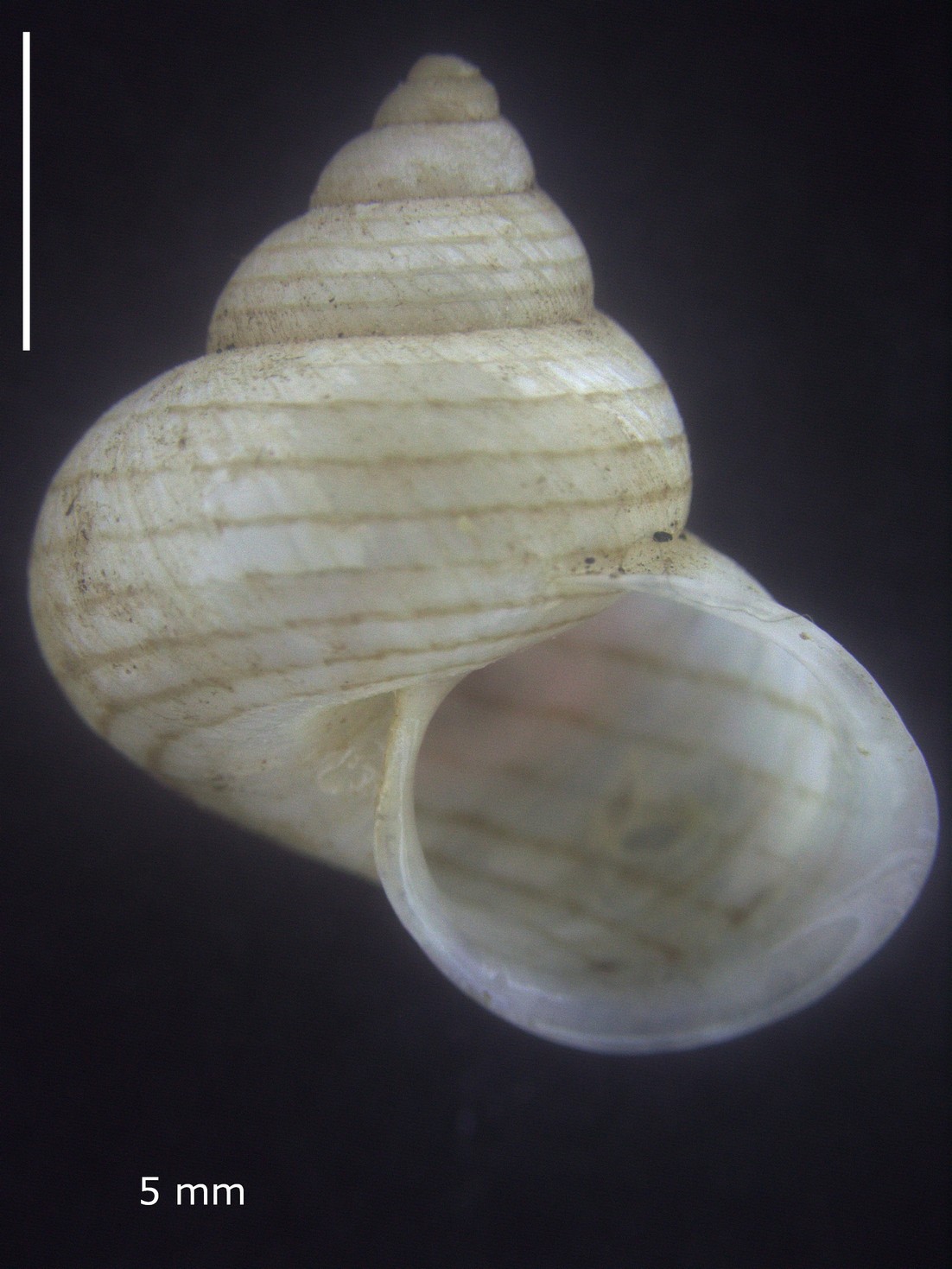

Supplement: File S4 [file peerj-10-13501-s004.zip › New Folder/8764.1.jpg]

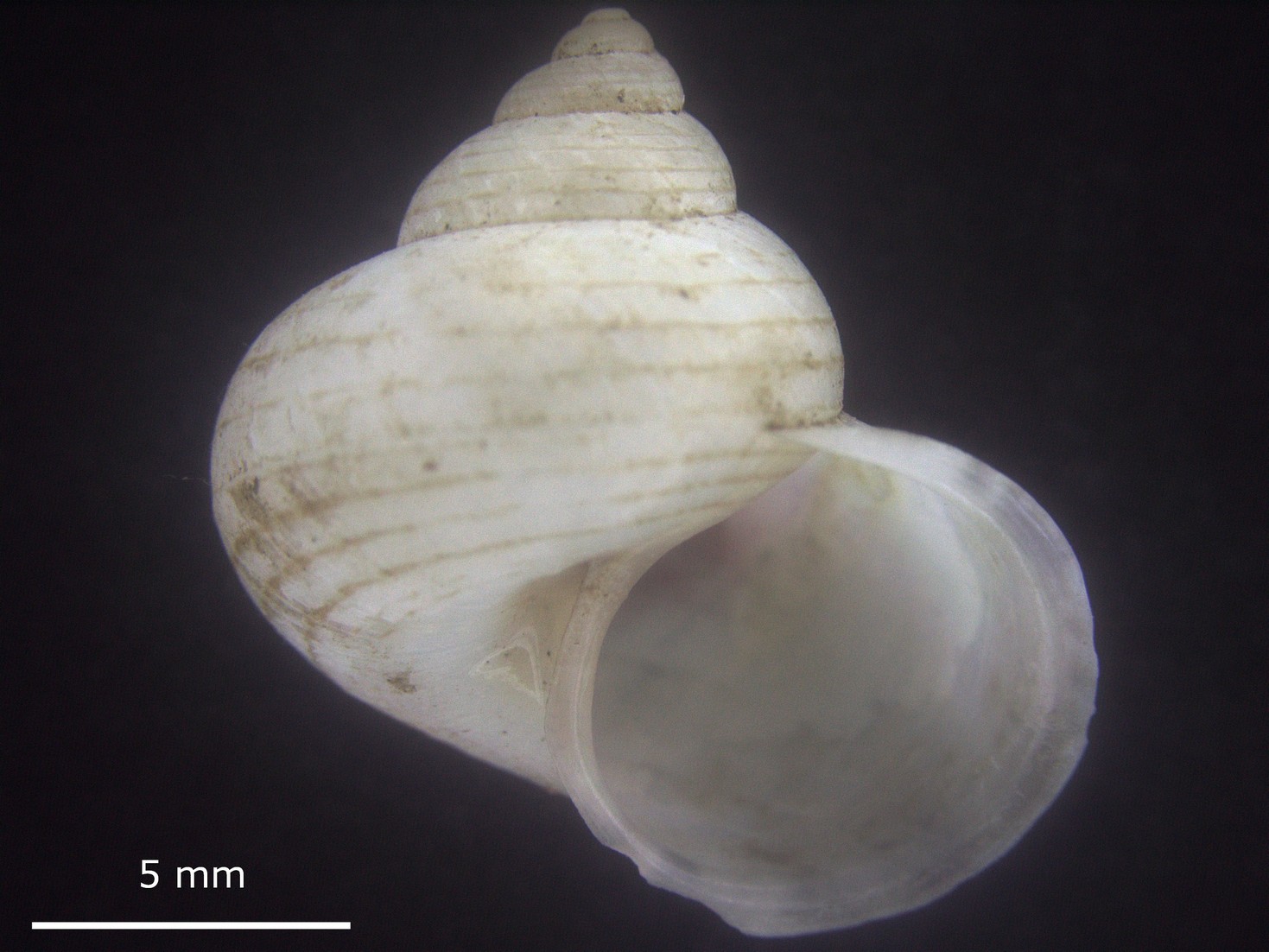

Supplement: File S4 [file peerj-10-13501-s004.zip › New Folder/8765.1.jpg]

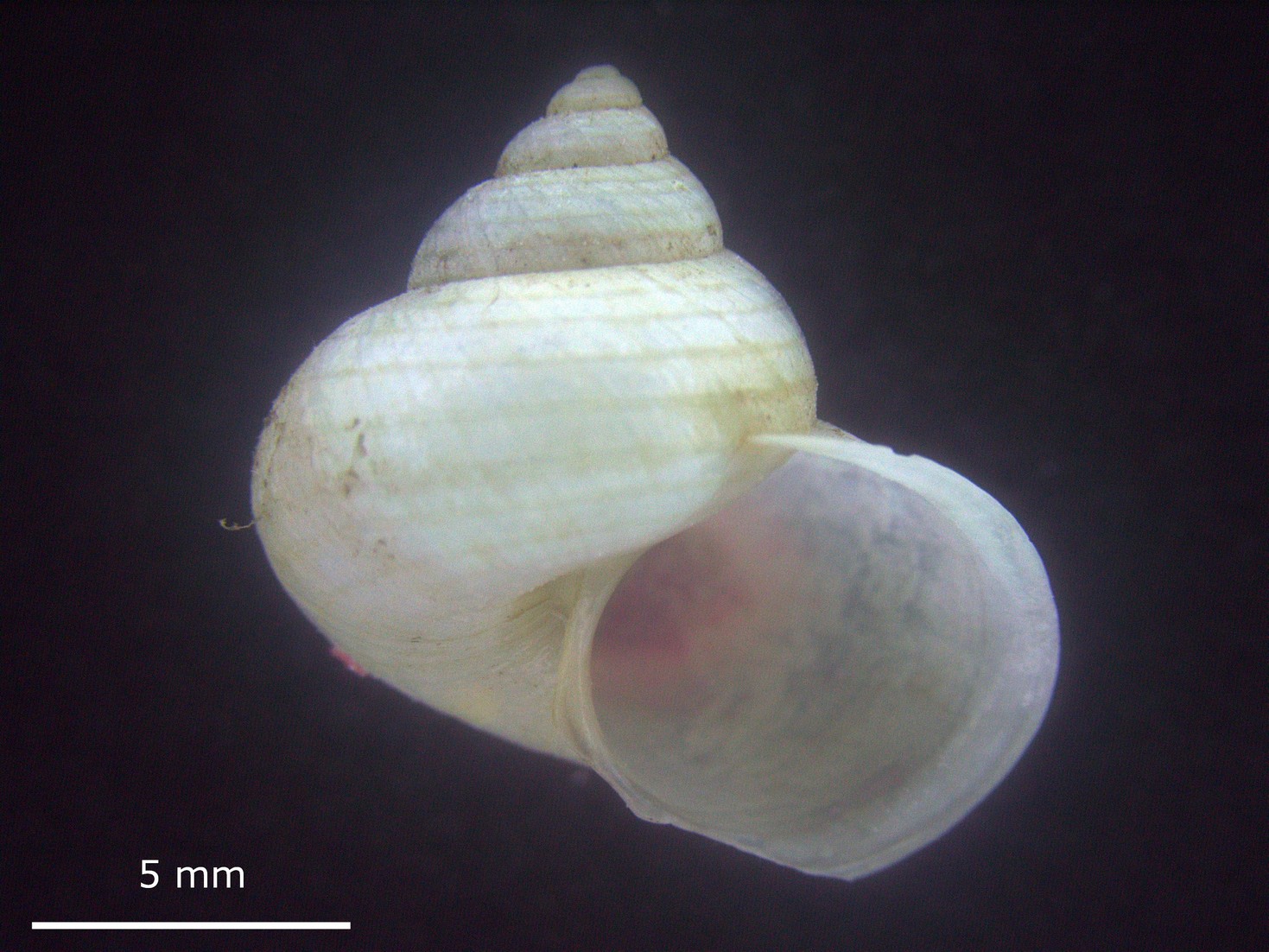

Supplement: File S4 [file peerj-10-13501-s004.zip › New Folder/8766.1.jpg]

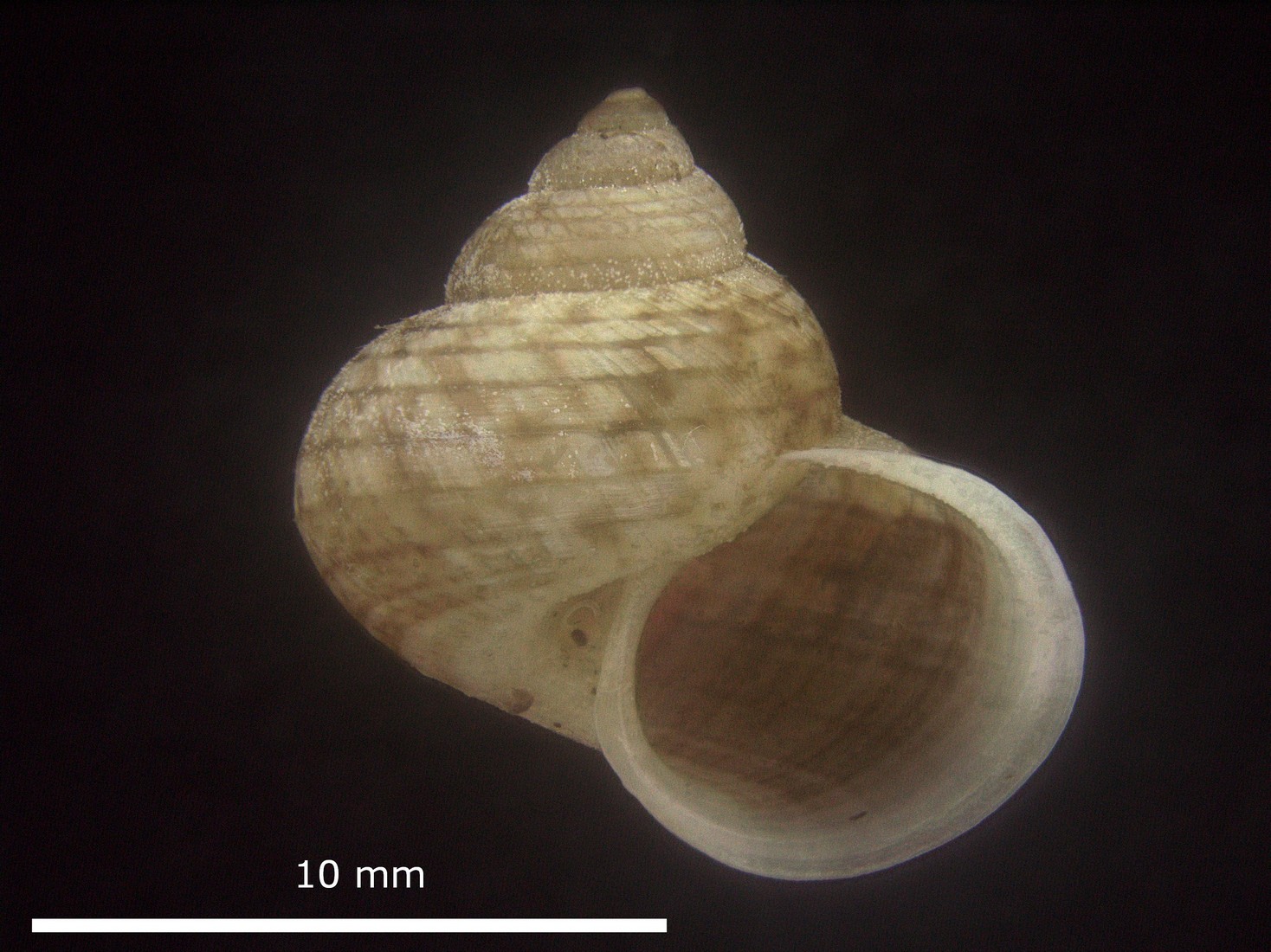

Supplement: File S4 [file peerj-10-13501-s004.zip › New Folder/8767.1.jpg]

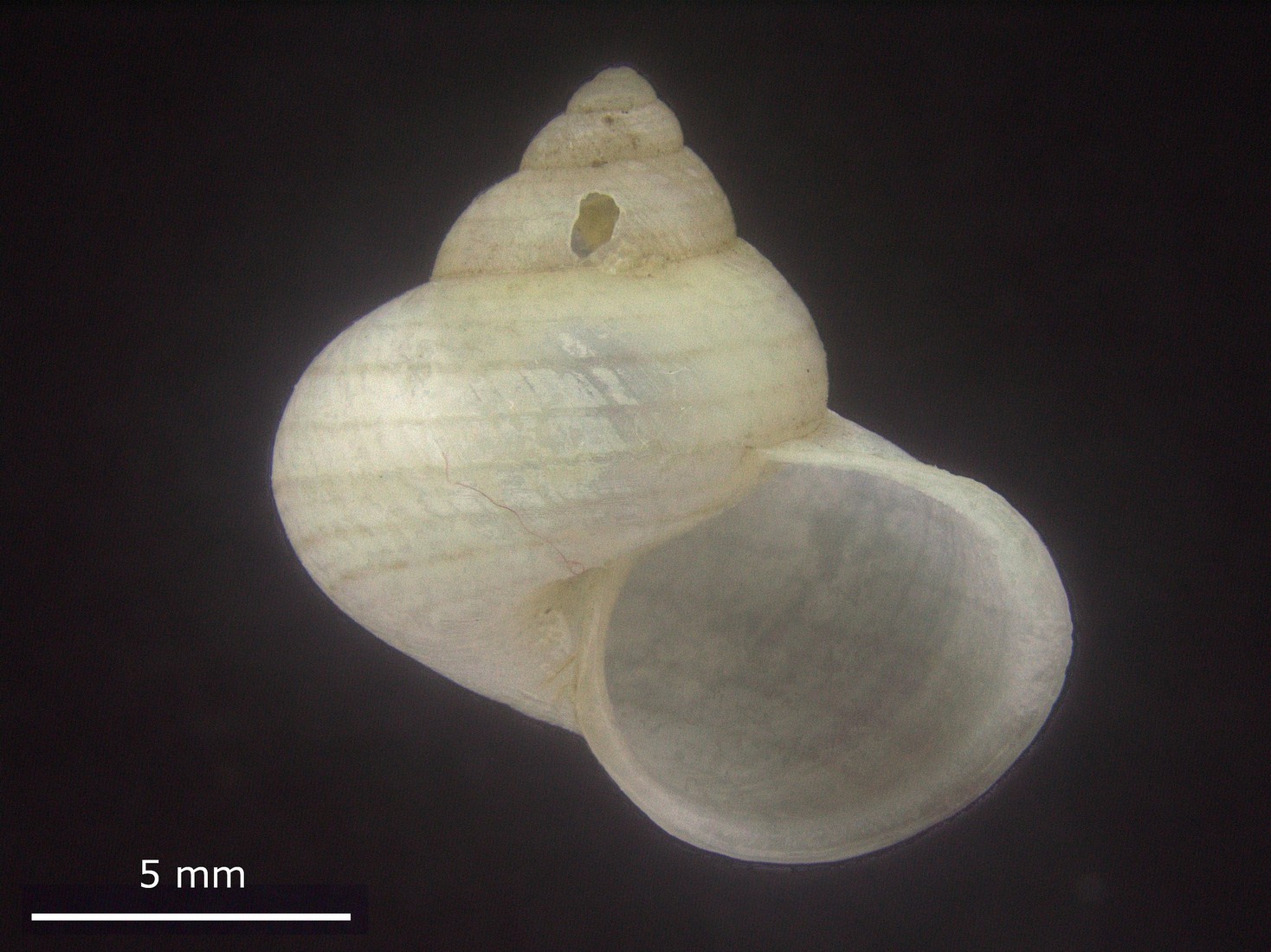

Supplement: File S4 [file peerj-10-13501-s004.zip › New Folder/8768.1.jpg]

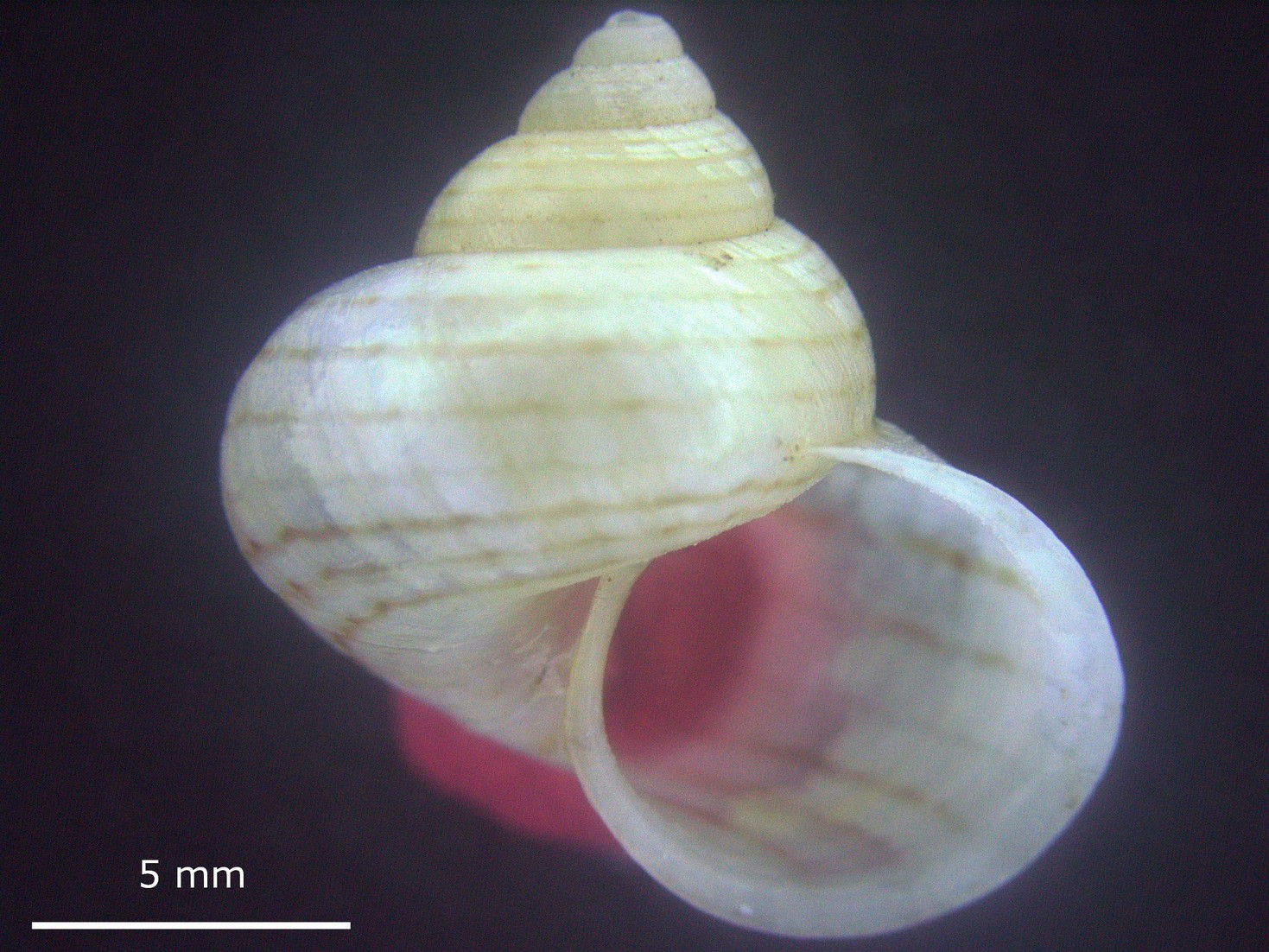

Supplement: File S4 [file peerj-10-13501-s004.zip › New Folder/8769.1.jpg]

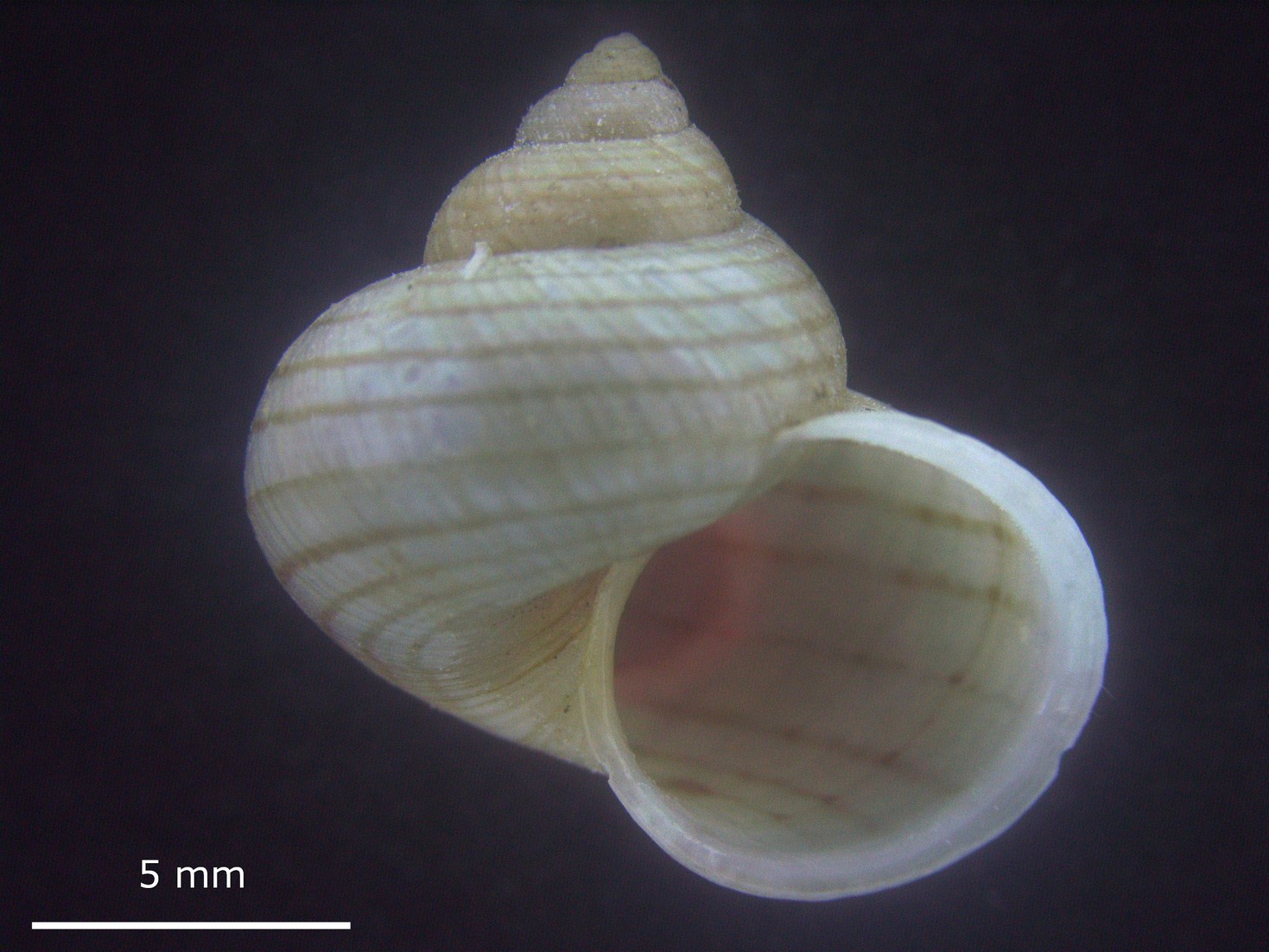

Supplement: File S4 [file peerj-10-13501-s004.zip › New Folder/8770.1.jpg]

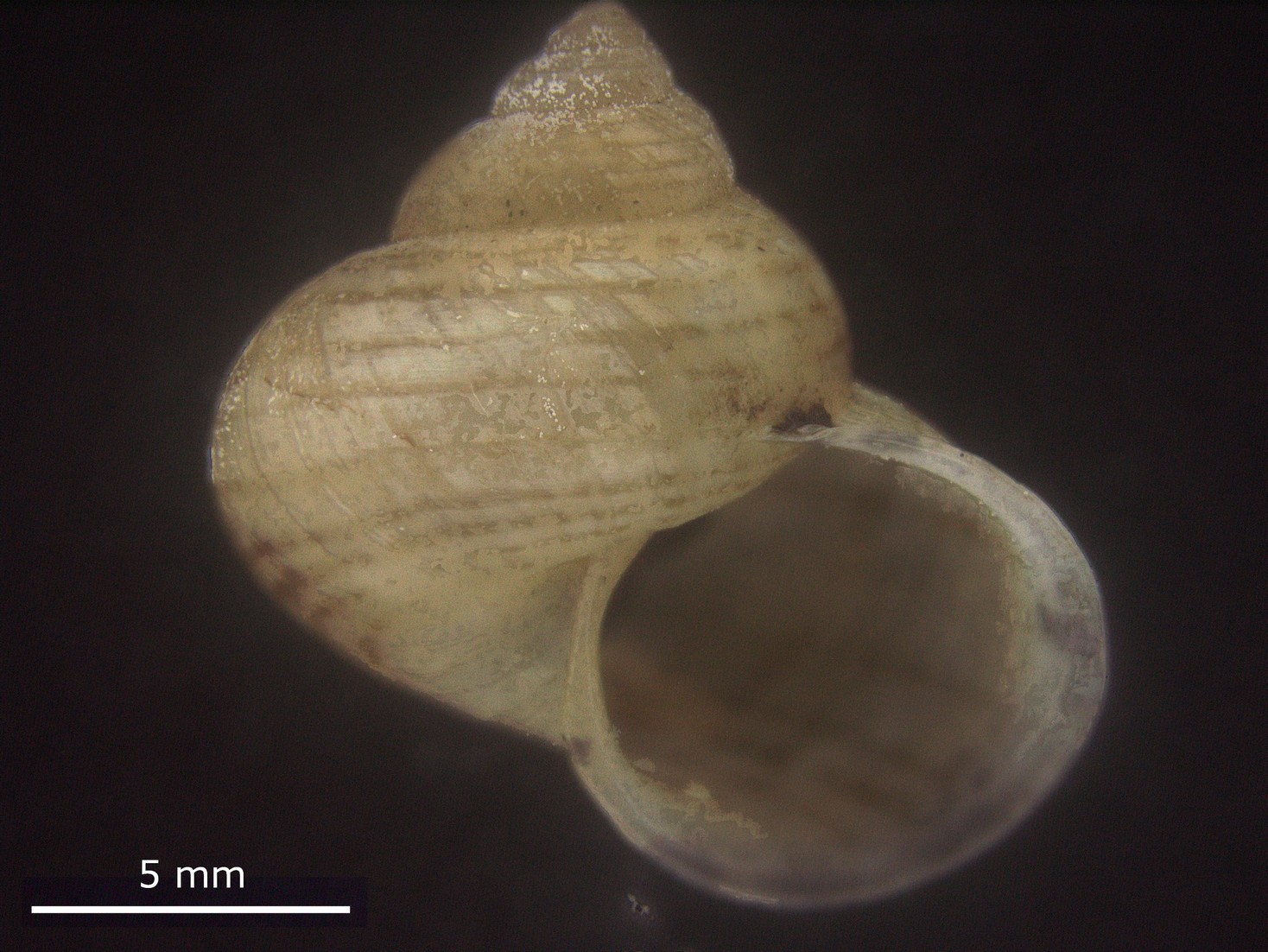

Supplement: File S4 [file peerj-10-13501-s004.zip › New Folder/8771.1.jpg]

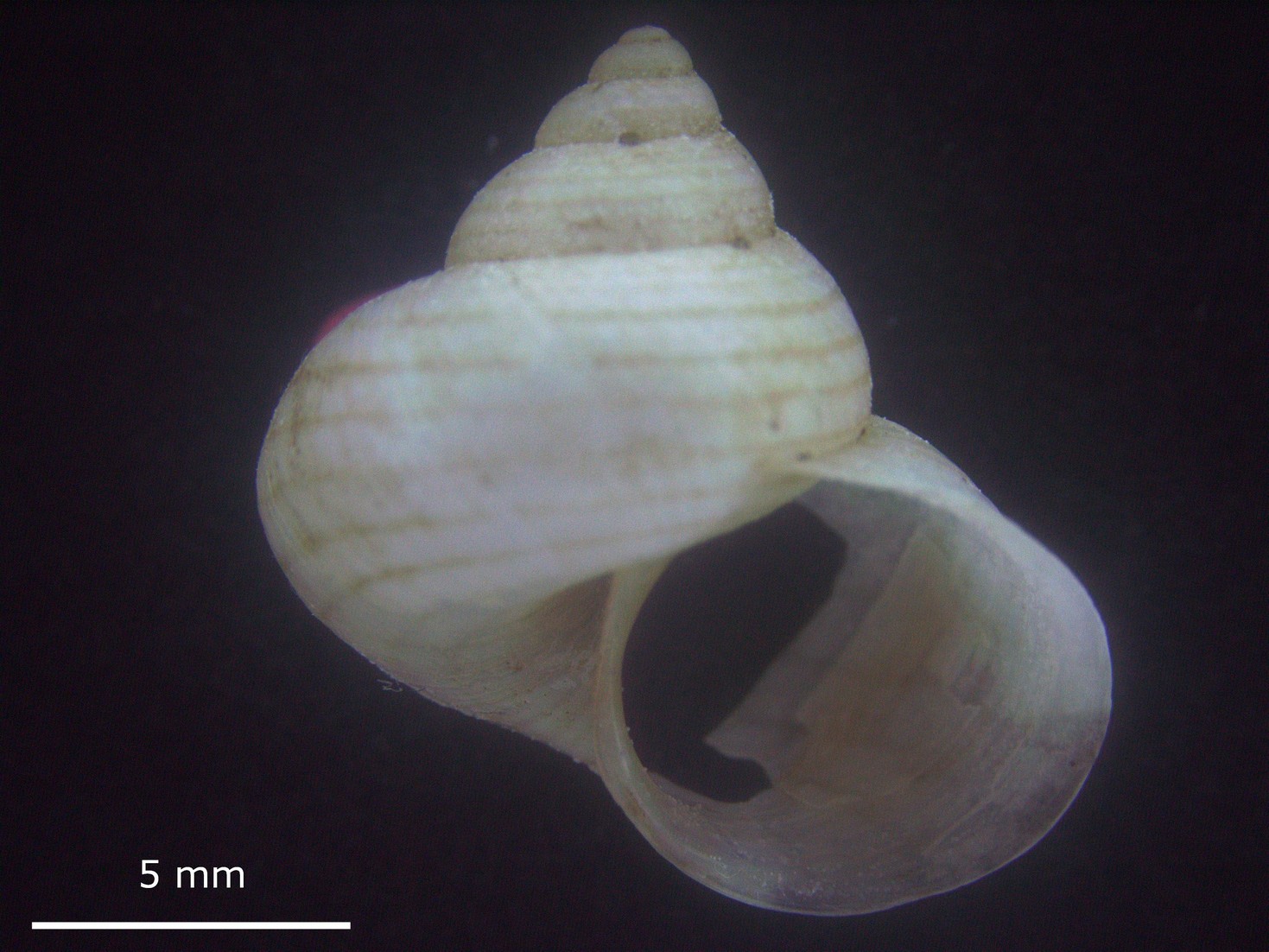

Supplement: File S4 [file peerj-10-13501-s004.zip › New Folder/8772.1.jpg]

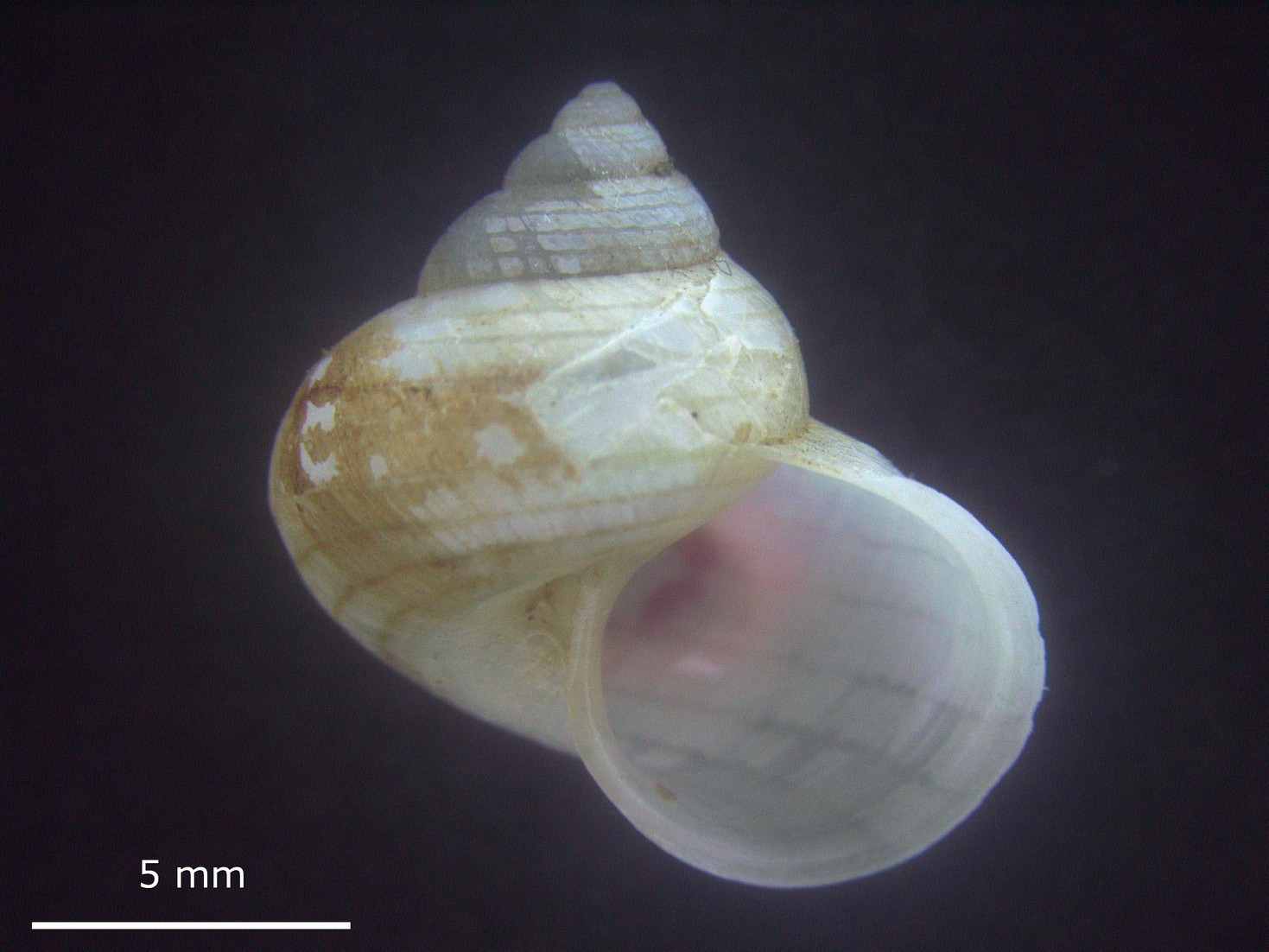

Supplement: File S4 [file peerj-10-13501-s004.zip › New Folder/8773.1.jpg]

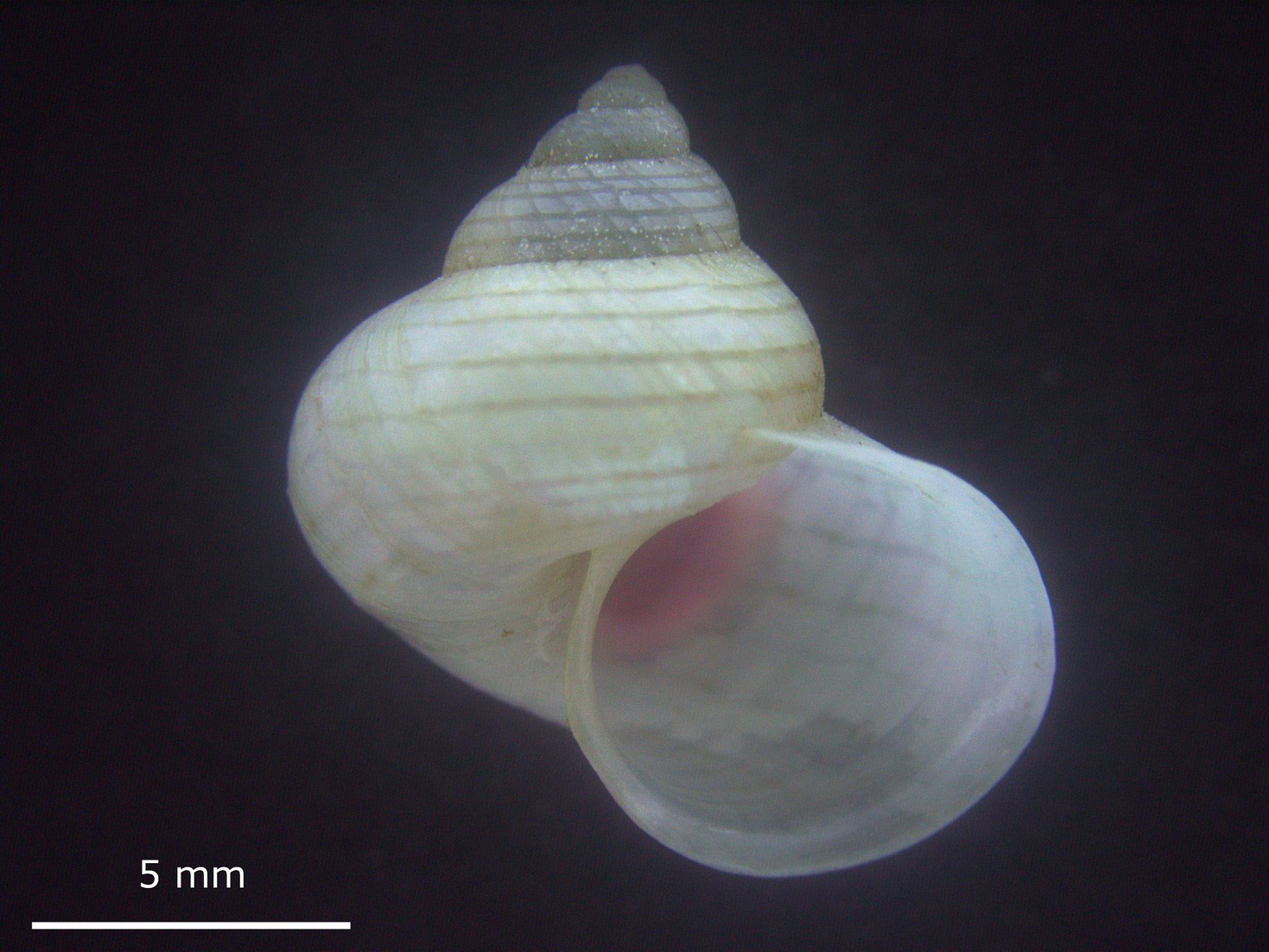

Supplement: File S4 [file peerj-10-13501-s004.zip › New Folder/8774.1.jpg]

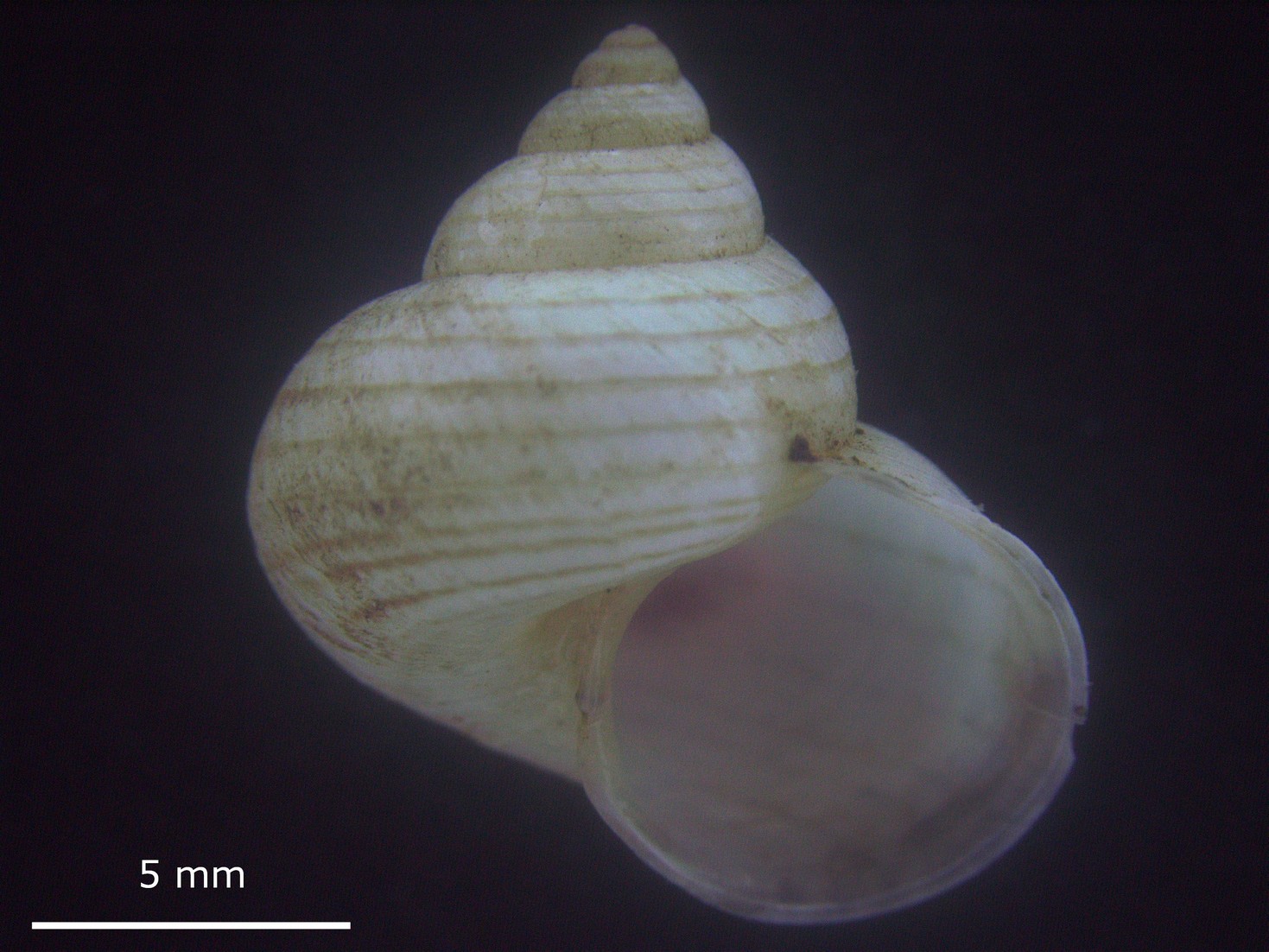

Supplement: File S4 [file peerj-10-13501-s004.zip › New Folder/8775.1.jpg]

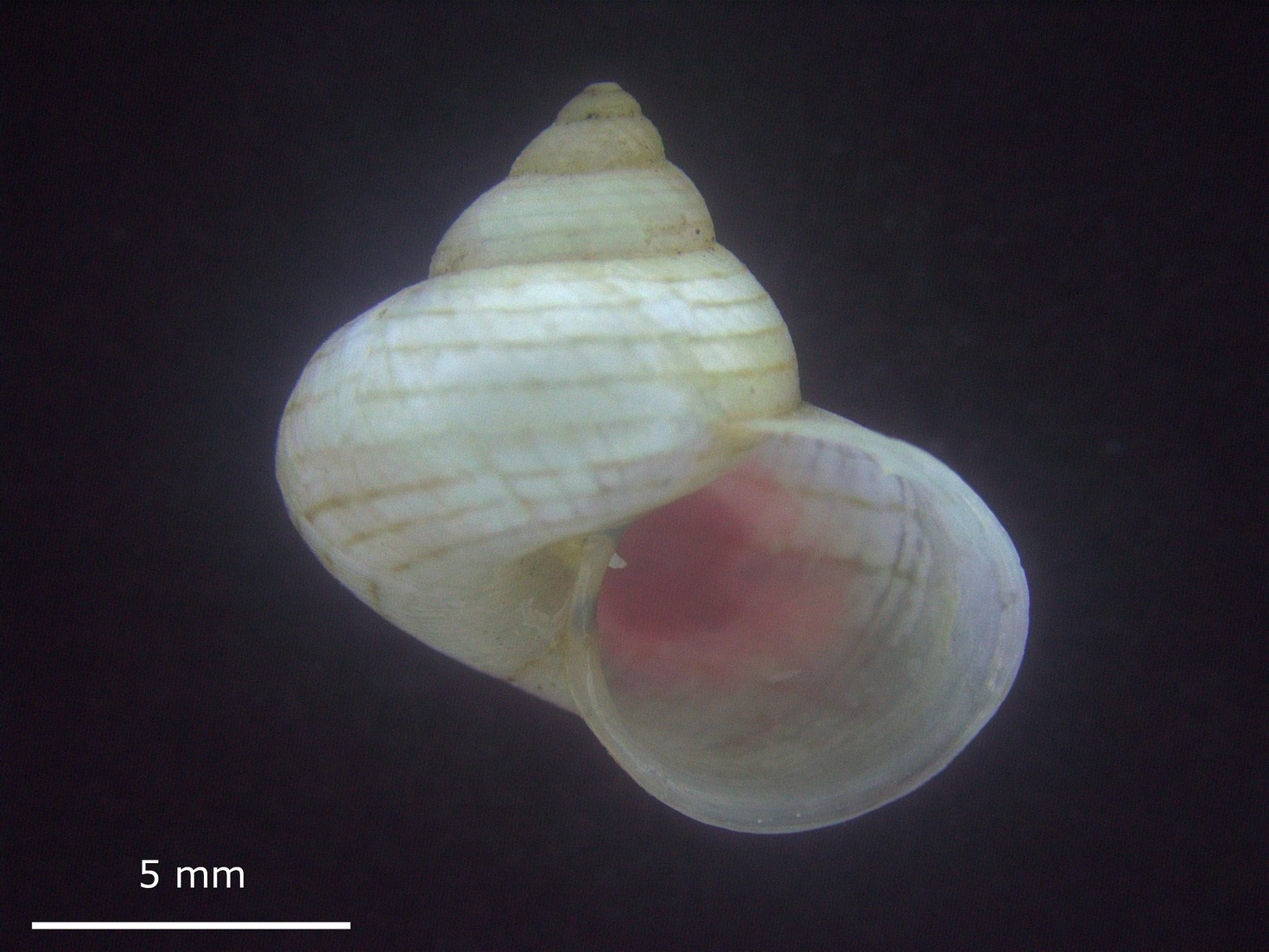

Supplement: File S4 [file peerj-10-13501-s004.zip › New Folder/8776.1.jpg]

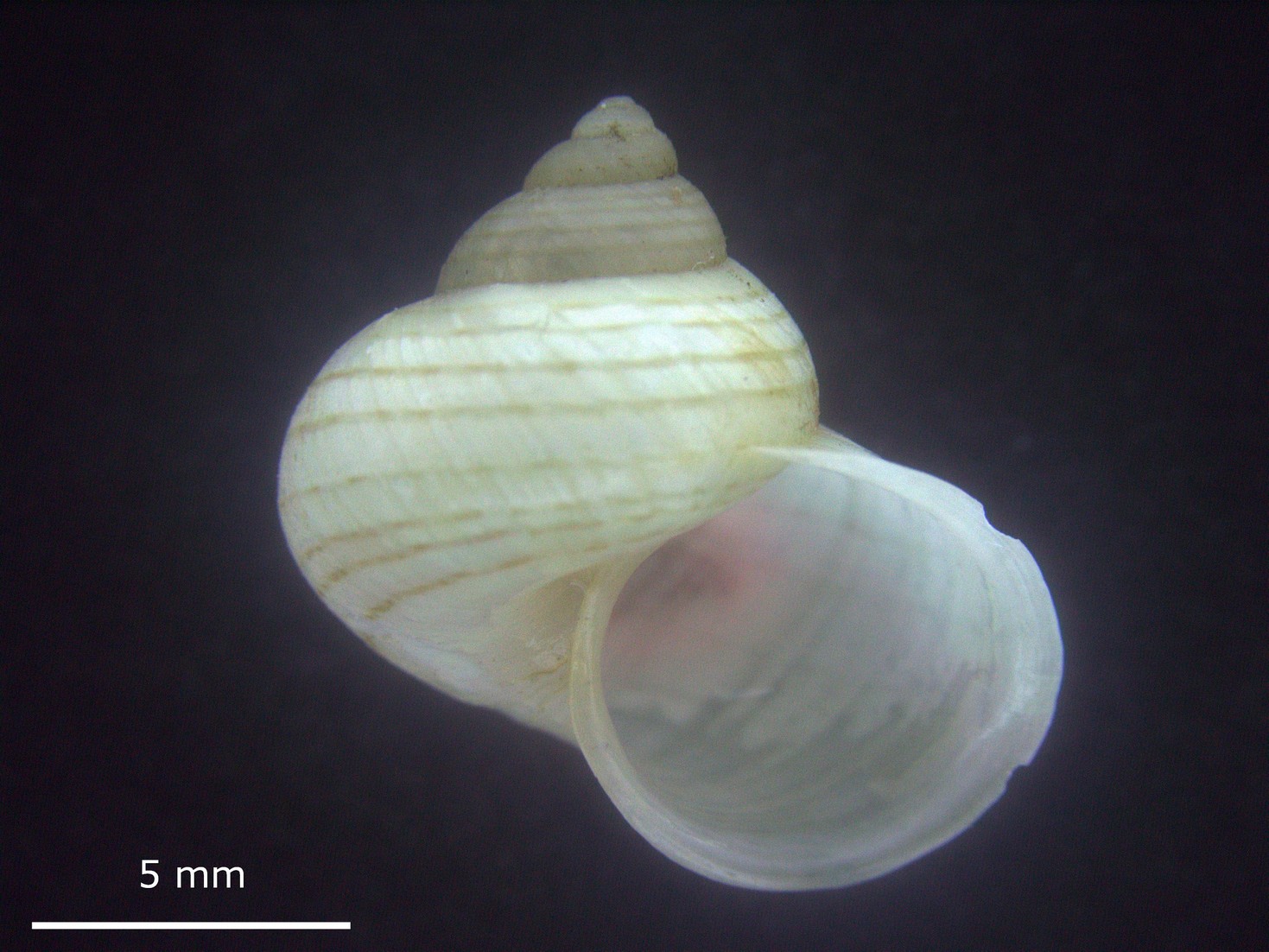

Supplement: File S4 [file peerj-10-13501-s004.zip › New Folder/8777.1.jpg]

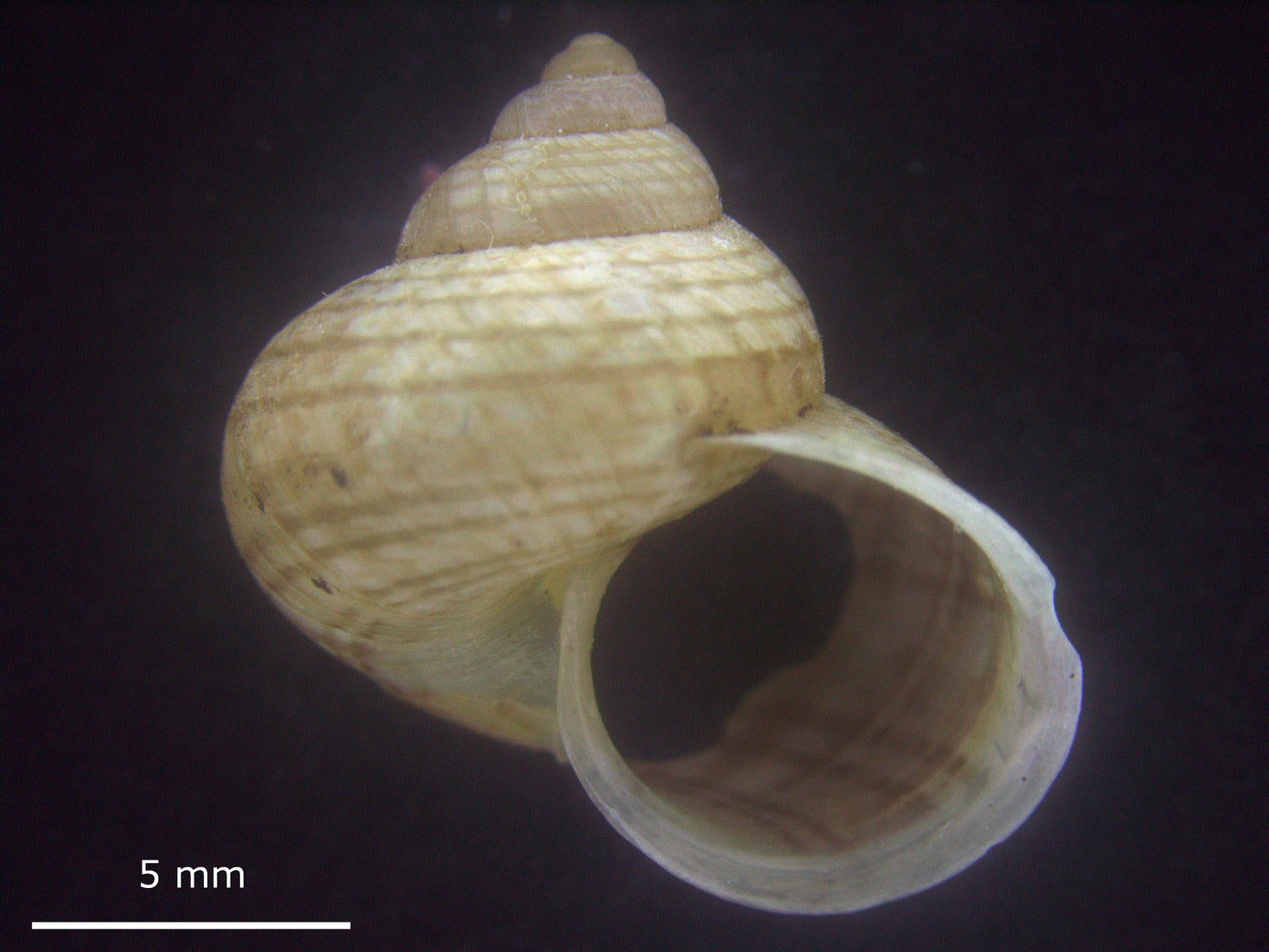

Supplement: File S4 [file peerj-10-13501-s004.zip › New Folder/8778.1.jpg]

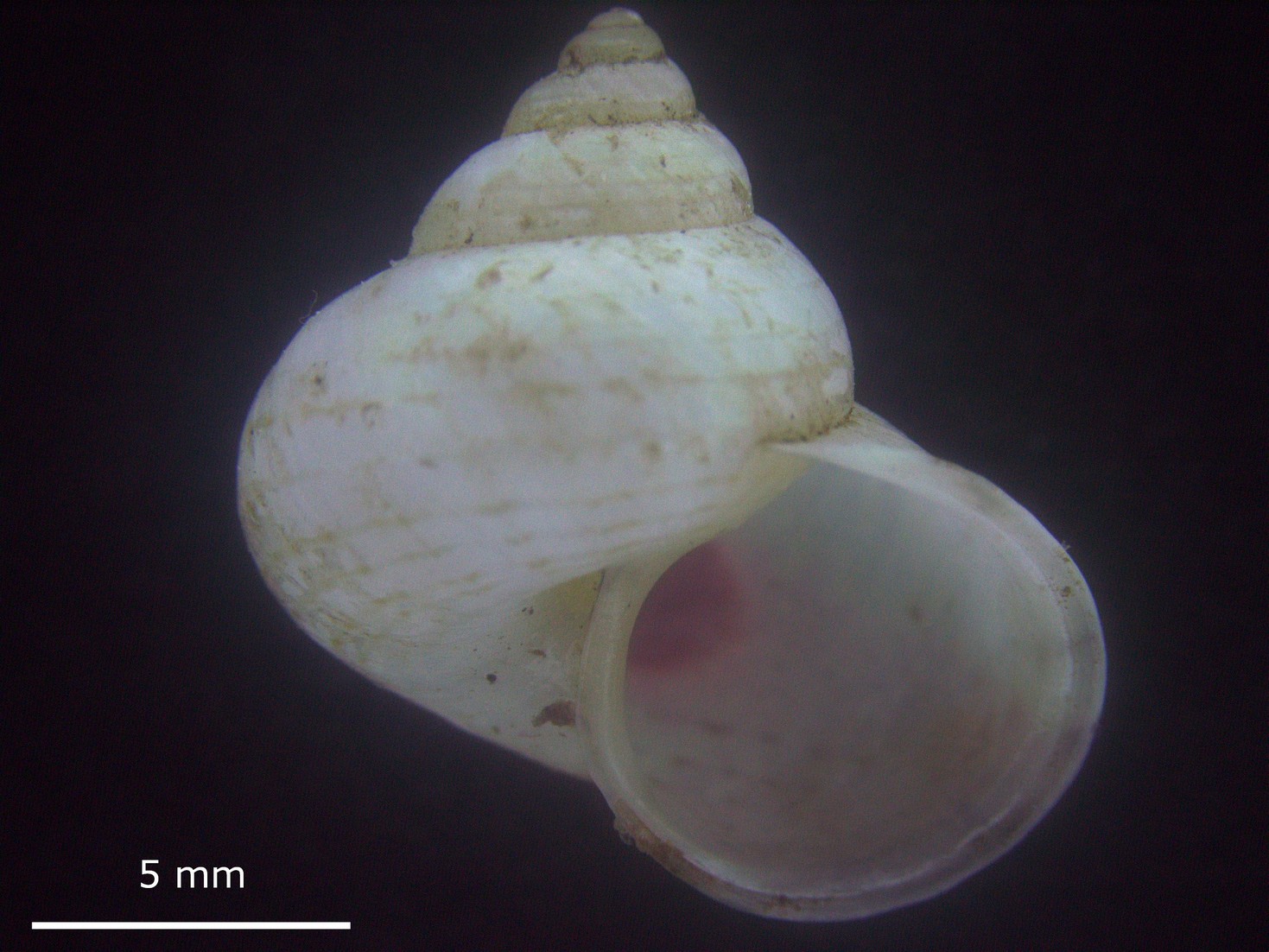

Supplement: File S4 [file peerj-10-13501-s004.zip › New Folder/8779.1.jpg]

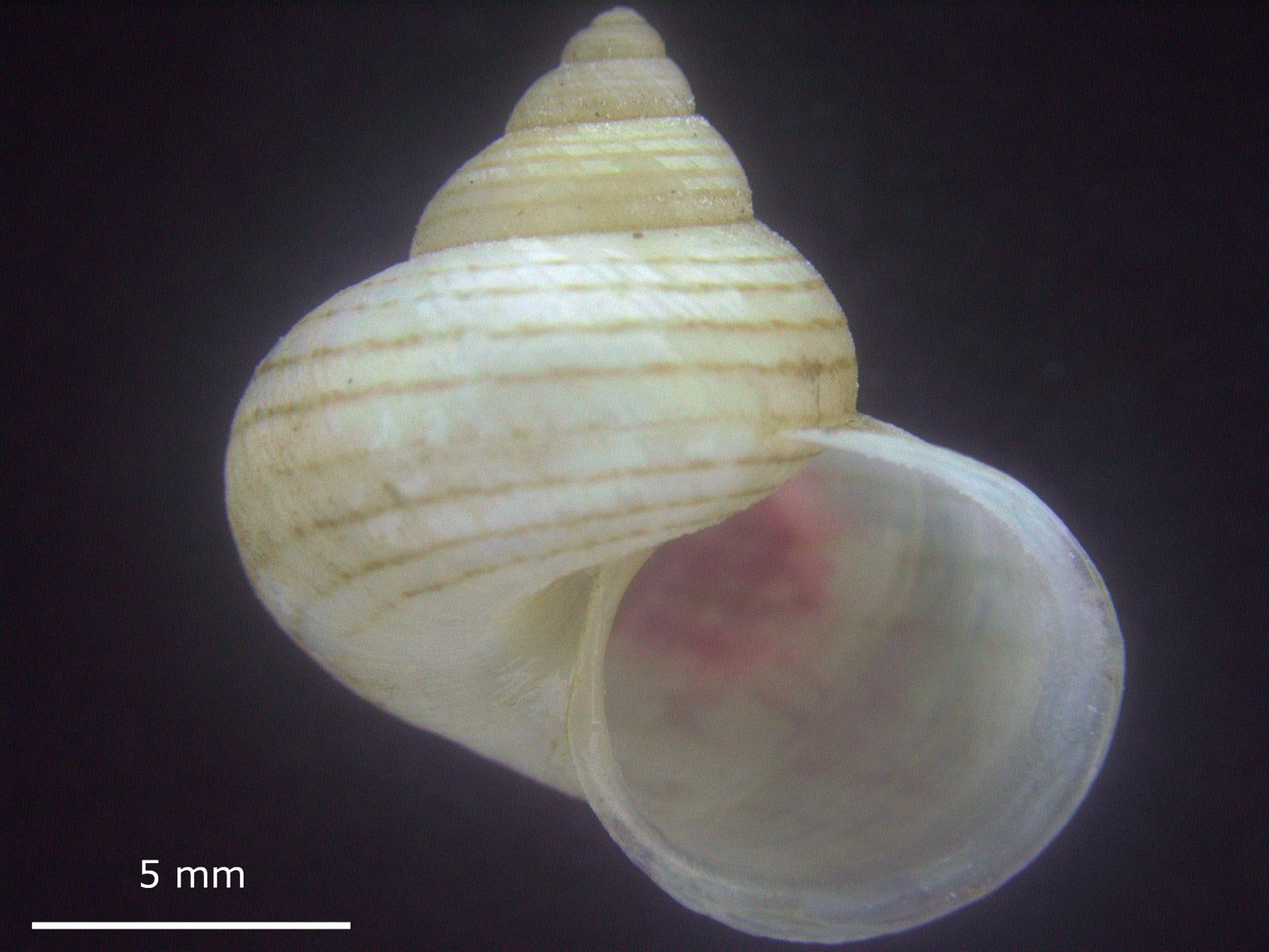

Supplement: File S4 [file peerj-10-13501-s004.zip › New Folder/8780.1.jpg]

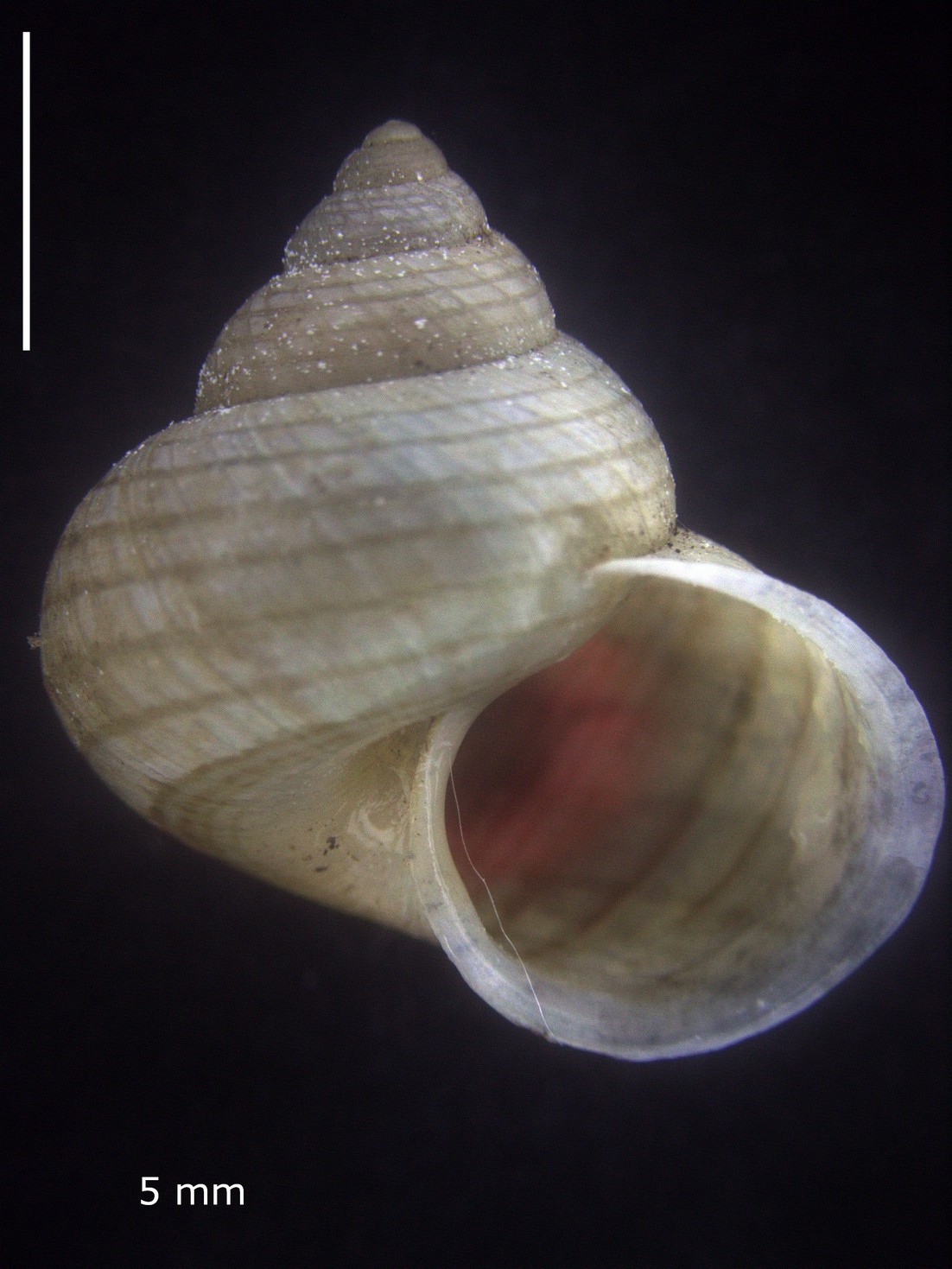

Supplement: File S4 [file peerj-10-13501-s004.zip › New Folder/8781.1.jpg]

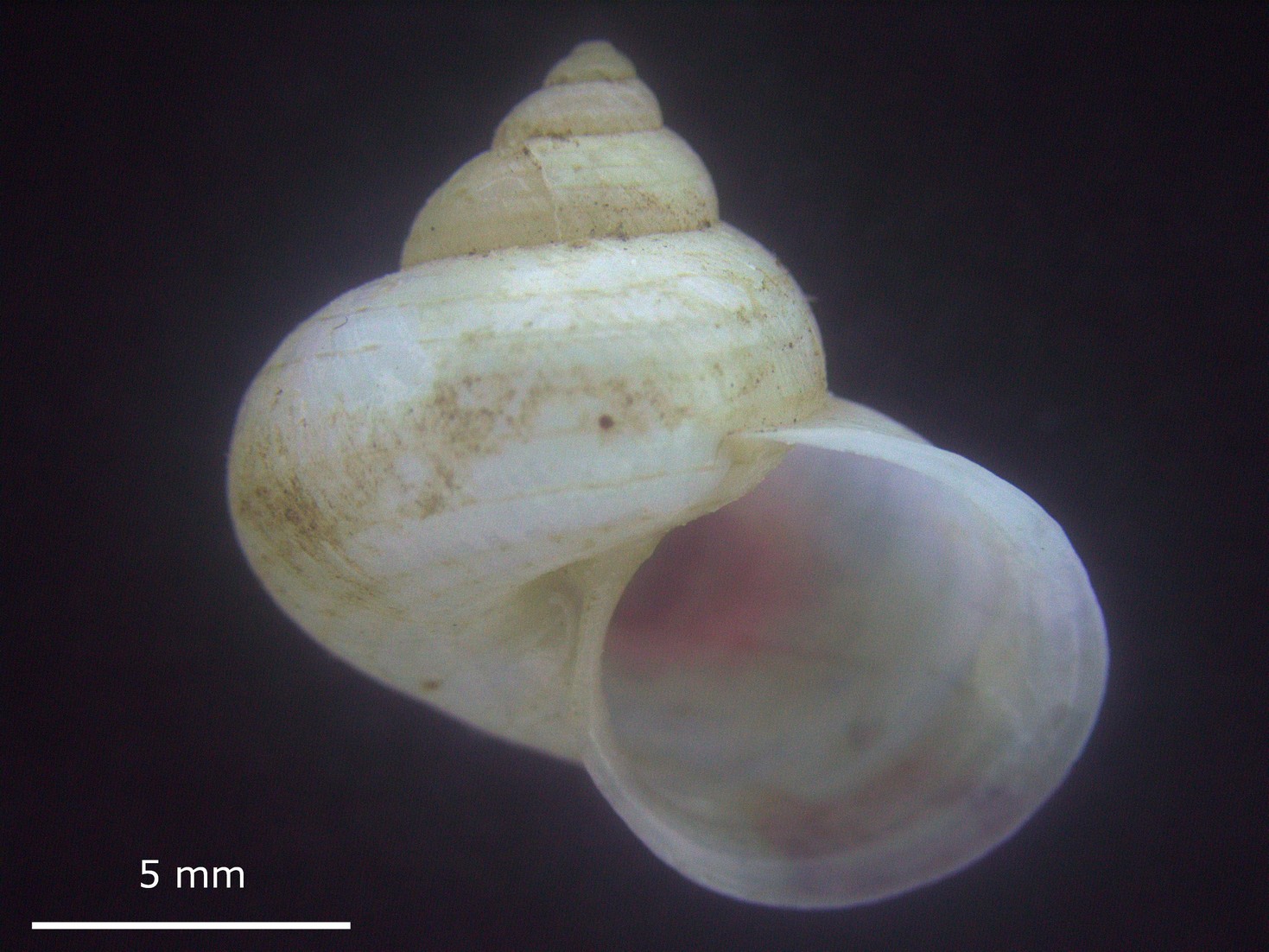

Supplement: File S4 [file peerj-10-13501-s004.zip › New Folder/8782.1.jpg]

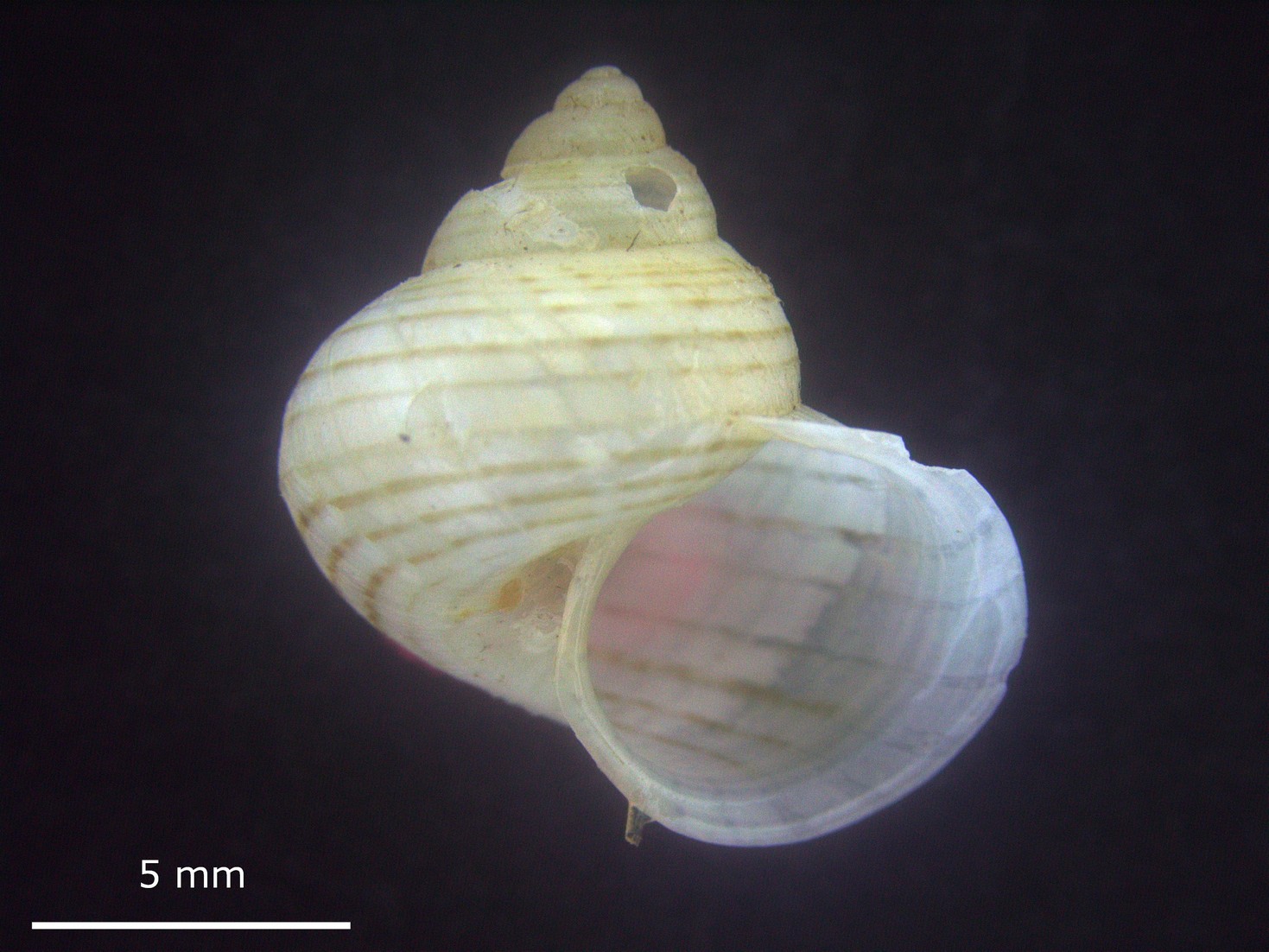

Supplement: File S4 [file peerj-10-13501-s004.zip › New Folder/8783.1.jpg]

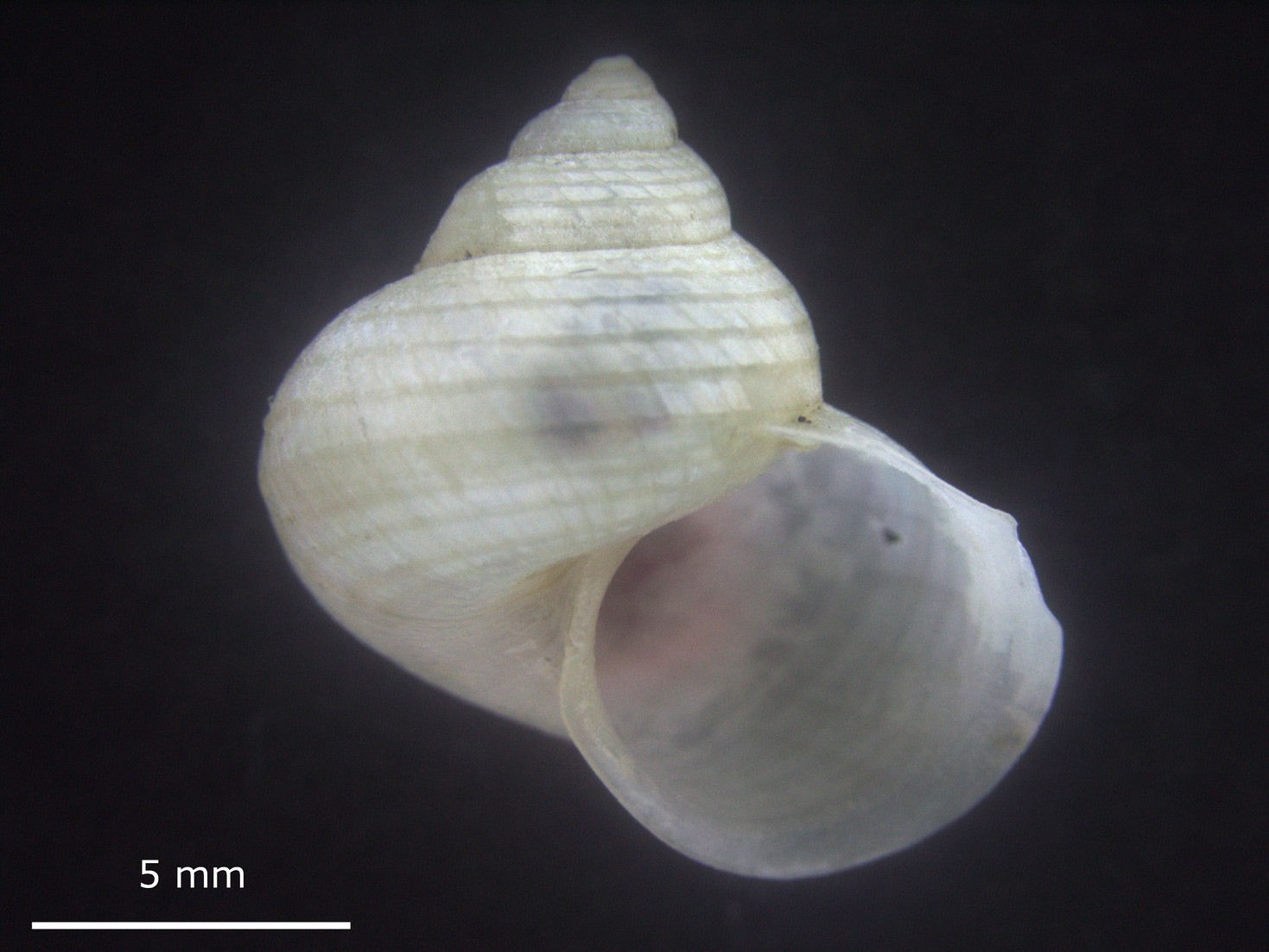

Supplement: File S4 [file peerj-10-13501-s004.zip › New Folder/8784.1.jpg]

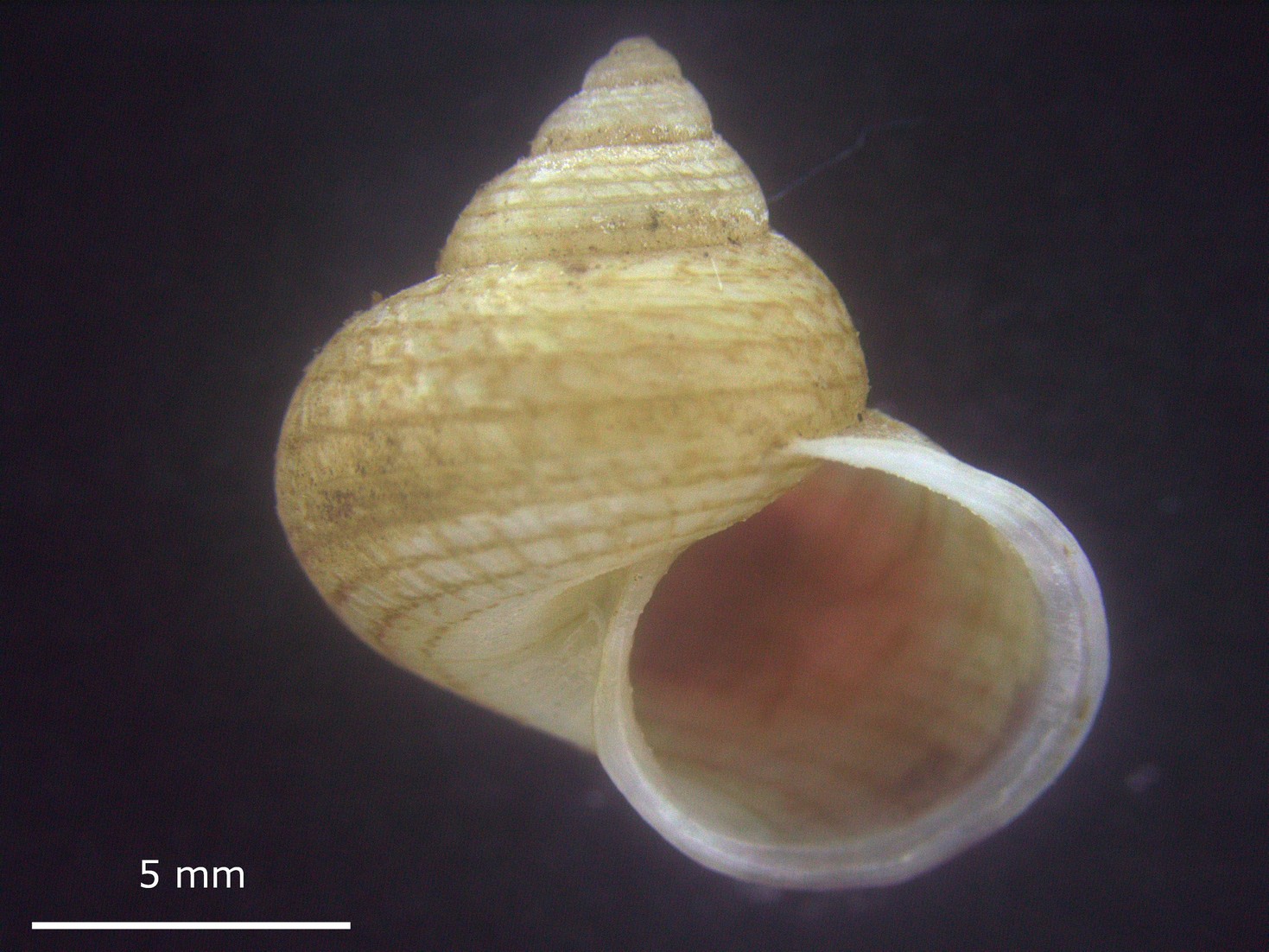

Supplement: File S4 [file peerj-10-13501-s004.zip › New Folder/8786.1.jpg]

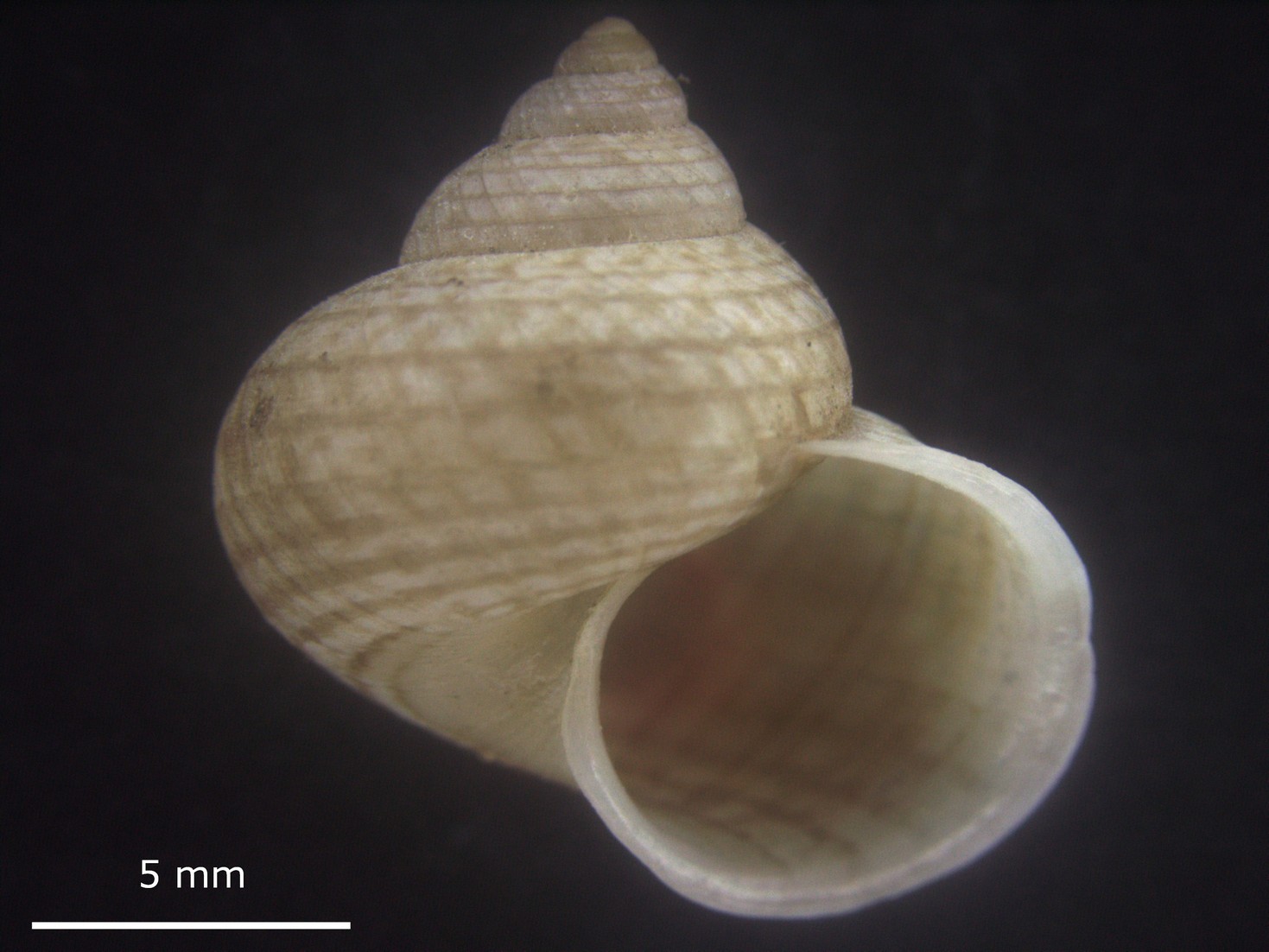

Supplement: File S4 [file peerj-10-13501-s004.zip › New Folder/8787.1.jpg]

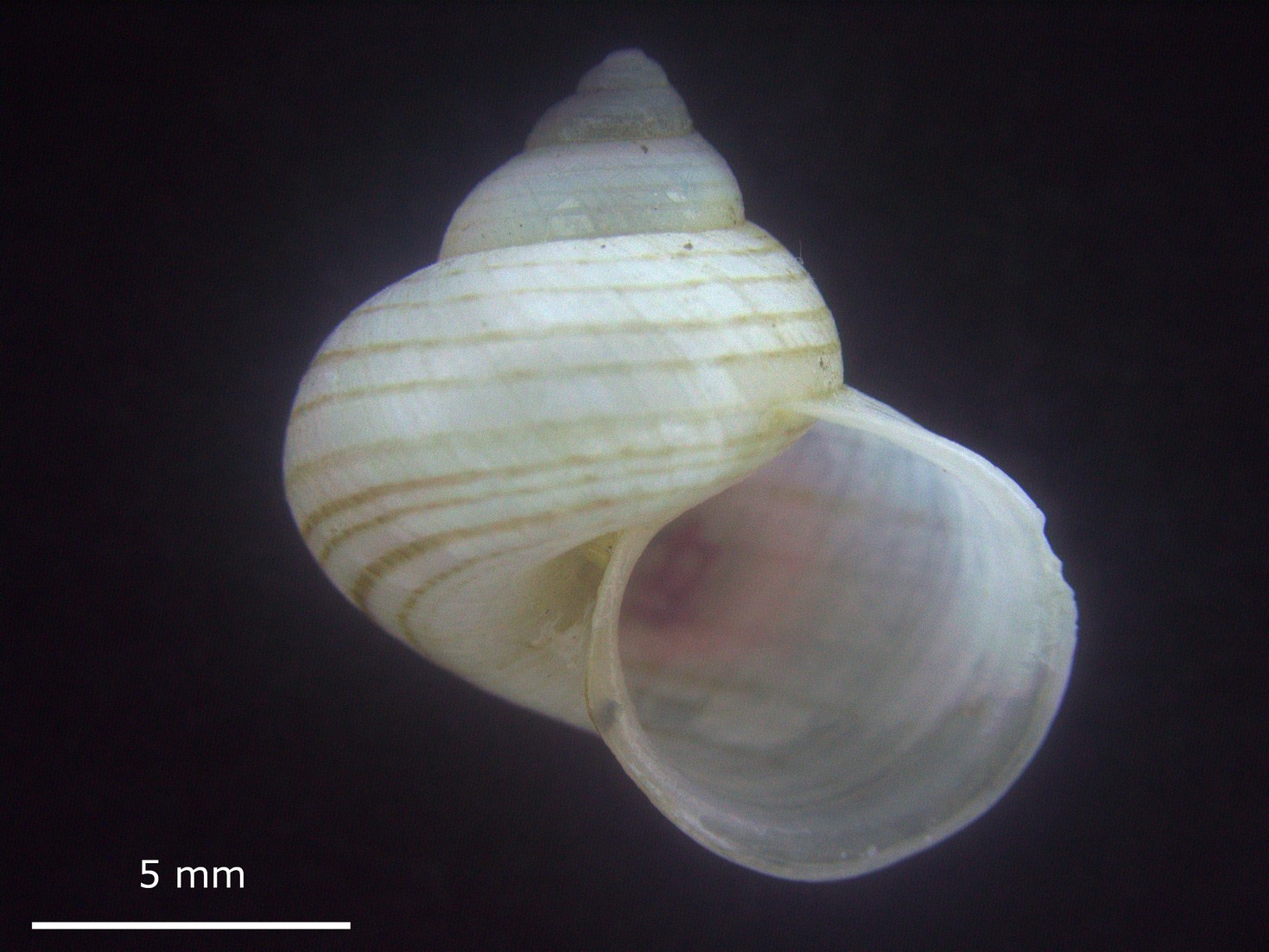

Supplement: File S4 [file peerj-10-13501-s004.zip › New Folder/8788.1.jpg]

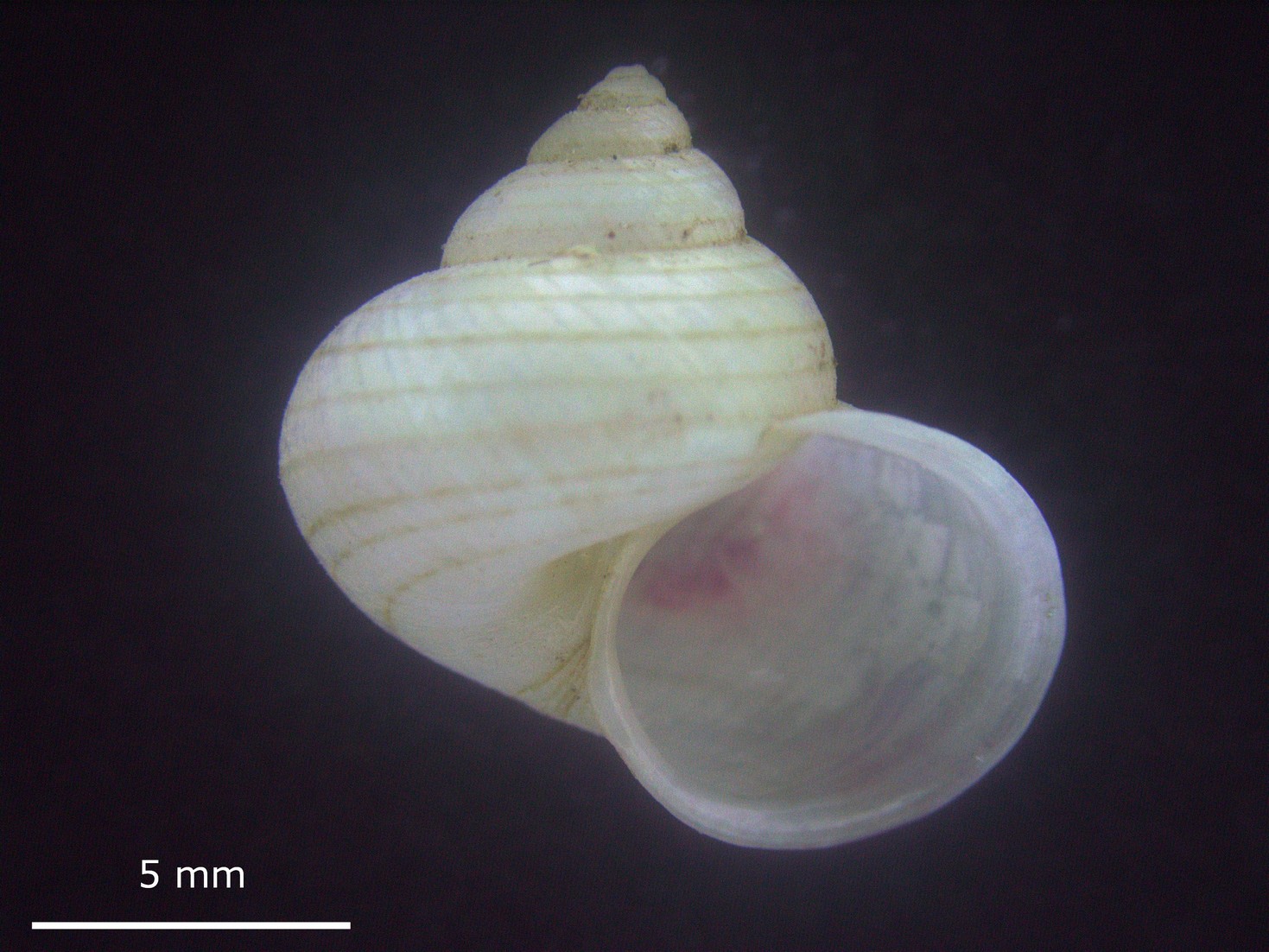

Supplement: File S4 [file peerj-10-13501-s004.zip › New Folder/8789.1.jpg]

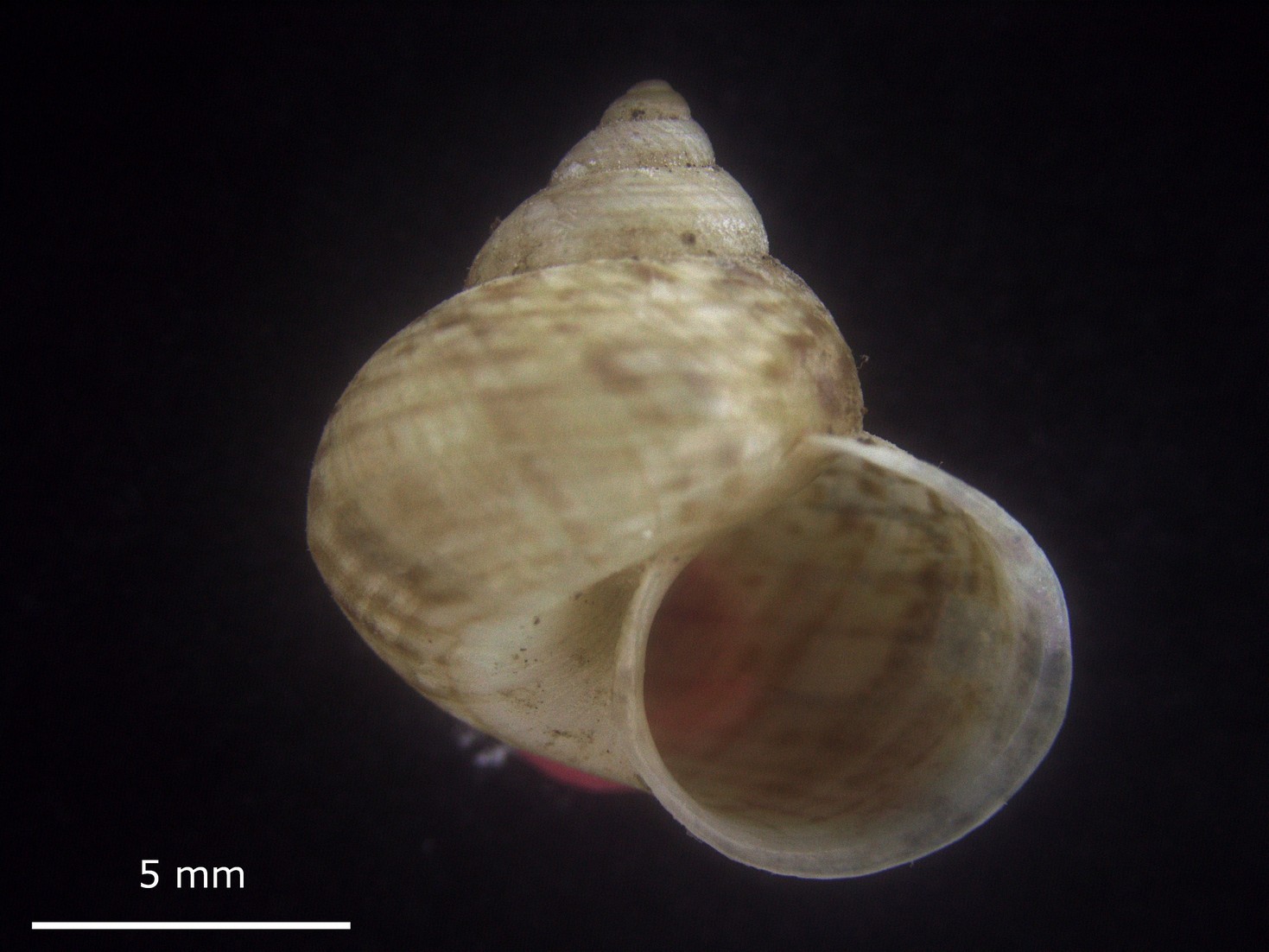

Supplement: File S4 [file peerj-10-13501-s004.zip › New Folder/8790.1.jpg]

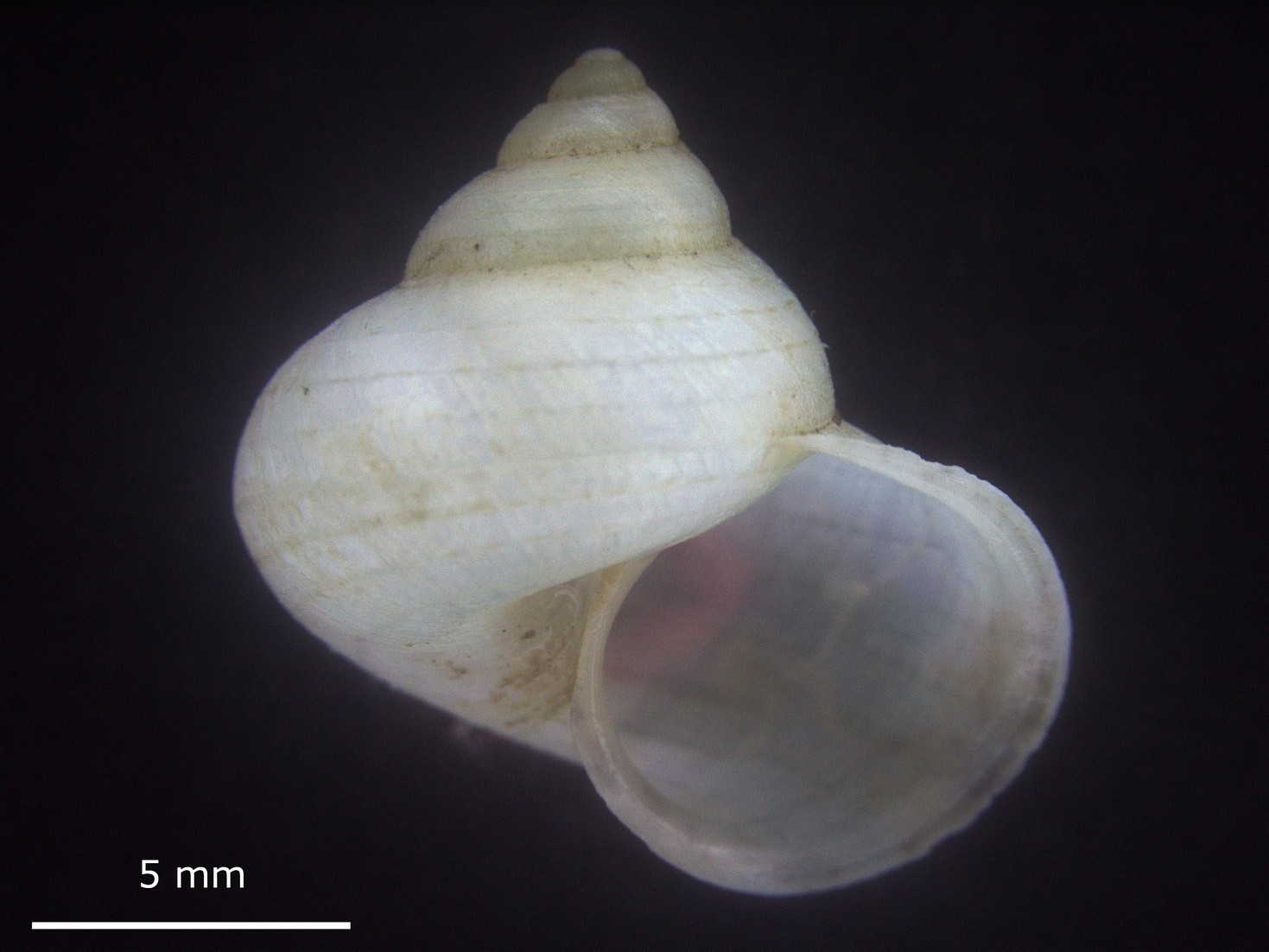

Supplement: File S4 [file peerj-10-13501-s004.zip › New Folder/8791.1.jpg]

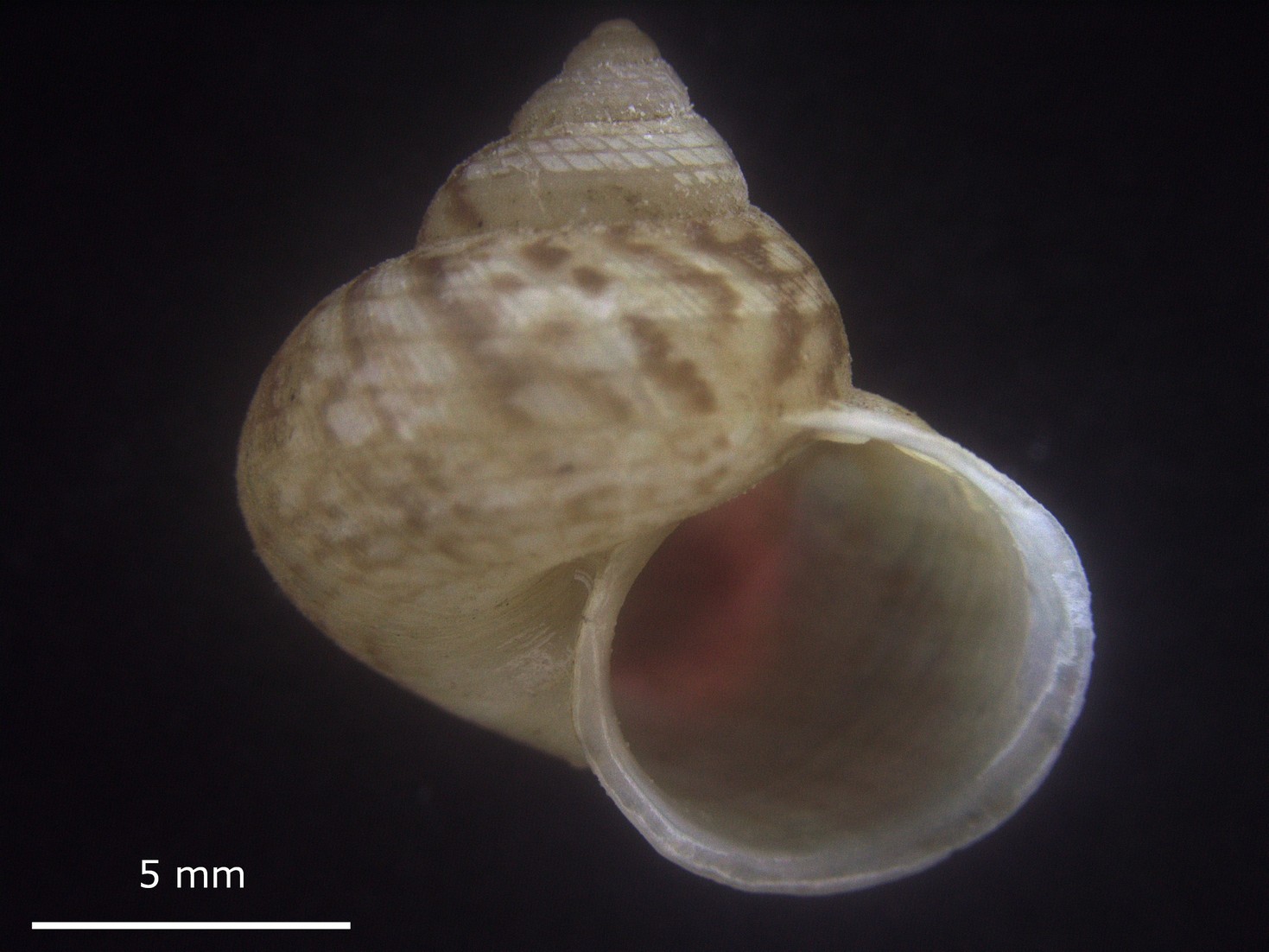

Supplement: File S4 [file peerj-10-13501-s004.zip › New Folder/8792.1.jpg]

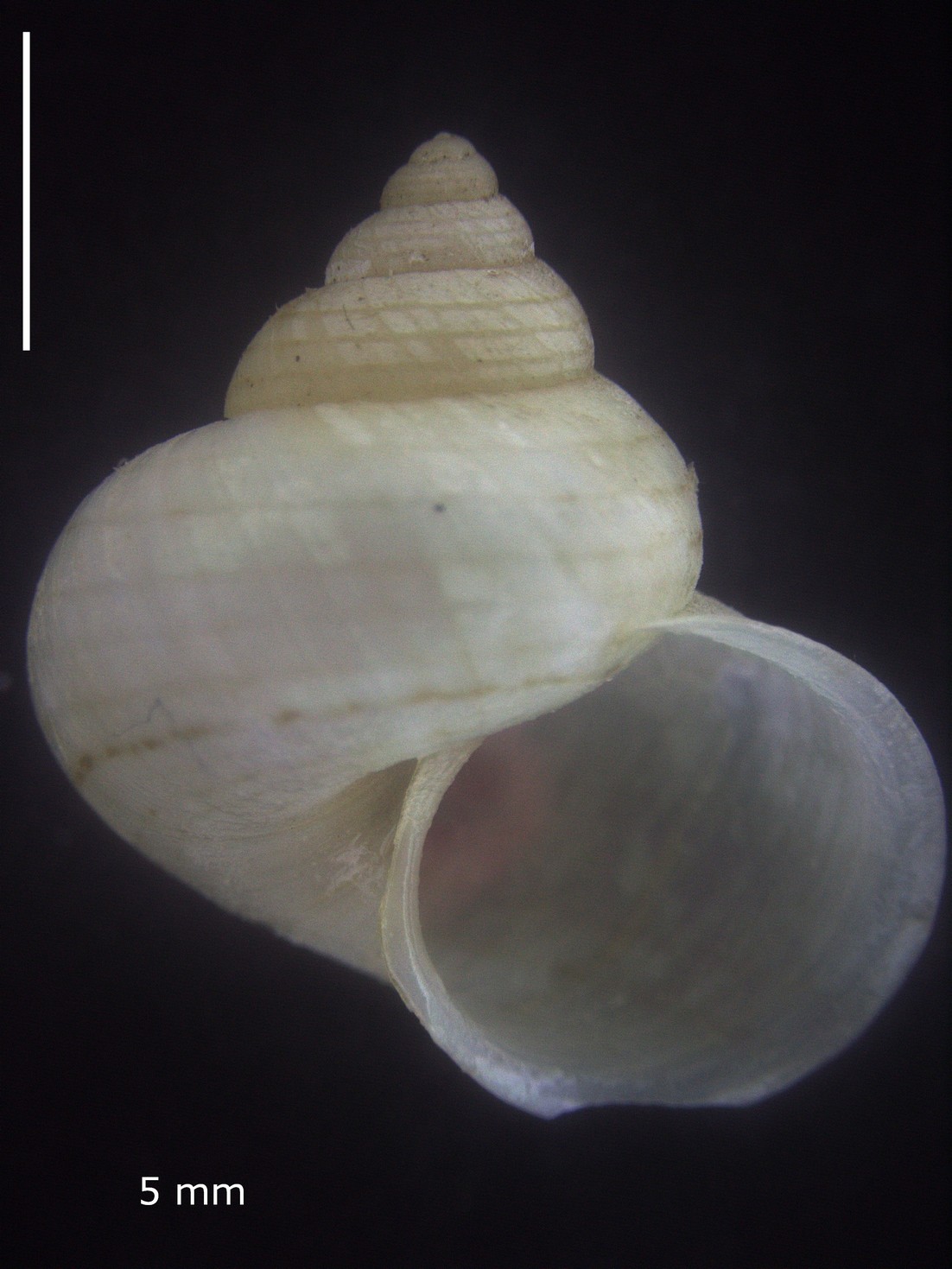

Supplement: File S4 [file peerj-10-13501-s004.zip › New Folder/8793.1.jpg]

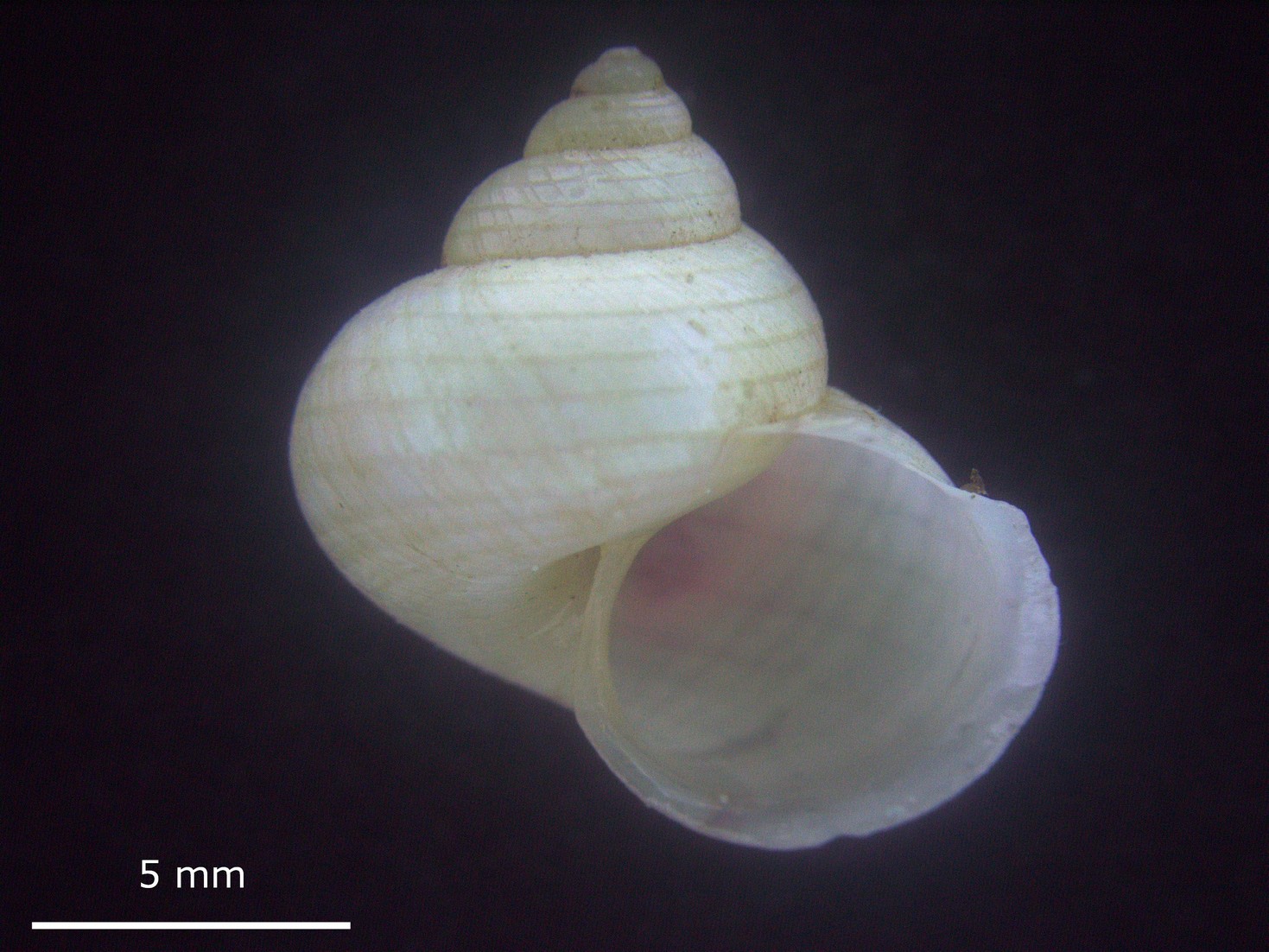

Supplement: File S4 [file peerj-10-13501-s004.zip › New Folder/8794.1.jpg]

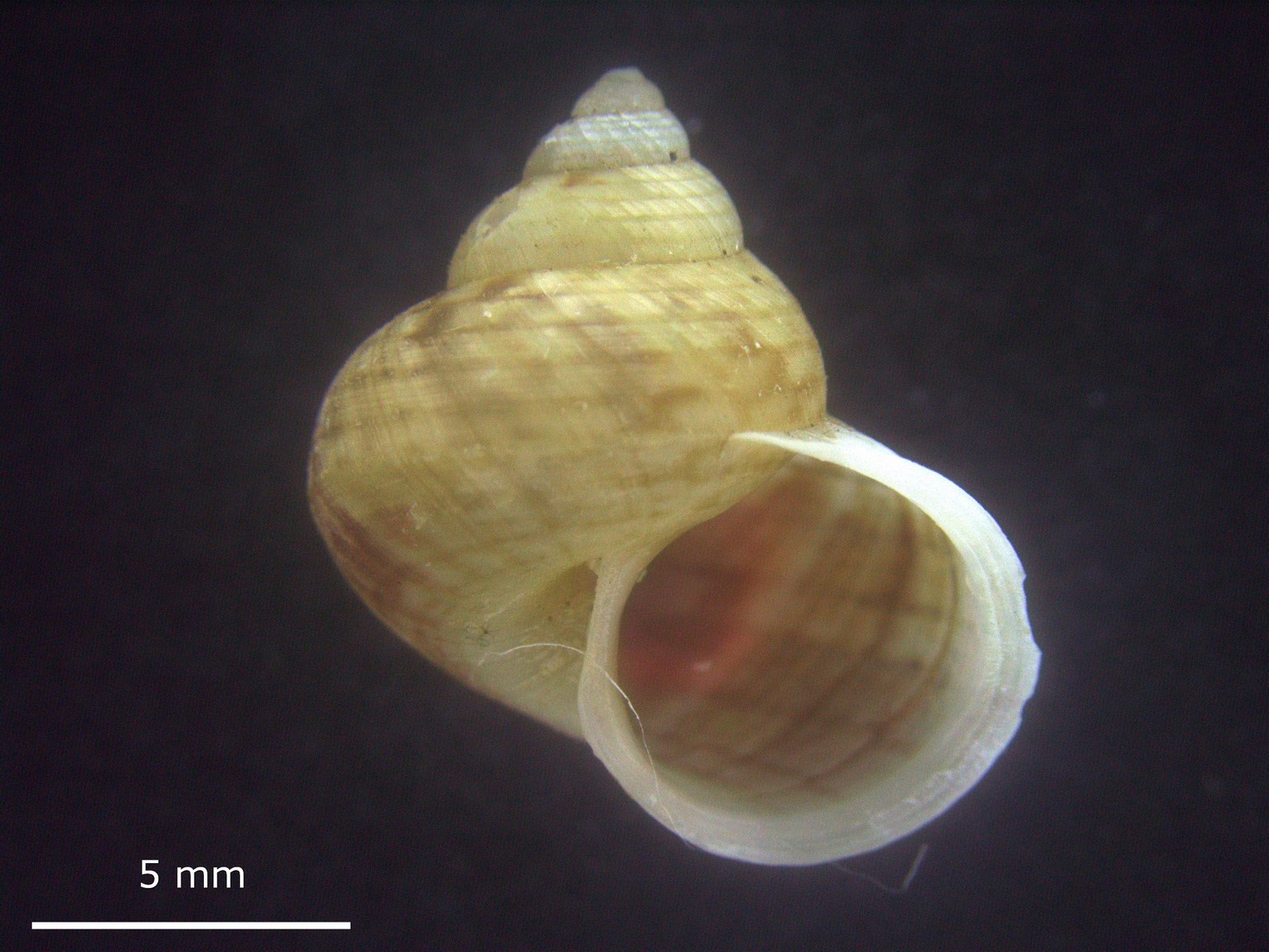

Supplement: File S4 [file peerj-10-13501-s004.zip › New Folder/8797.1.jpg]

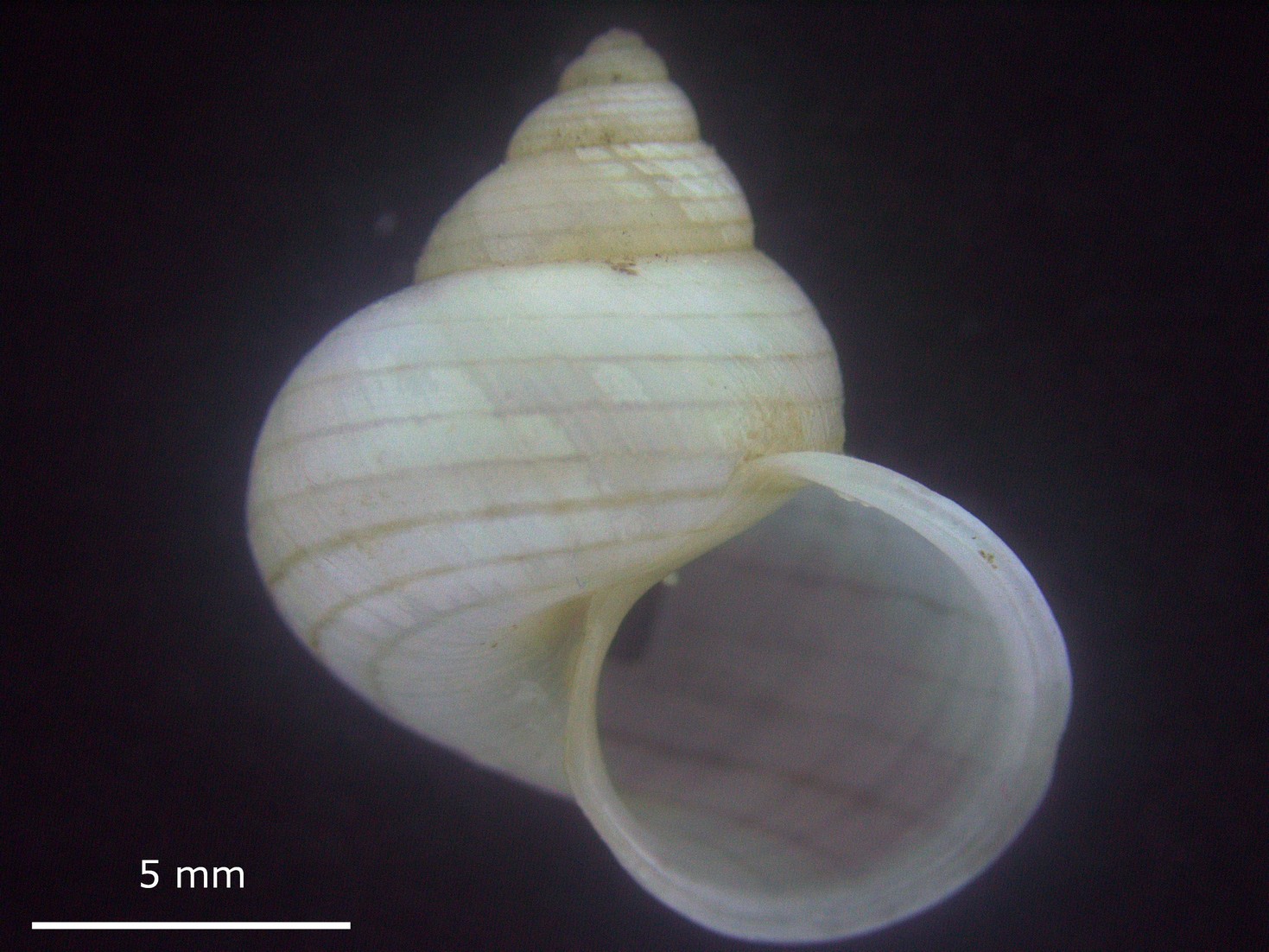

Supplement: File S4 [file peerj-10-13501-s004.zip › New Folder/8798.1.jpg]

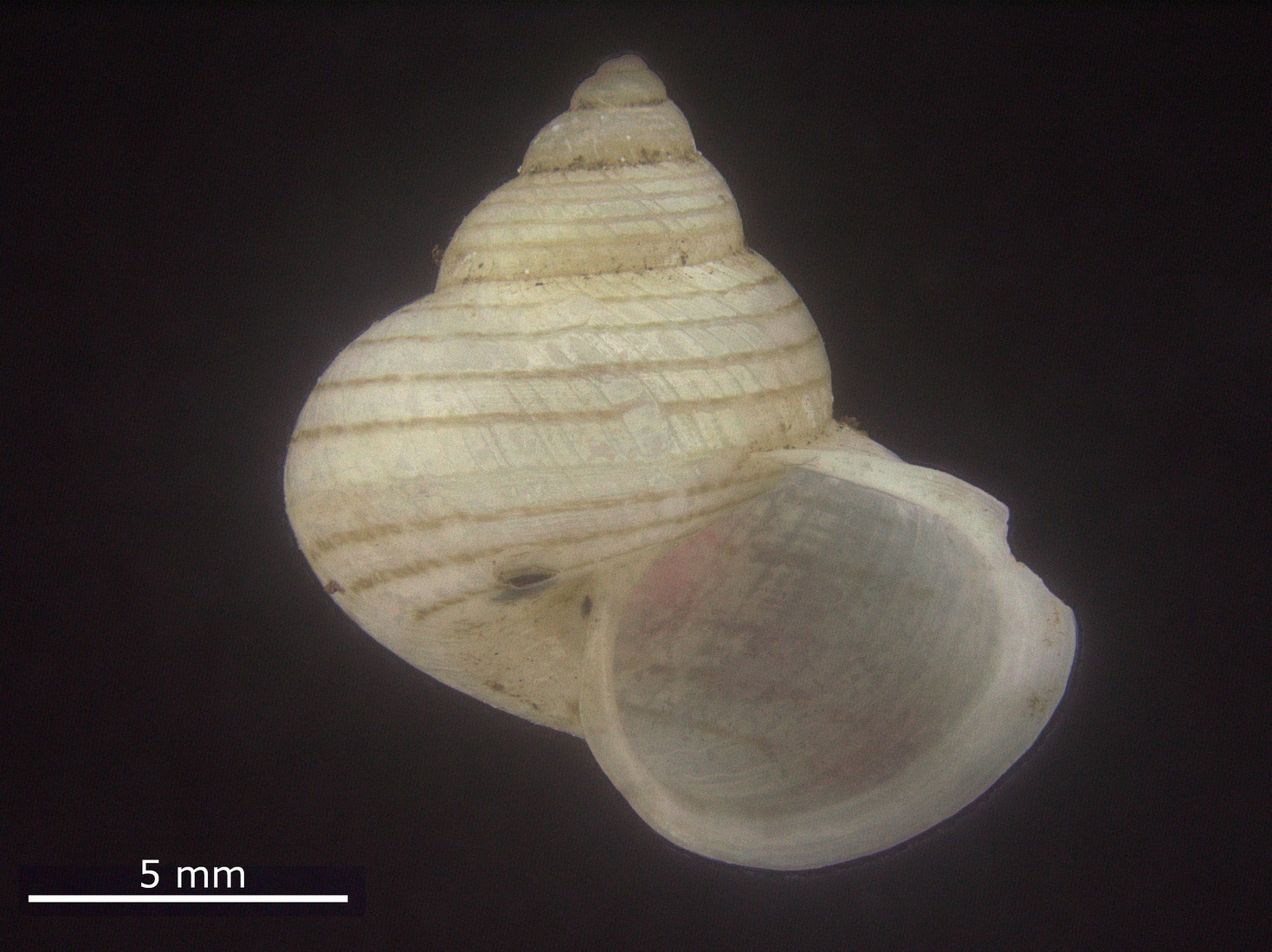

Supplement: File S4 [file peerj-10-13501-s004.zip › New Folder/8799.1.jpg]

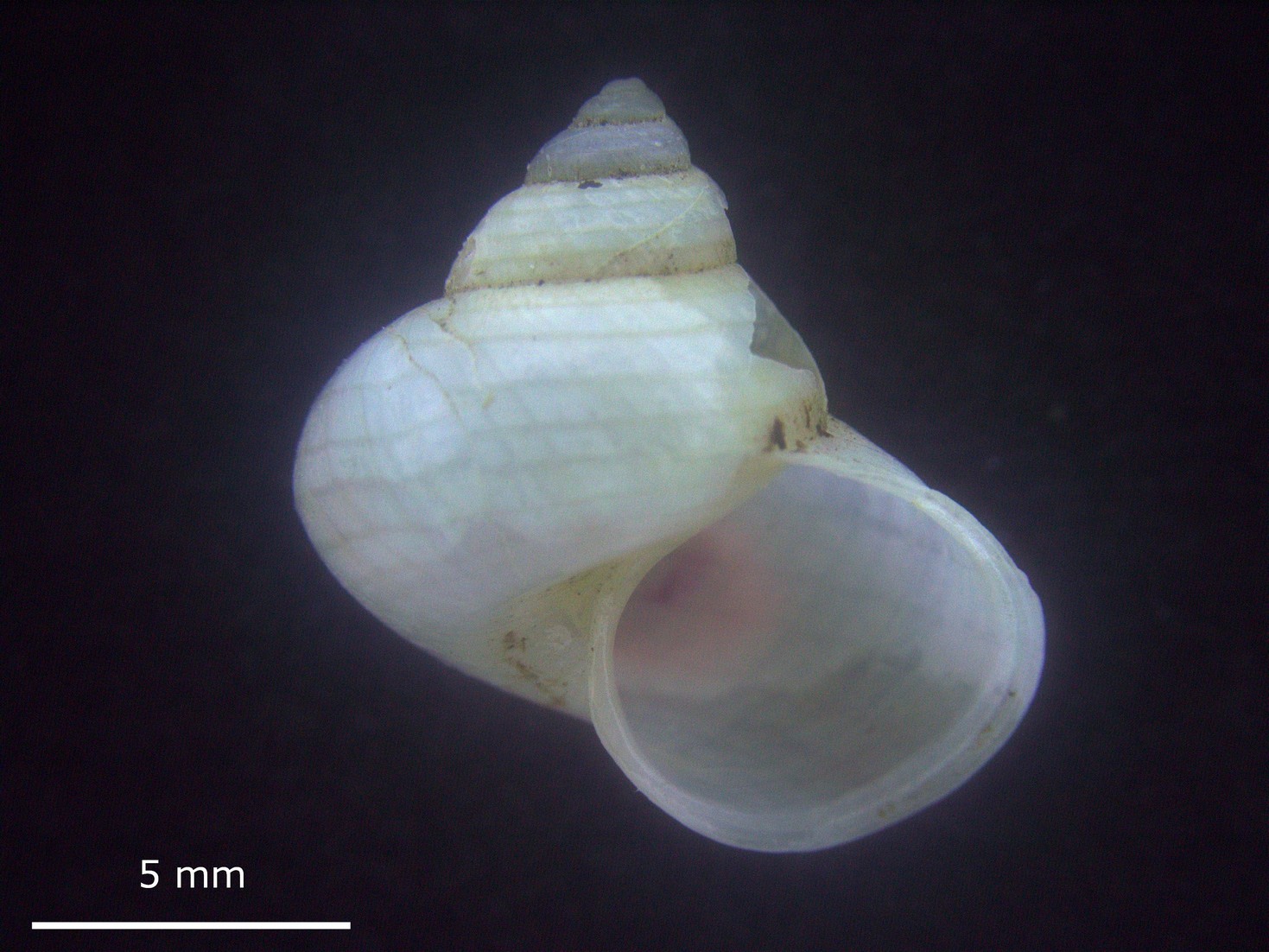

Supplement: File S4 [file peerj-10-13501-s004.zip › New Folder/8800.1.jpg]

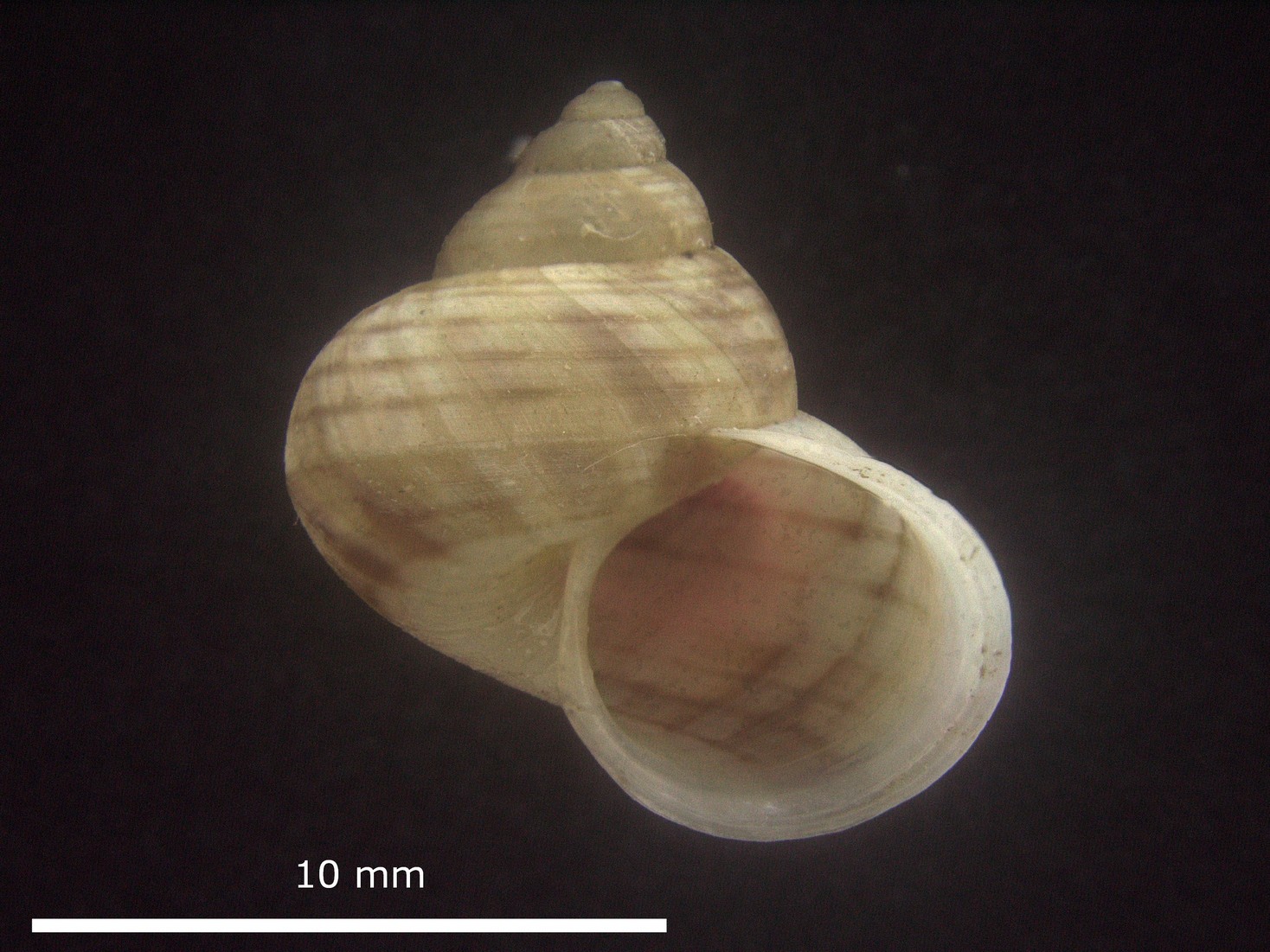

Supplement: File S4 [file peerj-10-13501-s004.zip › New Folder/8801.1.jpg]

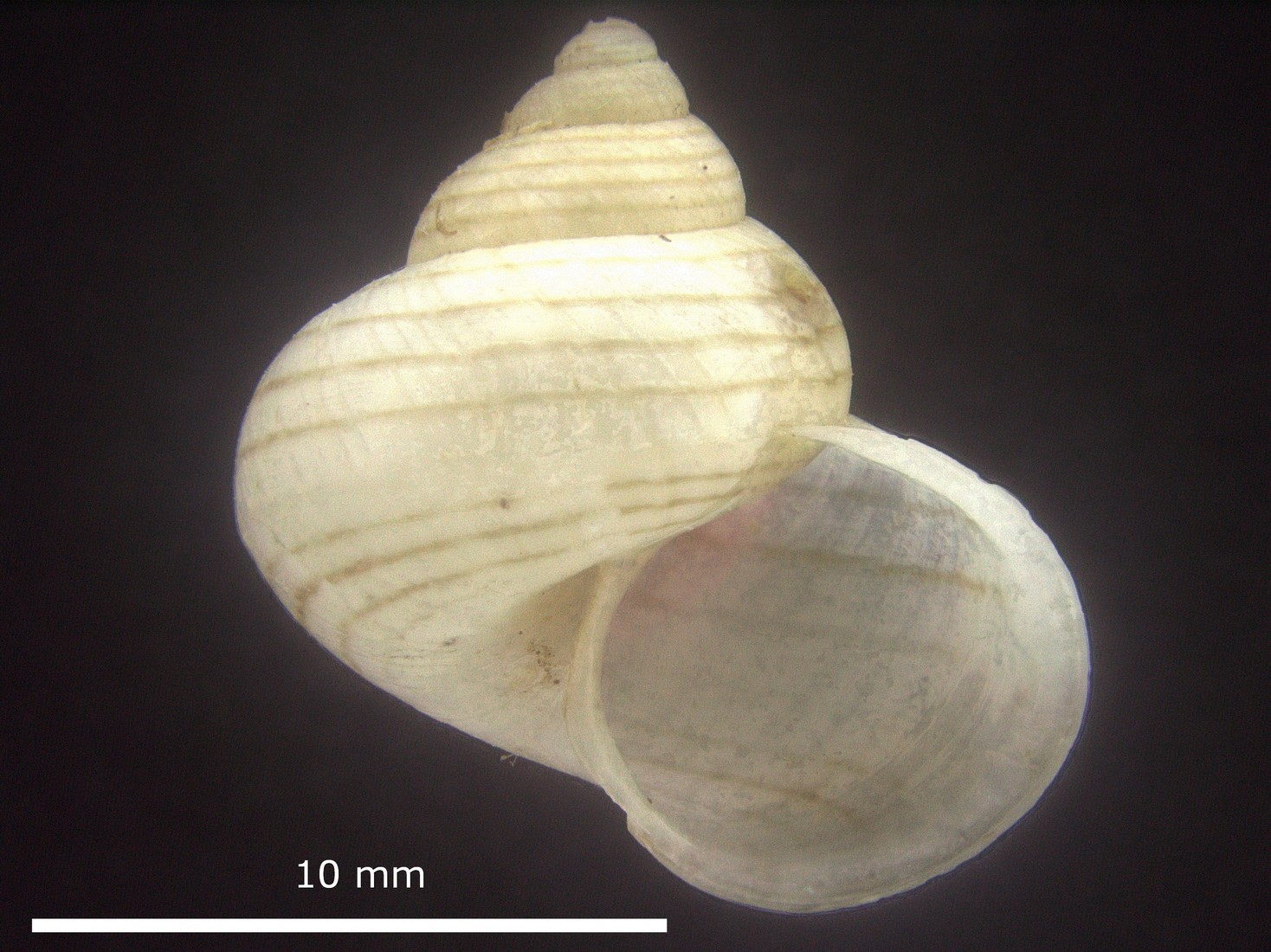

Supplement: File S4 [file peerj-10-13501-s004.zip › New Folder/8802.1.jpg]

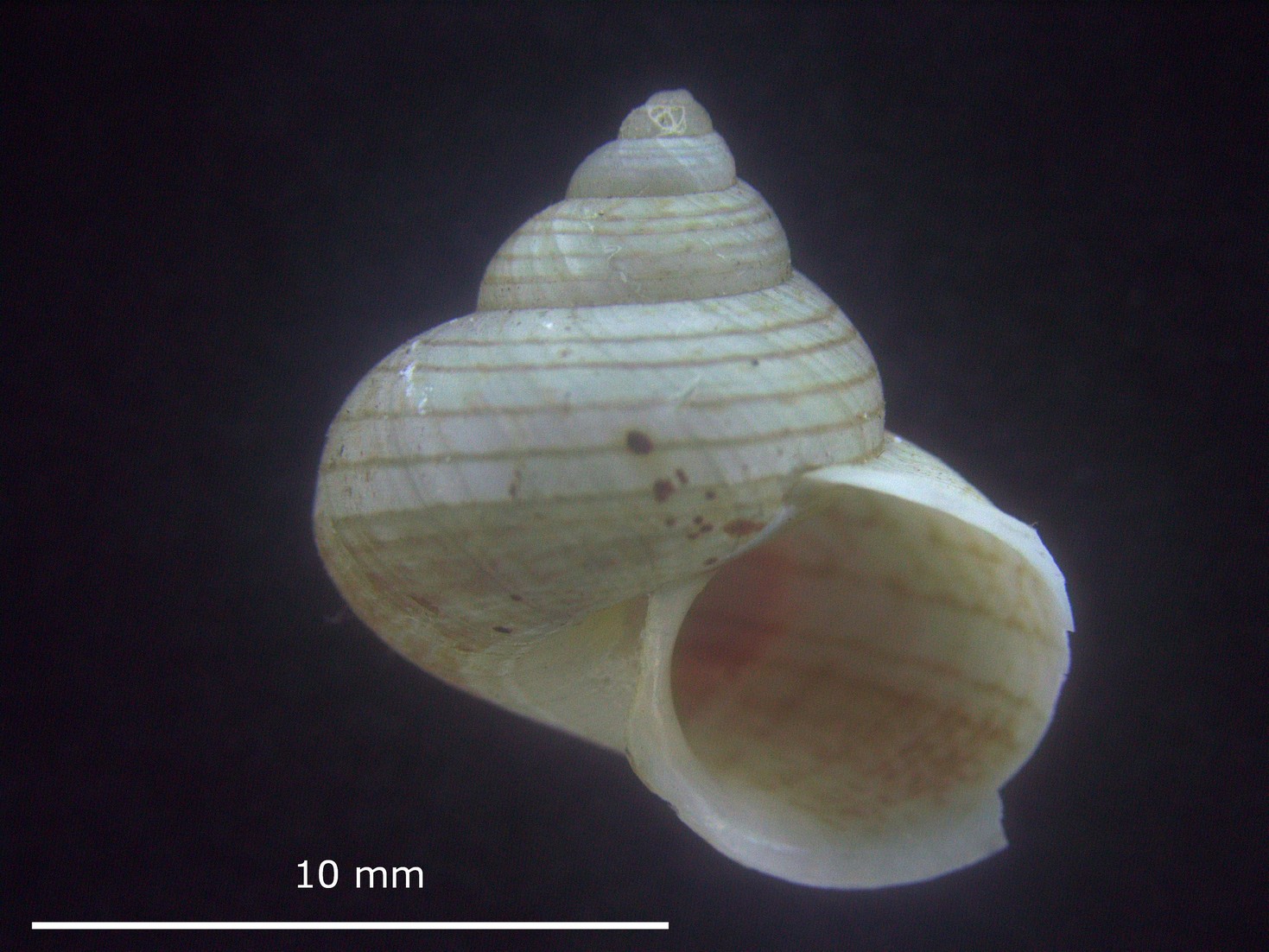

Supplement: File S4 [file peerj-10-13501-s004.zip › New Folder/8805.1.jpg]

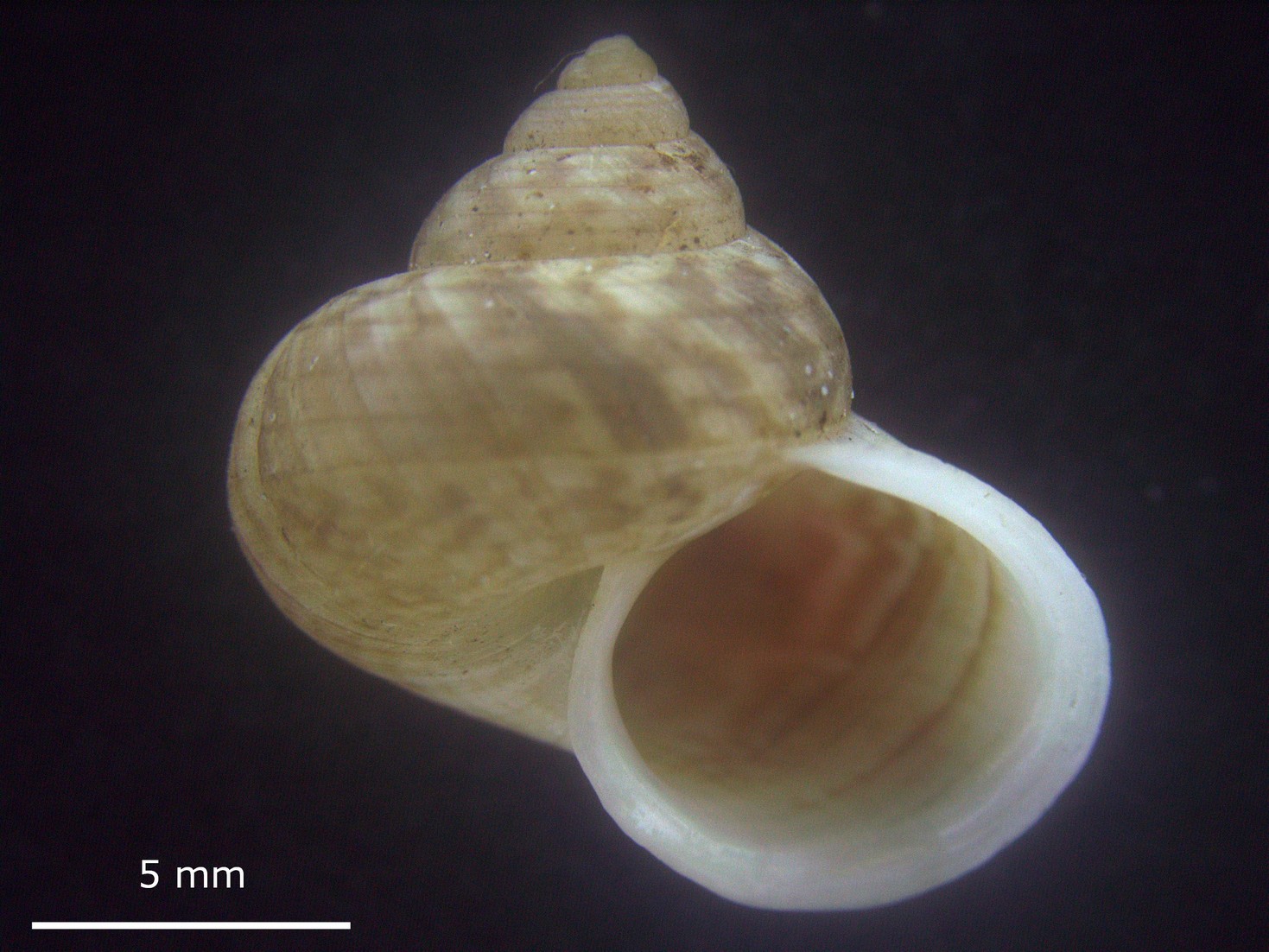

Supplement: File S4 [file peerj-10-13501-s004.zip › New Folder/8806.1.jpg]

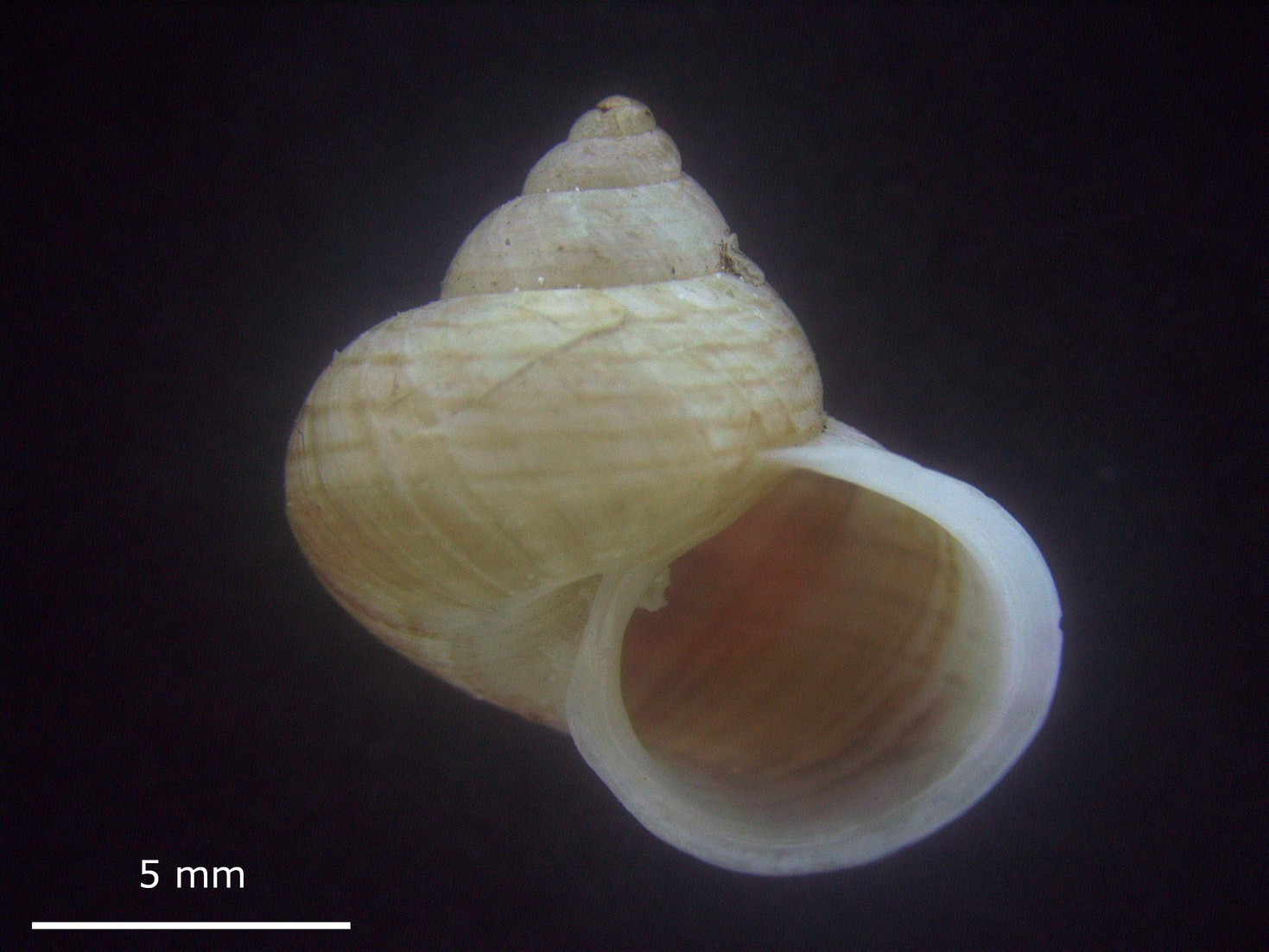

Supplement: File S4 [file peerj-10-13501-s004.zip › New Folder/8807.1.jpg]

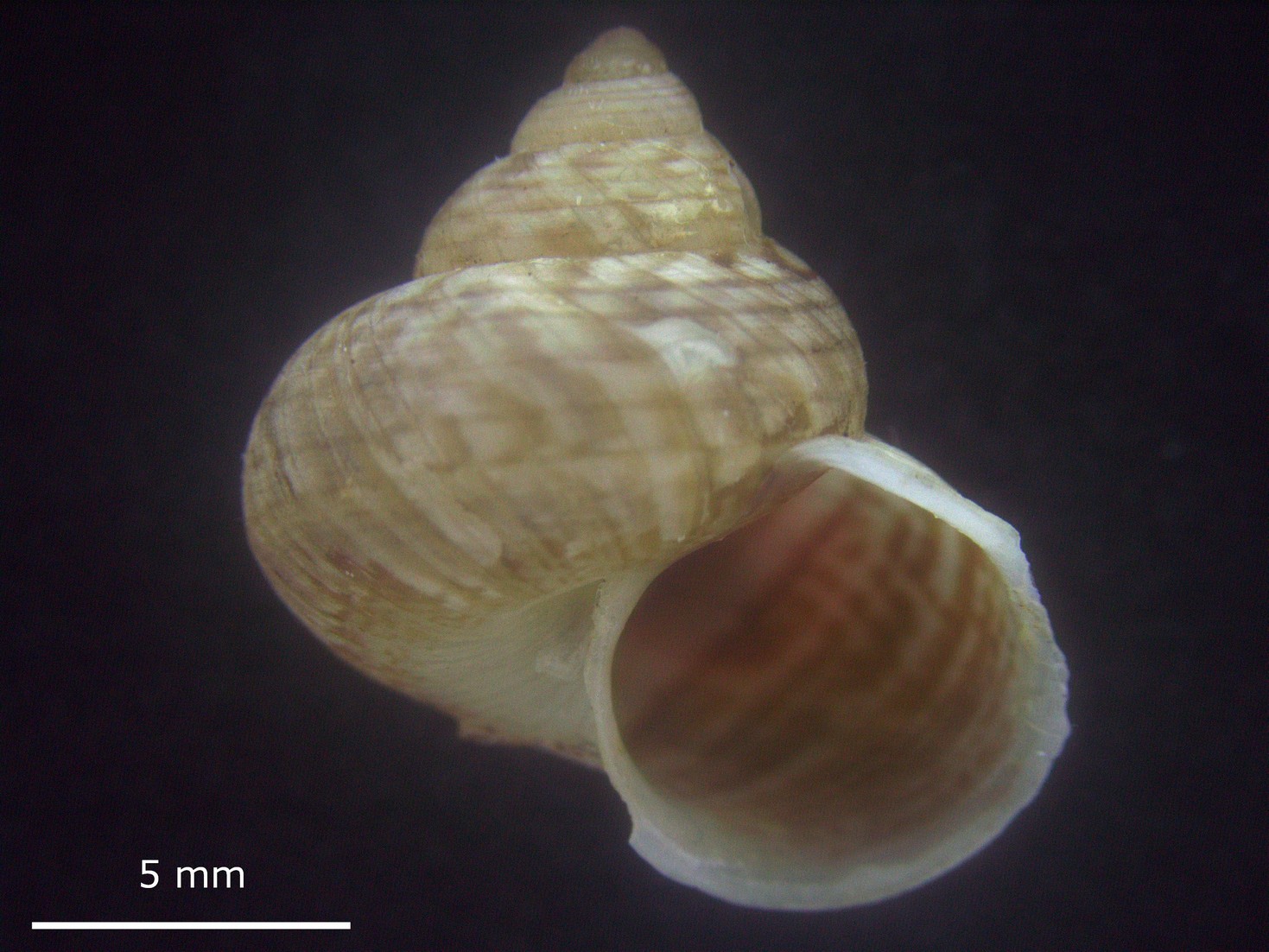

Supplement: File S4 [file peerj-10-13501-s004.zip › New Folder/8808.1.jpg]

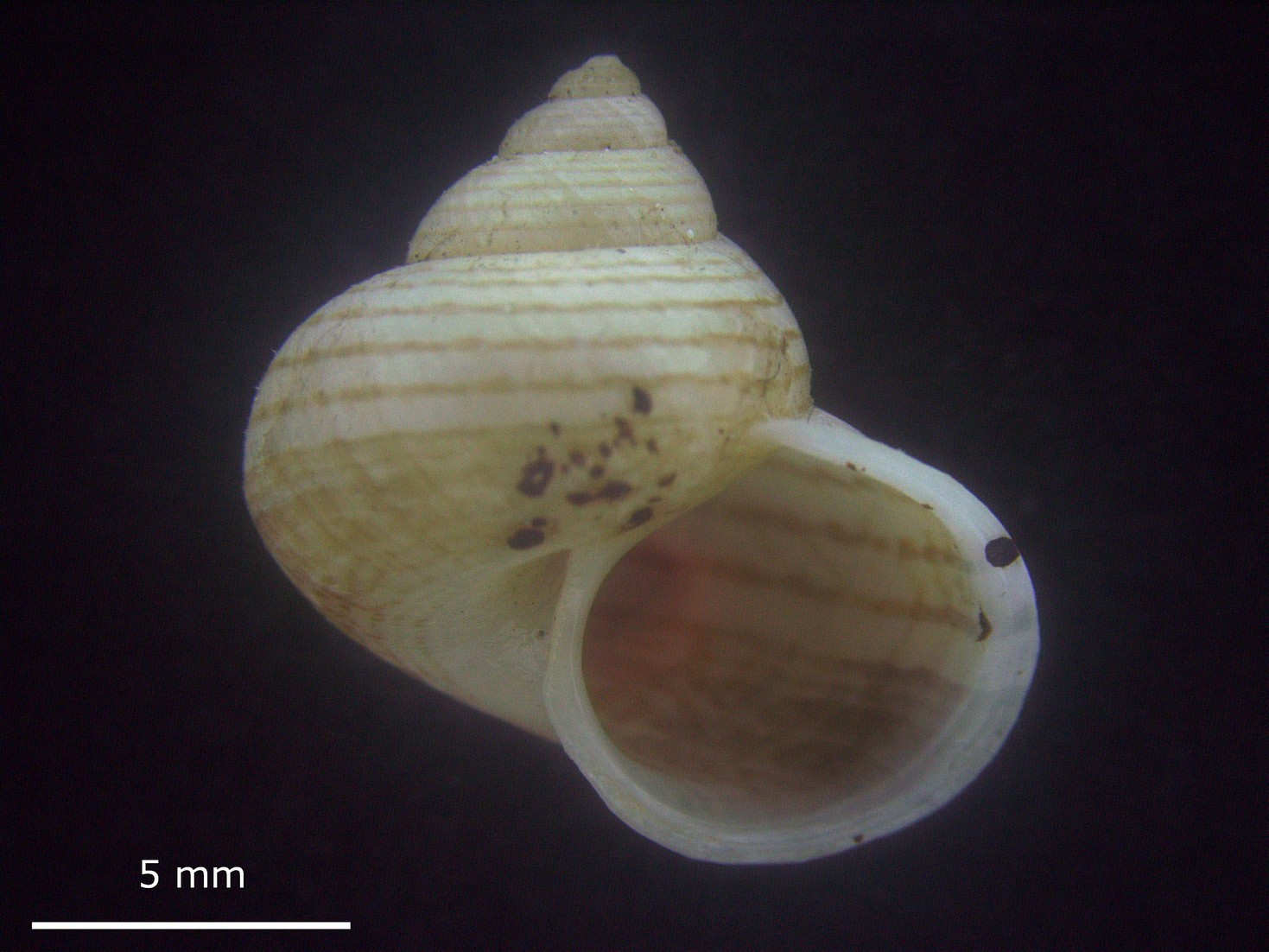

Supplement: File S4 [file peerj-10-13501-s004.zip › New Folder/8809.1.jpg]

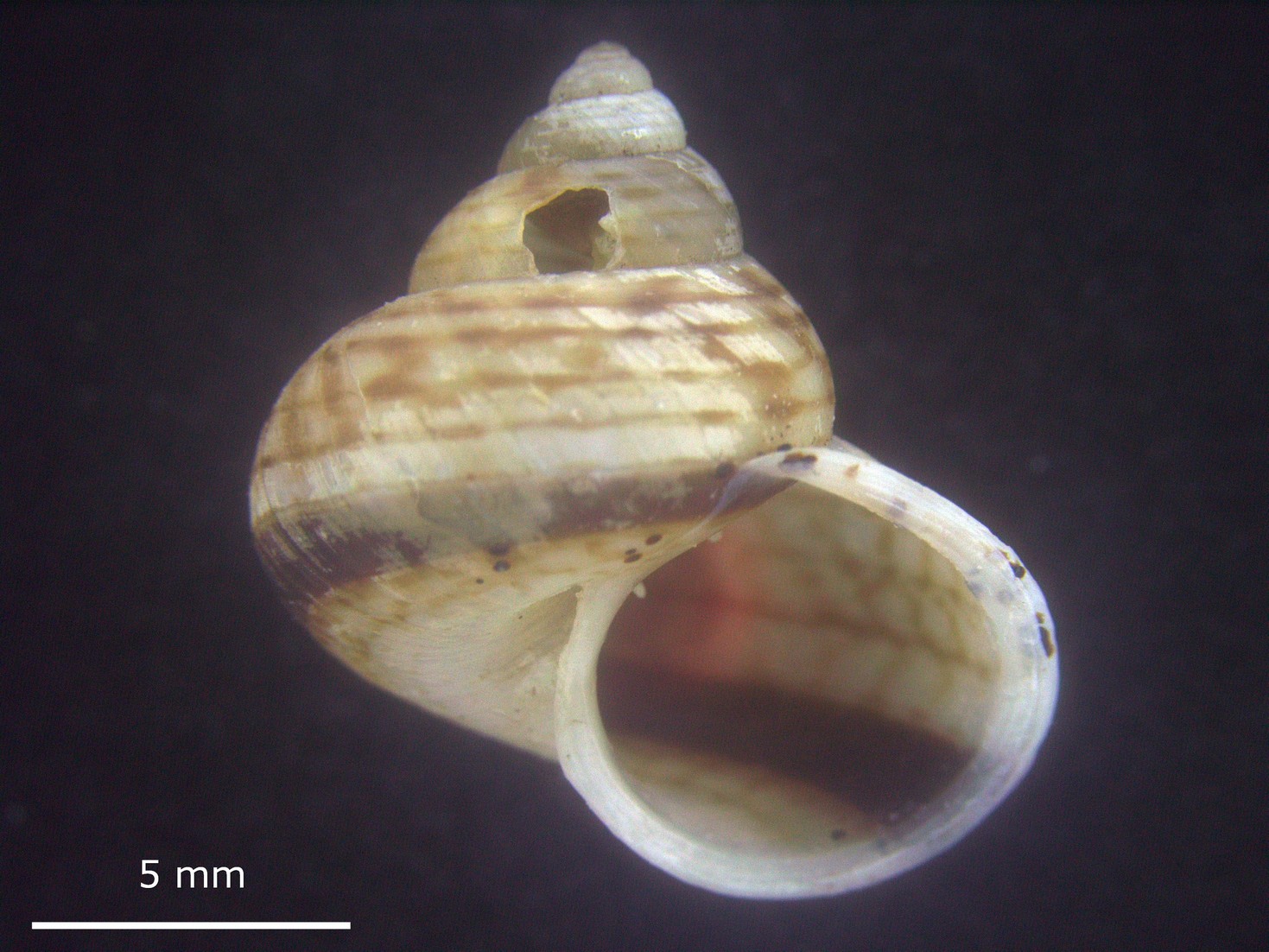

Supplement: File S4 [file peerj-10-13501-s004.zip › New Folder/8813.1.jpg]

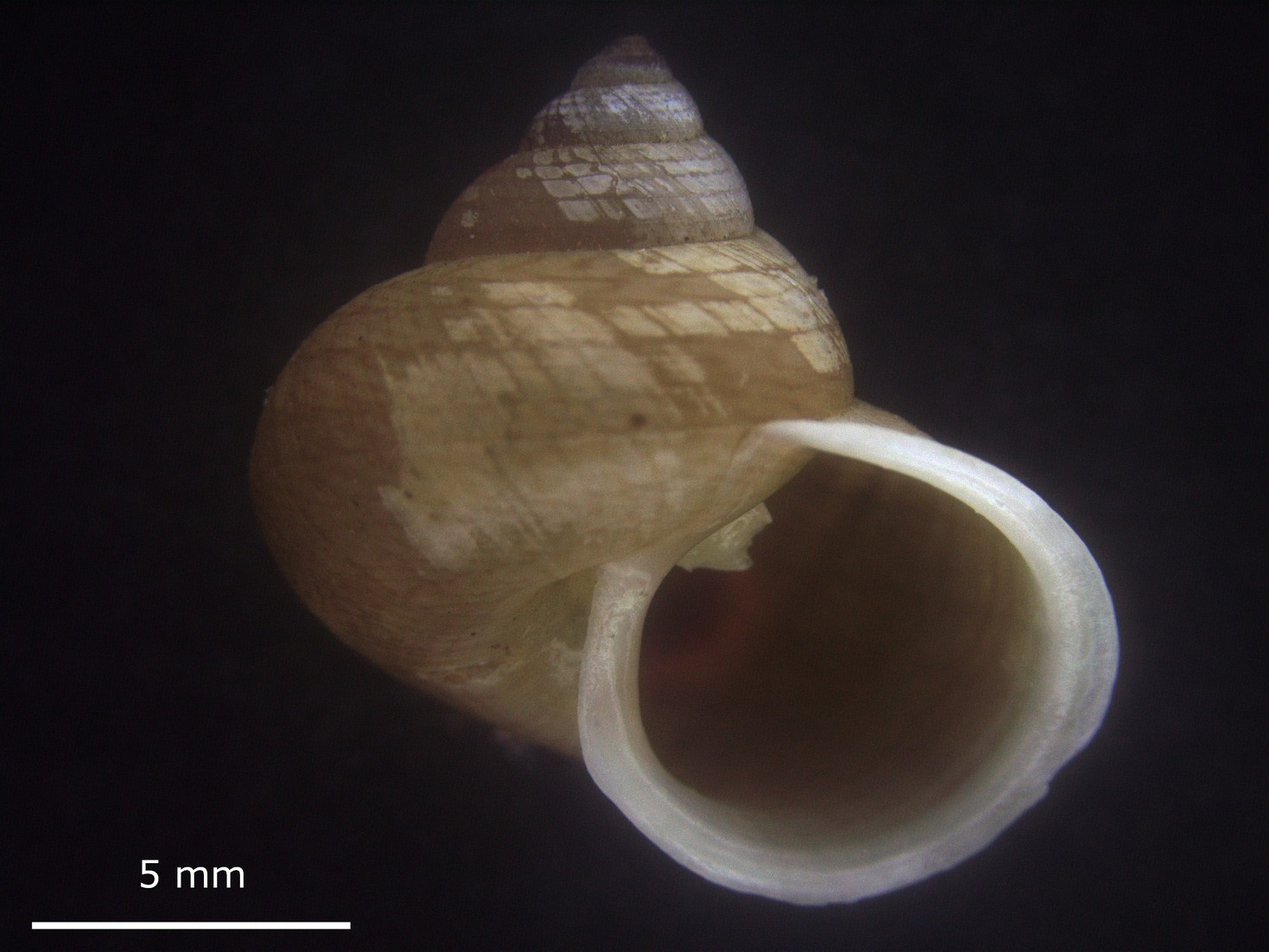

Supplement: File S4 [file peerj-10-13501-s004.zip › New Folder/8816.1.jpg]

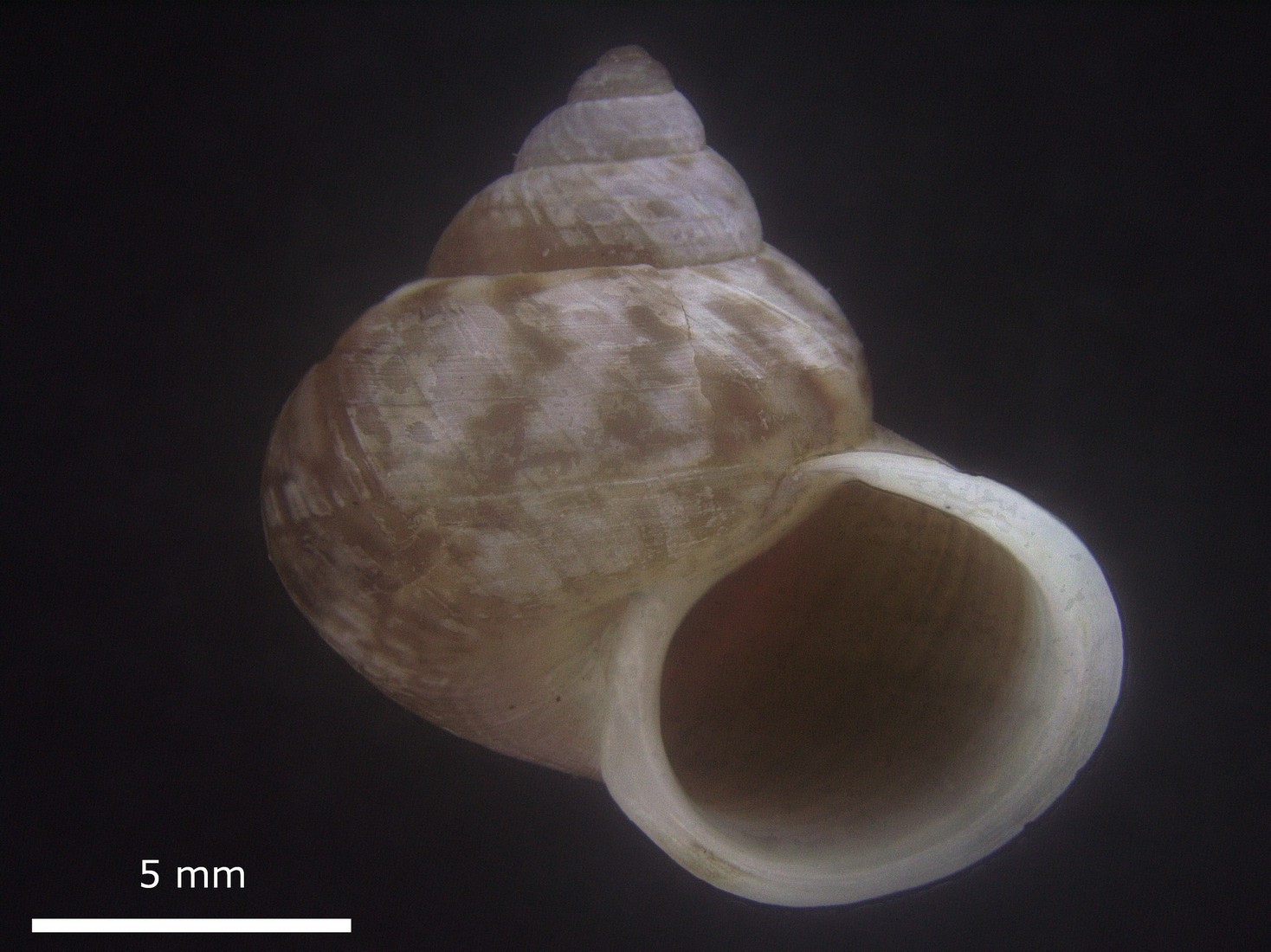

Supplement: File S4 [file peerj-10-13501-s004.zip › New Folder/8817.1.jpg]

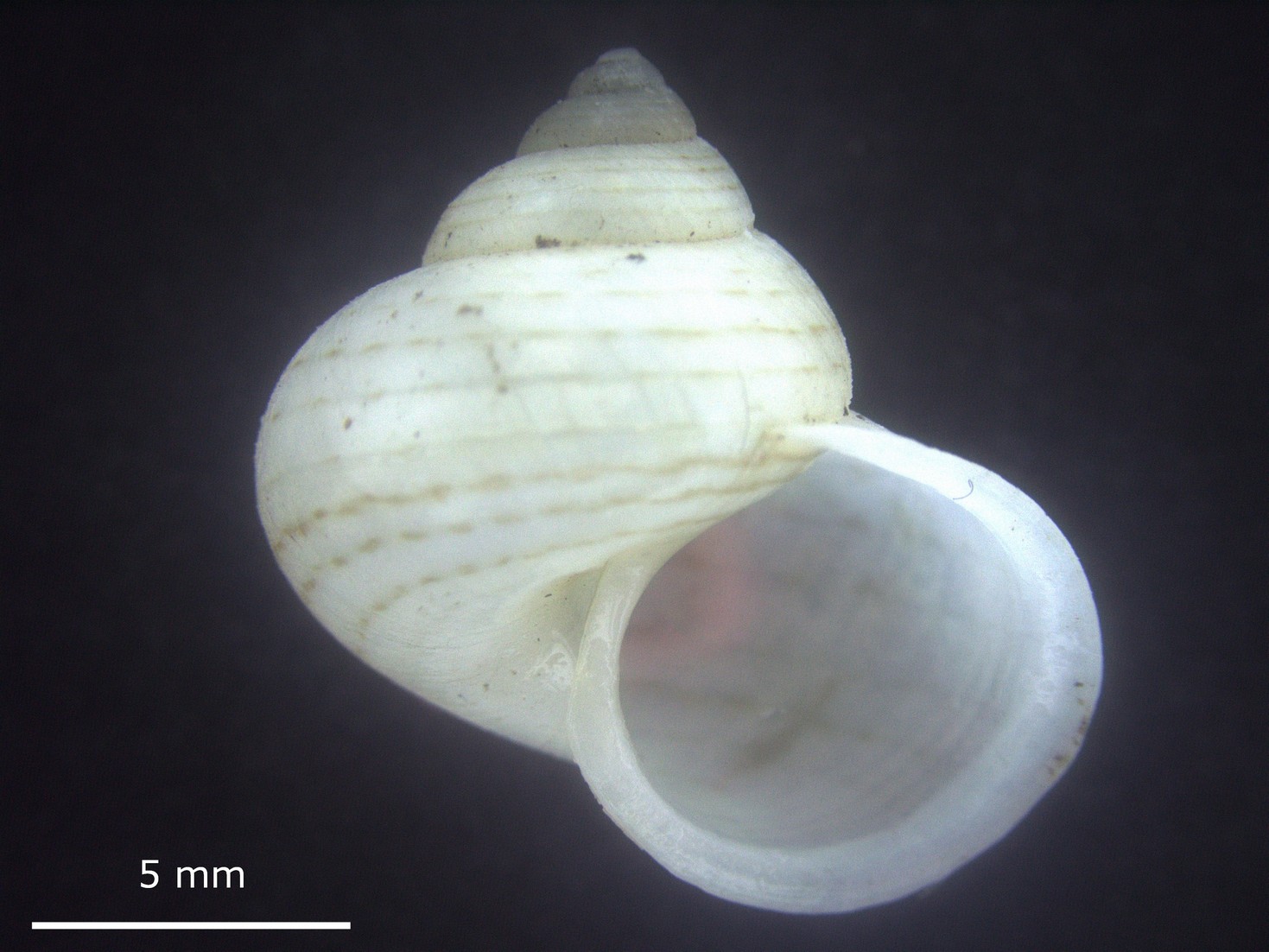

Supplement: File S4 [file peerj-10-13501-s004.zip › New Folder/9444.1.jpg]

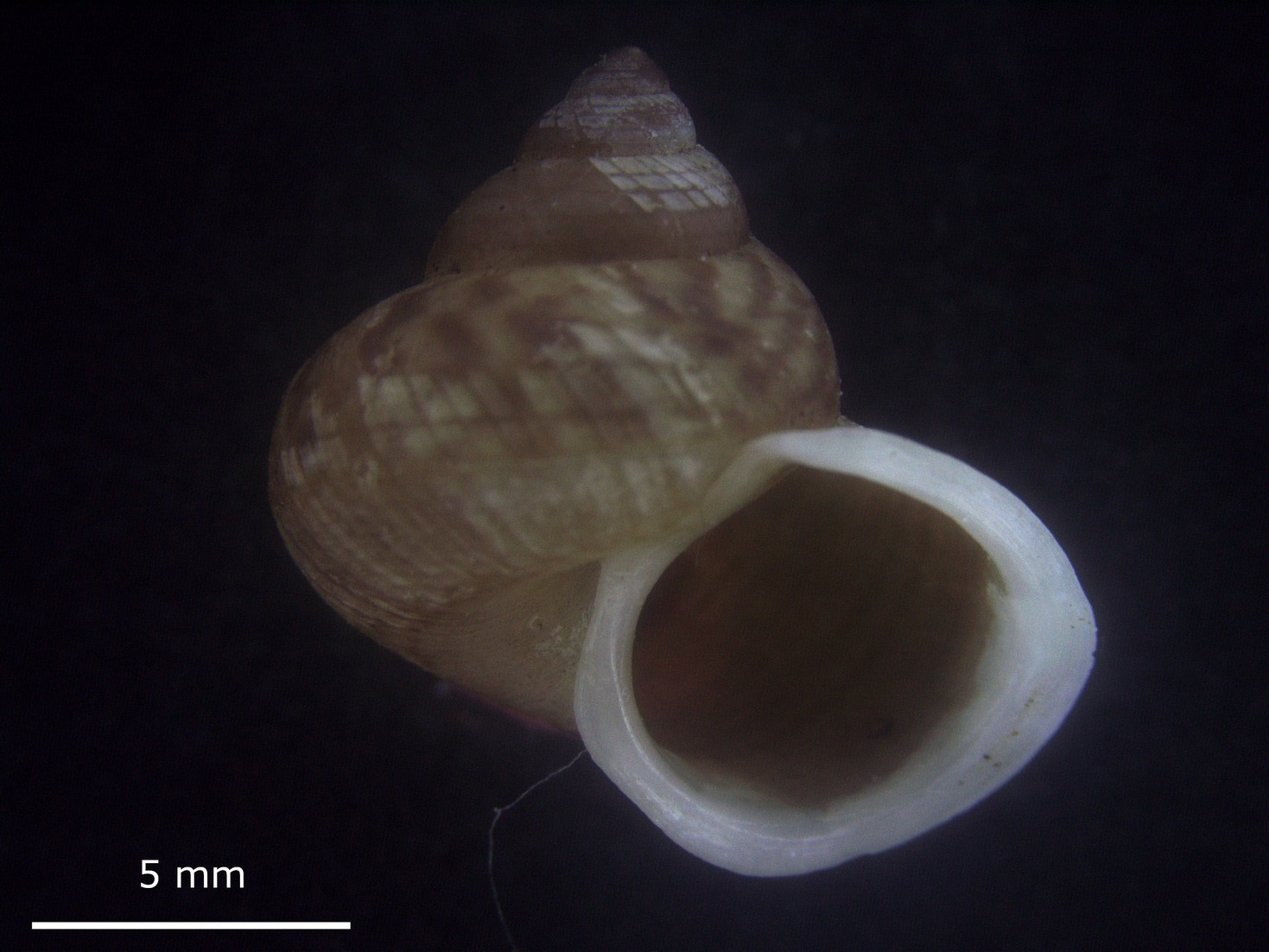

Supplement: File S4 [file peerj-10-13501-s004.zip › New Folder/9445.1.jpg]

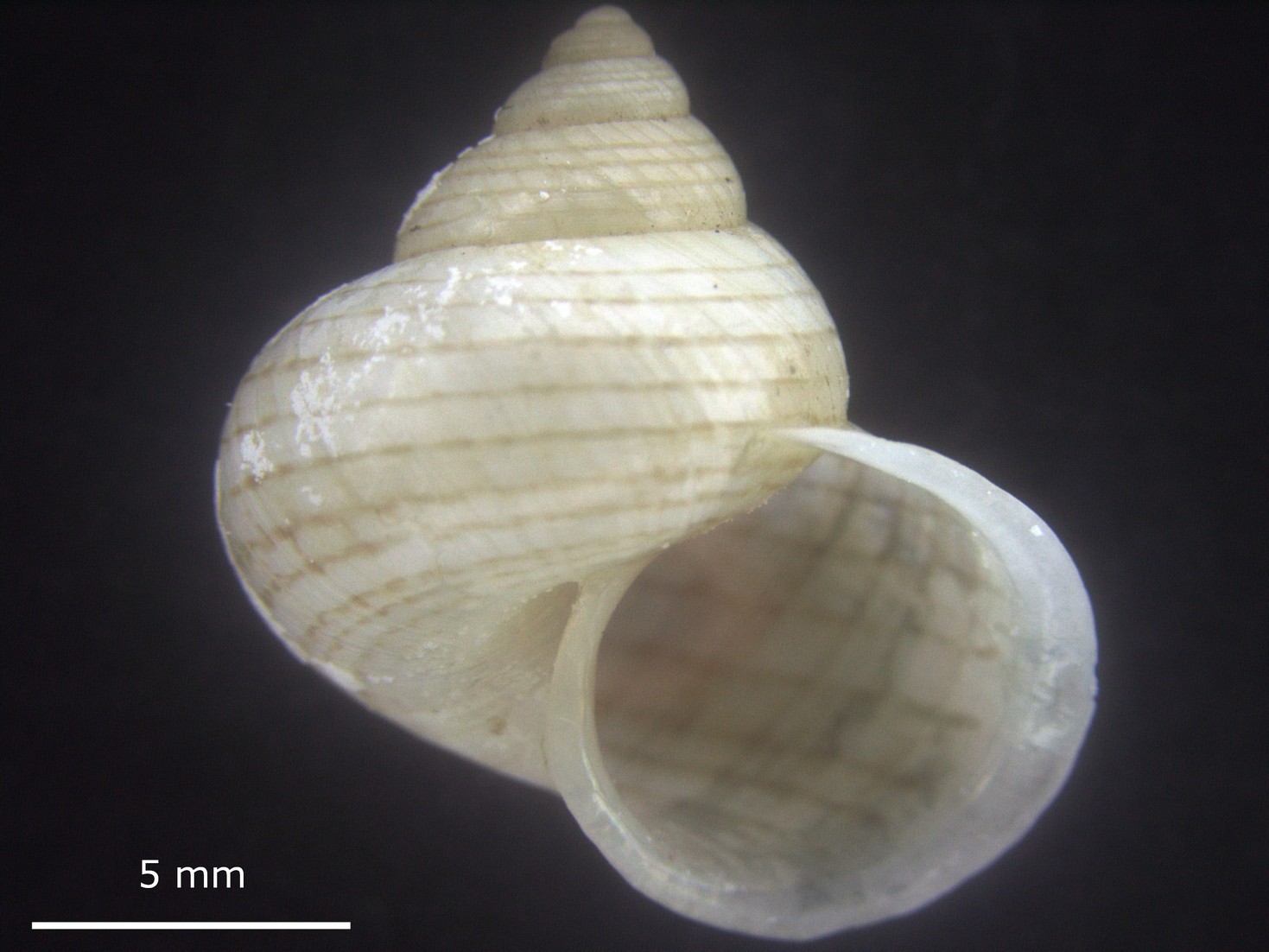

Supplement: File S4 [file peerj-10-13501-s004.zip › New Folder/9446.1.jpg]

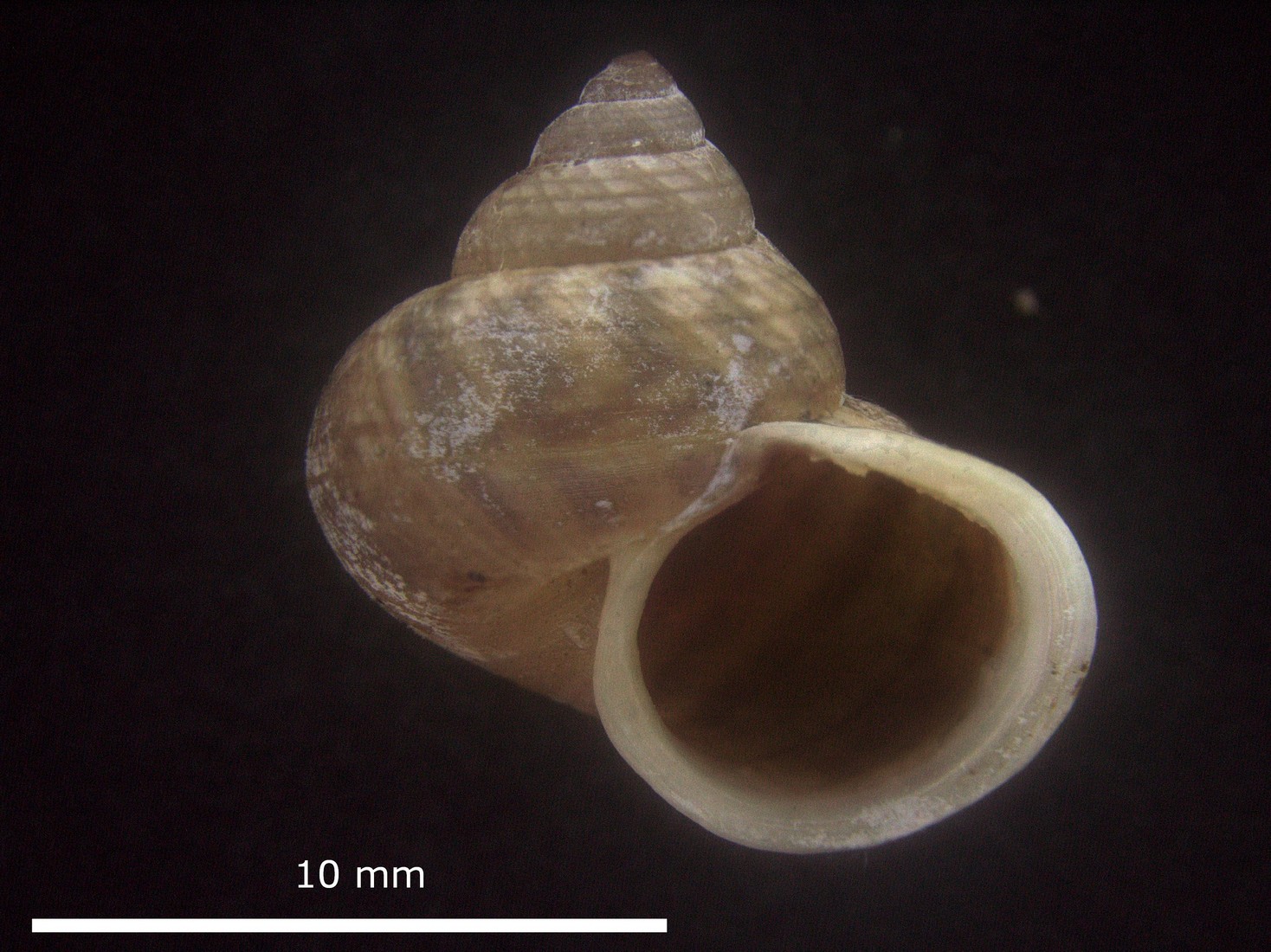

Supplement: File S4 [file peerj-10-13501-s004.zip › New Folder/9447.1.jpg]

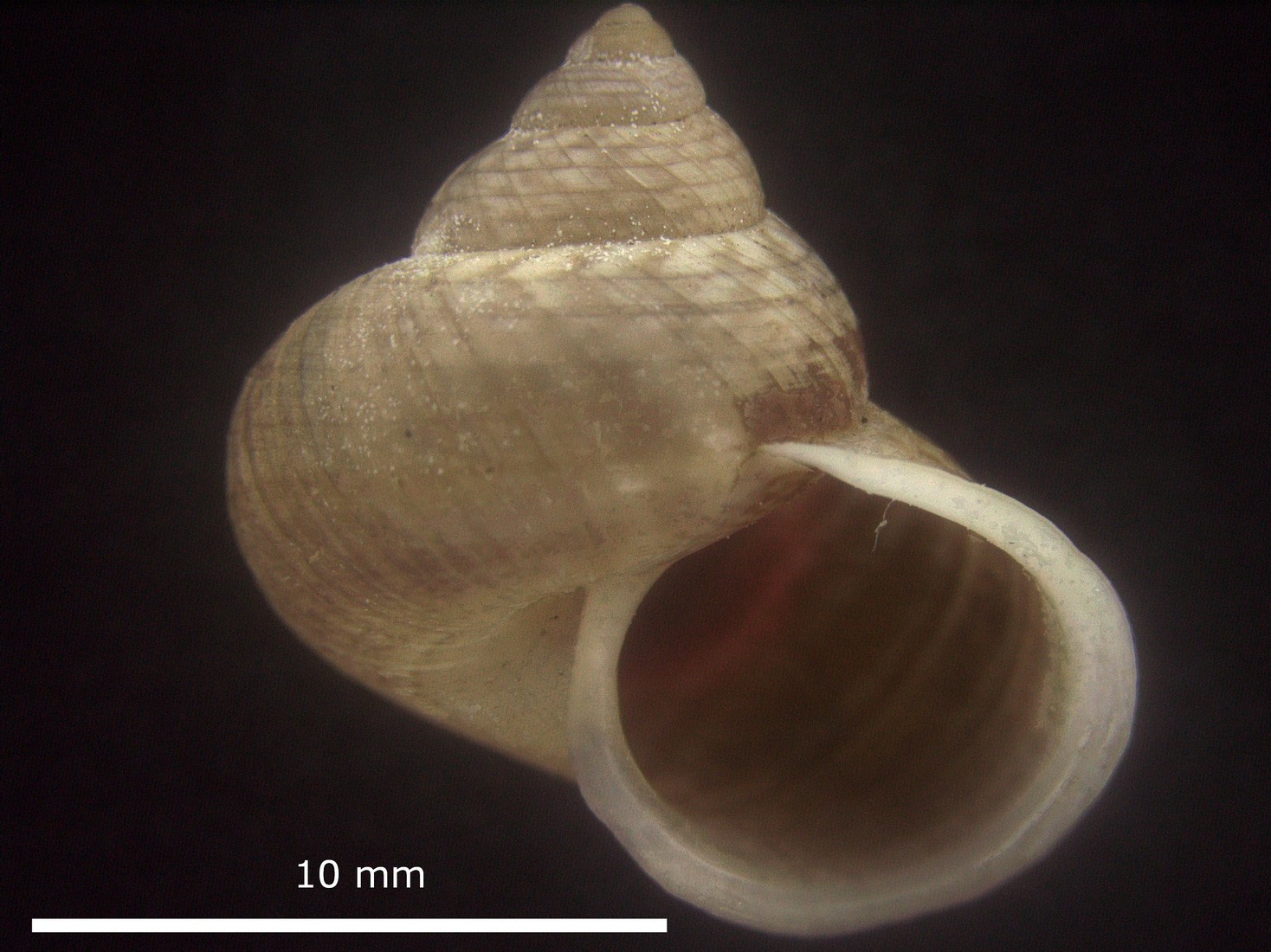

Supplement: File S4 [file peerj-10-13501-s004.zip › New Folder/9449.1.jpg]

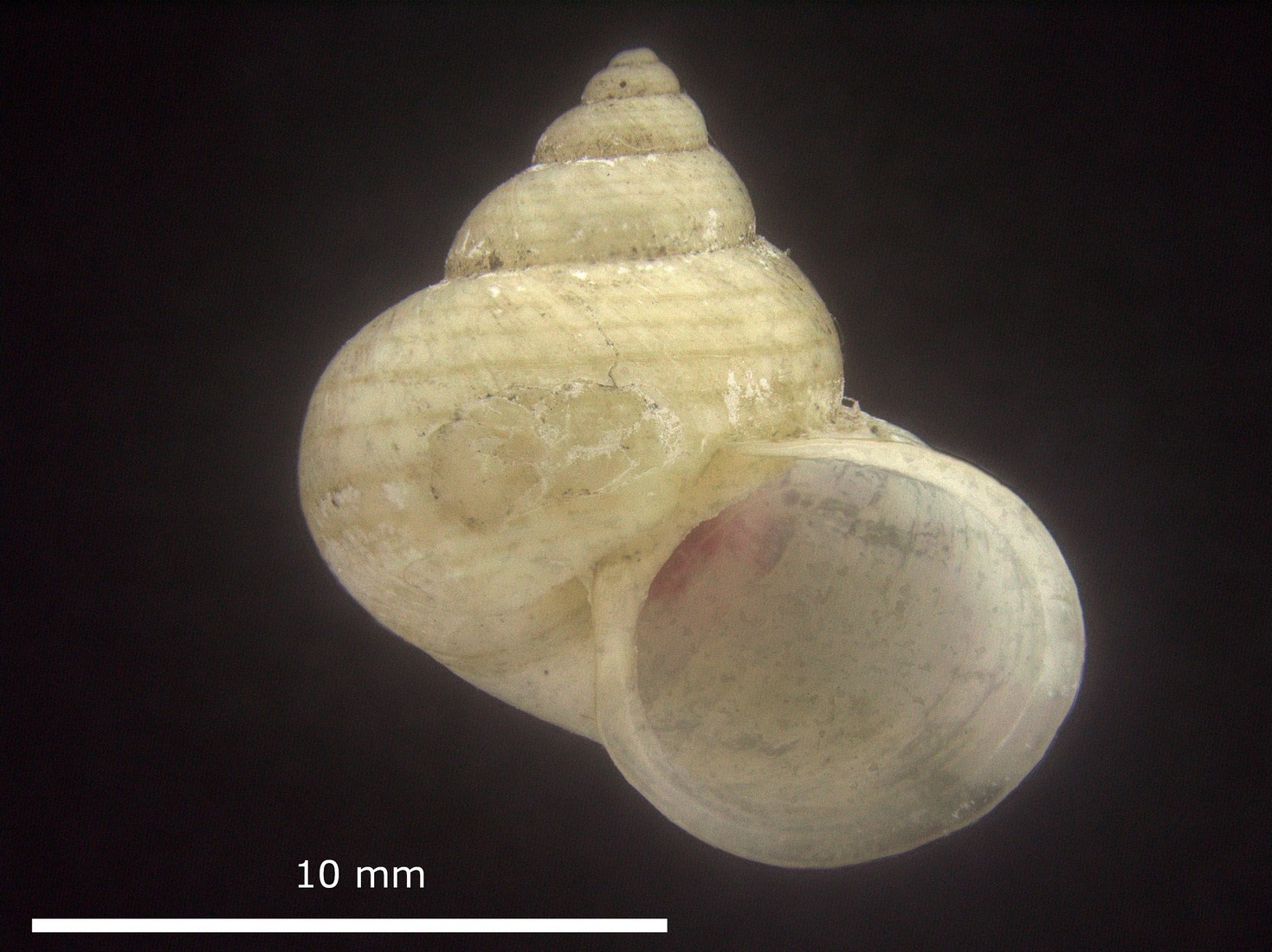

Supplement: File S4 [file peerj-10-13501-s004.zip › New Folder/9450.1.jpg]

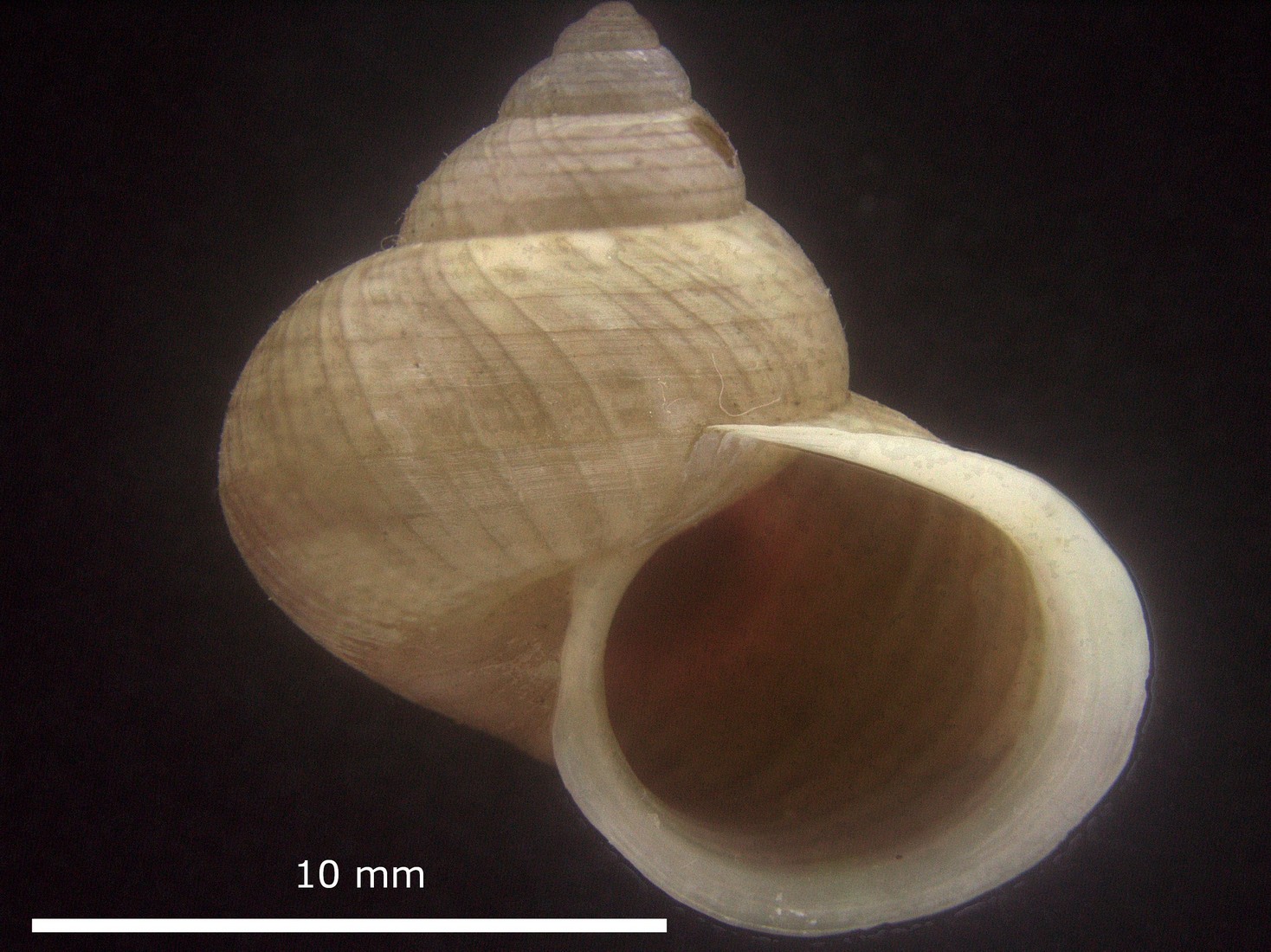

Supplement: File S4 [file peerj-10-13501-s004.zip › New Folder/9451.1.jpg]

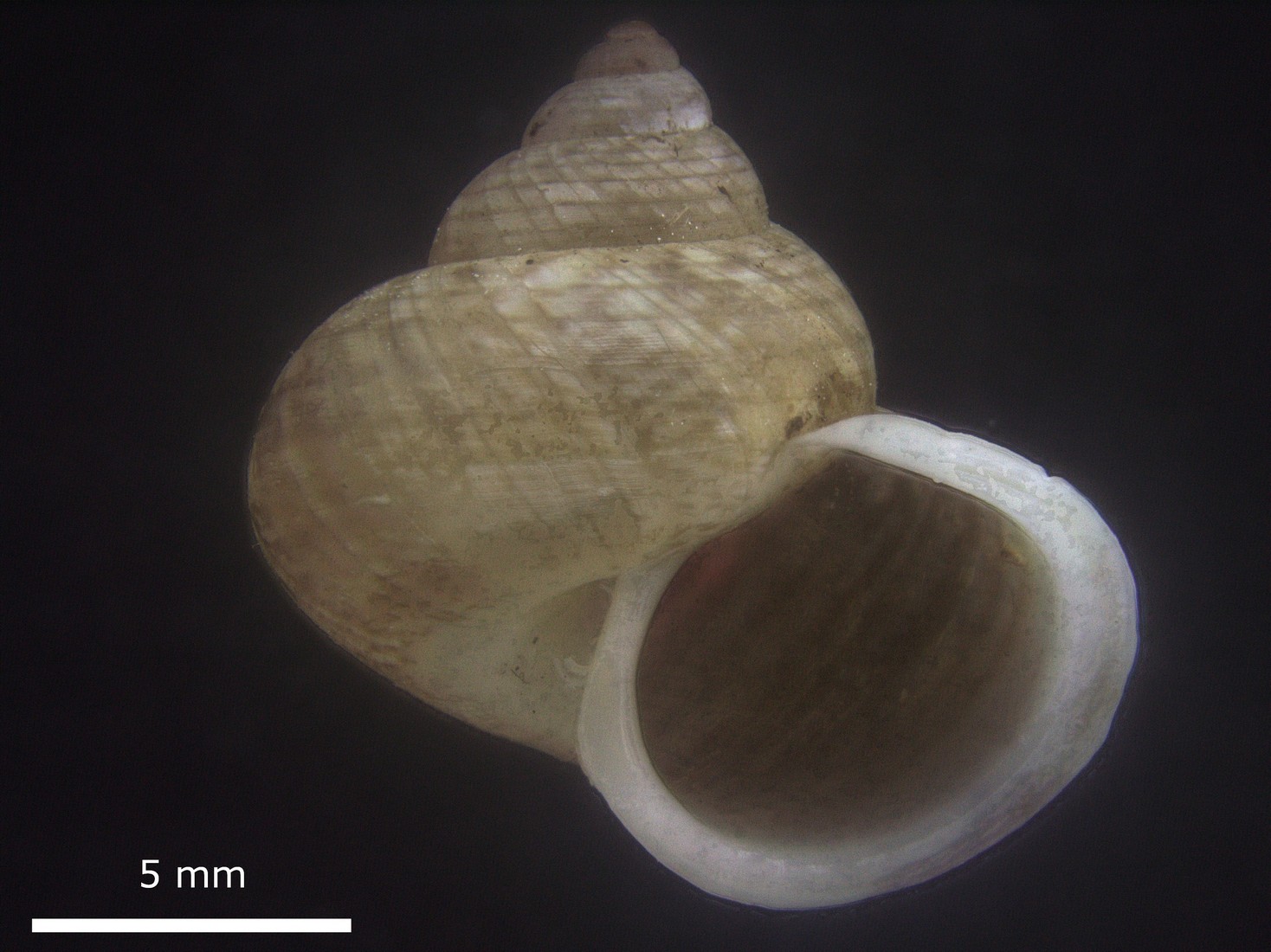

Supplement: File S4 [file peerj-10-13501-s004.zip › New Folder/9726.1.jpg]

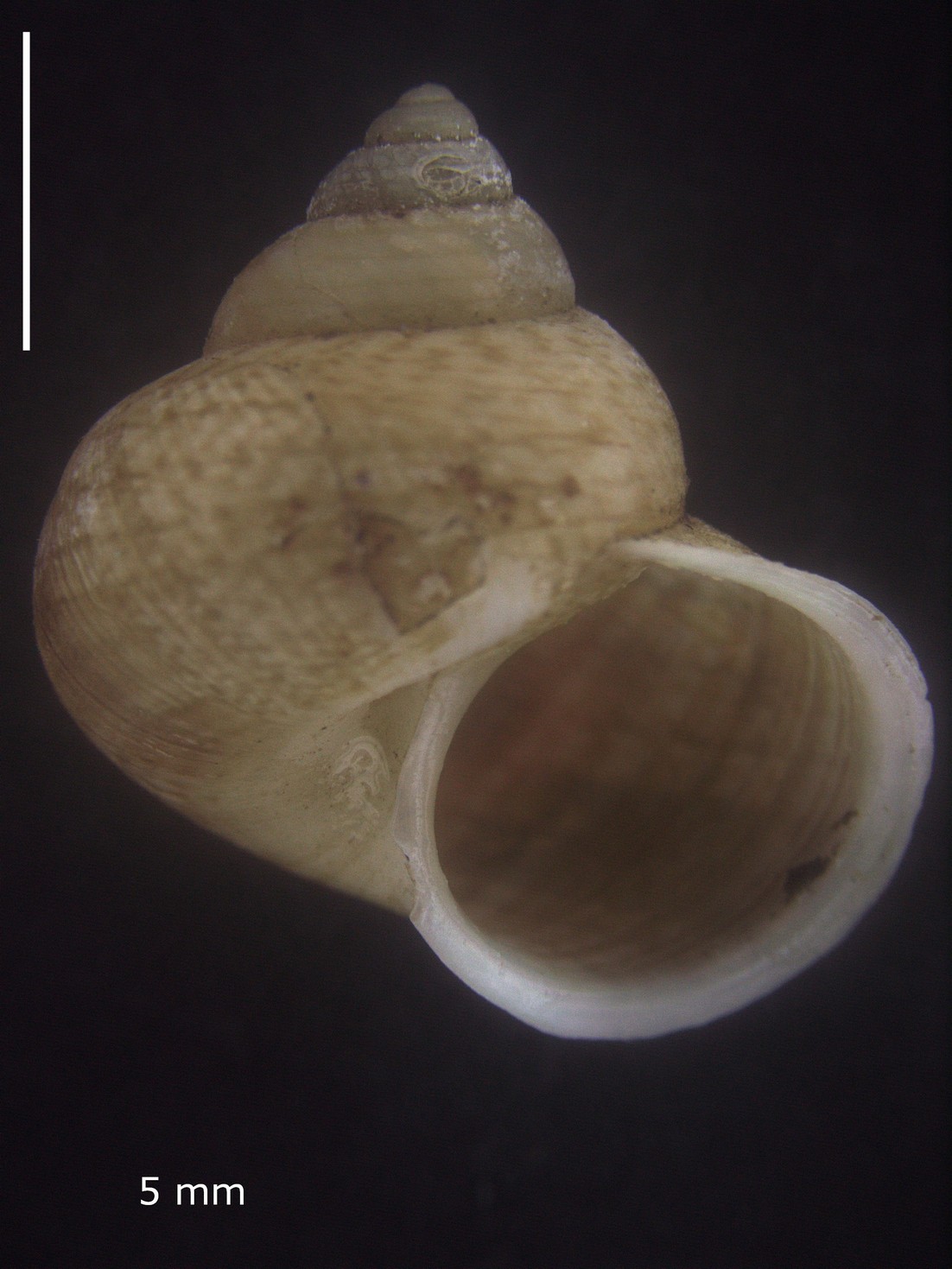

Supplement: File S4 [file peerj-10-13501-s004.zip › New Folder/9727.1.jpg]

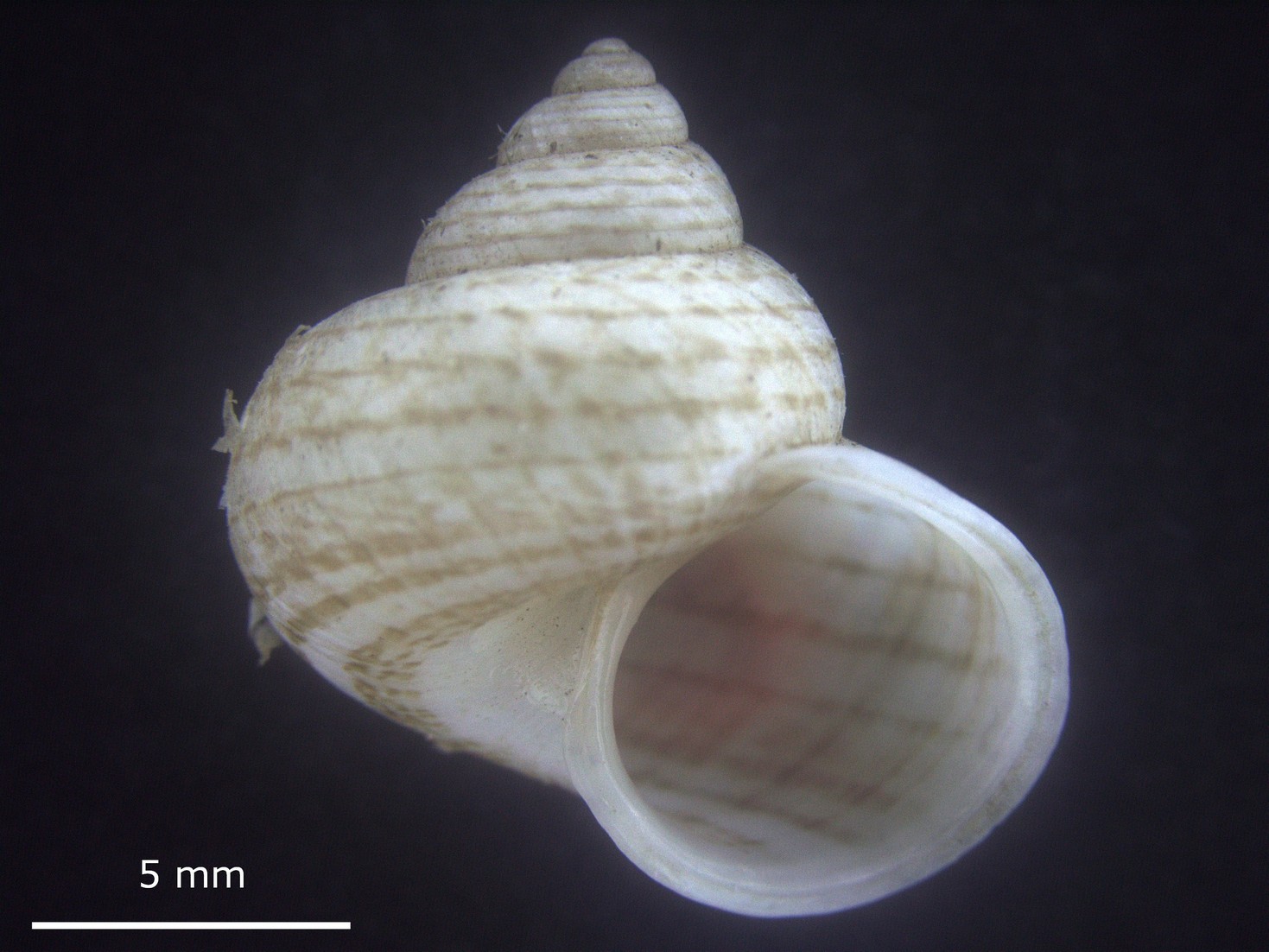

Supplement: File S4 [file peerj-10-13501-s004.zip › New Folder/9728.1.jpg]
